# Simultaneous Detection of Beta and Gamma Human Herpesviruses by Multiplex qPCR Reveals Simple Infection and Coinfection Episodes Increasing Risk for Graft Rejection in Solid Organ Transplantation

**DOI:** 10.3390/v10120730

**Published:** 2018-12-19

**Authors:** Yessica Sánchez-Ponce, Gustavo Varela-Fascinetto, José Carlos Romo-Vázquez, Briceida López-Martínez, José Luis Sánchez-Huerta, Israel Parra-Ortega, Ezequiel M. Fuentes-Pananá, Abigail Morales-Sánchez

**Affiliations:** 1 Research Unit in Virology and Cancer, Children’s Hospital of Mexico Federico Gómez, 06720 Mexico City, Mexico; yes103_neutron@hotmail.com (Y.S.-P.); empanana@yahoo.com (E.M.F.-P.); 2 Postgraduate Program in Biological Science, National Autonomous University of Mexico, 04510 Mexico City, Mexico; 3 Department of Transplantation, Children’s Hospital of Mexico Federico Gómez, 06720 Mexico City, Mexico; gvarela@himfg.edu.mx; 4 Department of Nephrology, Children’s Hospital of Mexico Federico Gómez, 06720 Mexico City, Mexico; dr.jcnefro@gmail.com; 5 Subdirection of Diagnostic Auxiliary Services, Children’s Hospital of Mexico Federico Gómez, 06720 Mexico City, Mexico; brisalopezmtz@gmail.com; 6 Department of Clinical Laboratory, Children’s Hospital of Mexico Federico Gómez, 06720 Mexico City, Mexico; jlshuerta@yahoo.com.mx (J.L.S.-H.); i_parra29@hotmail.com (I.P.-O.)

**Keywords:** human herpesviruses, transplantation, coinfection, graft rejection

## Abstract

Herpesviruses are common components of the human microbiome that become clinically relevant when a competent immunosurveillance is compromised, such as in transplantation. Members of the beta and gamma subfamilies are associated with a wide diversity of pathologies, including end-organ disease and cancer. In this study, we developed a multiplex qPCR technique with high specificity, sensitivity, efficiency and predictability that allowed the simultaneous detection and quantification of beta and gamma human herpesviruses. The technique was tested in a cohort of 34 kidney- or liver-transplanted pediatric patients followed up for up to 12 months post-transplant. Viral load was determined in 495 leukocyte-plasma paired samples collected bi-weekly or monthly. Human herpesvirus (HHV) 7 was the herpesvirus most frequently found in positive samples (39%), followed by Epstein-Barr virus (EBV) (20%). Also, EBV and HHV7 were present in the majority of coinfection episodes (62%). The share of positive samples exclusively detected either in leukocytes or plasma was 85%, suggesting that these herpesviruses tended to take a latent or lytic path in an exclusive manner. Infection by human cytomegalovirus (HCMV) and HHV6, as well as coinfection by EBV/HHV7 and EBV/HHV6/HHV7, were associated with graft rejection (RR = 40.33 (*p* = 0.0013), 5.60 (*p* = 0.03), 5.60 (*p* = 0.03) and 17.64 (*p* = 0.0003), respectively). The routine monitoring of beta and gamma herpesviruses should be mandatory in transplant centers to implement preventive strategies.

## 1. Introduction

Herpesviruses are common human residents that in most cases cause asymptomatic infection in immunocompetent people. Human cytomegalovirus (HCMV), human herpesvirus (HHV) 6A, HHV6B, and HHV7 conform the beta subfamily, while Epstein-Barr virus (EBV) and Kaposi Sarcoma Herpesvirus (KSHV) belong to the gamma subfamily. After primary infection, beta and gamma herpesviruses remain latent throughout the life of infected individuals with sporadic reactivation episodes that mostly cause no disease [1]. Nevertheless, in immunocompromised individuals such as those with pharmacological immunosuppression after organ or tissue transplantation, both primary and recurrent herpesvirus reactivation can cause a plethora of clinical effects. These clinical complications range from mild febrile episodes to end-organ disease or aggressive lymphomas and sarcomas [2].

Some reports about herpesvirus coinfections suggest a joint role influencing the course of different malignancies, such as Burkitt’s lymphoma, primary effusion lymphoma and Kaposi’s sarcoma [3,4,5]. Cooperative coinfections have also been described for EBV and HIV, *Plasmodium falciparum* and *Helicobacter pylori* [6,7]. These studies support the hypothesis of a potential viral communication among herpesviruses, which could synergize their pathogenic effects. However, in spite of the associated morbidity of beta and gamma herpesviruses in post-transplant patients, there are few studies addressing a cooperative role, with very little or no evident clinical implications [8,9,10,11,12,13].

Assessing the clinical–biological significance and potential cooperative capacity of beta and gamma herpesviruses to cause or worsen disease demands systematic and simultaneous quantitative monitoring. The multiplex and quantitative polymerase chain reaction (qPCR) using Taqman probes allows the evaluation of viral loads present in biological specimens [14]. This technique saves time and the amount of sample and reagents needed for the analysis, which considerably reduces economic and biological costs [15]. Quantitative and multiplex analyses of beta and gamma herpesviruses are scarce [16,17]. Here, we describe a standardized qPCR to quantitatively and simultaneously detect beta and gamma herpesviruses. The technique was tested in a cohort of 34 pediatric patients included in the liver and kidney transplant program at the Children’s Hospital of Mexico “Federico Gómez”. Blood samples (*N* = 495) were prospectively obtained from patients biweekly or monthly until completing up to 12 months of follow-up. We evaluated episodes of single and multiple infection as well as episodes of viral persistence during consecutive sampling. Also, we analyzed viral loads from plasma and leukocytes separately as an indirect indicator of latent or lytic infection. Finally, we analyzed the association of infections and coinfections detected with graft rejection.

## 2. Materials and Methods

### 2.1. Viruses

HCMV strain AD169 (ATCC VR538) was used to infect primary human foreskin fibroblasts and viral genomes were obtained from the culture supernatant. HHV6 DNA was obtained from the MOLT3 cell line (ATCC CRL-1552) infected with viral particles of HHV6B Z29 strain (ATCC VR-1467). HHV7 DNA was purified from primary infected lymphocytes. EBV and KSHV genomes were obtained from the commercial cell lines Raji (ATCC CCL-86) and BCP-1 (ATCC-CRL-2294), respectively.

### 2.2. Primers and Probes

We used primers and probes previously described in the literature or designed in our laboratory to PCR amplify viral targets (Table 1). The targeted genes were: EBV *Balf*-*5*, HCMV *UL123*, HHV6 *U31*, HHV7 *U57* and KSHV *Lana*. These genes were chosen because they share no homology with other herpesviruses, with the exception of HHV6 for which the designed primers recognize both HHV6A and HHV6B (mentioned throughout the text only as HHV6). We also included a pair of primers and a probe to detect endogenous β-actin, which was used as an internal amplification control. We analyzed several parameters for primers and probes previously reported, including length, melting temperature (Tm) and percentage of GC base pairs (Oligo Analyzer 3.1 Software [18]).

We considered all relevant parameters for de novo design of primers and probes (Primer-BLAST [22]) in order to homogenize characteristics of all oligonucleotides. Also, we carefully analyzed the formation of self-dimers and hetero-dimers for all combinations of primers and probes that would be present in the same reaction tube in order to rule out the possibility of formation of inappropriate oligonucleotide pairing that could interfere with the specific reactions (Supplementary Tables S1–S4). The Tms were in the range of 53.3 °C to 58.6 °C for primers, while Tms were in the range of 55.1 °C to 66.5 °C for probes. We used probes targeting EBV and HHV6 labeled with the 6-Carboxyfluorescein (FAM) fluorophore, HCMV and HHV7 probes with 6-Carboxi-4′,5′-Dichloro-2',7'-Dimethoxyfluorescein, succinimidyl ester (JOE) fluorophore, and β-actin and KSHV probes with Cyanine 5 (Cy5) fluorophore. All primers and probes were commercially synthesized (Synthetic DNA S.A.P.I. of C.V.).

### 2.3. Construction of Plasmids Carrying Viral Targets

Viral targets were PCR amplified using primers described in Table 1 and cloned individually into the commercial vector pGEM-T Easy (Promega, Madison, WI, USA), according to the manufacturer’s instructions. Plasmids containing viral fragments were digested with restriction enzyme *EcoR*I (New England BioLabs, Ipswich, MA, USA) to release the insert. The expected size of every viral fragment was confirmed by agarose gel electrophoresis and then identified by Sanger sequencing. Plasmids containing viral fragments were also digested with restriction enzyme *Nde*I (New England BioLabs) to produce linear plasmids, which were then used in the multiplex qPCRs. From here, these plasmids containing viral fragments will be mentioned throughout the text as standard plasmids.

### 2.4. Multiplex qPCR Standardization

qPCRs were performed with a Rotor-Gene Q 5PLEX HRM (Qiagen, Hilden, NRW, Germany) and analyzed in Rotor-Gene Q Software 2.3 (Qiagen, Hilden, NRW, Germany). We first tested specificity of the primers in a background of human genomic DNA using DNA from the EBV, HCMV, HHV6, HHV7 and KSHV negative human cell line MOLT3 (ATCC-CRL-1552). We ran every PCR individually using Sybr Green (Thermo Fisher, Waltham, MA, USA) as source of fluorescence. Serial dilutions (10^6^–10^1^) of standard plasmids were mixed with 100 ng of the MOLT3 cell line. We confirmed the amplification of a single product in every reaction with the MELT curves (dissociation curves) and then the expected size of every product was visualized by 1.5% agarose gel electrophoresis.

We standardized a two-tube multiplex qPCR as follows. EBV, HCMV and β-actin were detected in one tube and HHV6, HHV7 and KSHV were detected in another tube. Both multiplex qPCRs were designed with identical cycling conditions for simultaneous analysis. The multiplex qPCR assays were performed in a final volume of 20 μL containing: 1× QuantiTect Multiplex PCR NoRox (Qiagen, Hilden, NRW, Germany), 250 nM of each forward and reverse primer (except for primers targeting β-actin, which were used at 125 nM), 125 nM of each probe (except for β-actin, which was used at 62.5 nM) and 5 μL of the appropriate mix of standard plasmids. Standard curves were constructed using 10-fold serial dilutions (10^6^–10^1^) copies of viral plasmids. Every standard also contained 100 ng of DNA from the human cell line MOLT3 (ATCC-CRL-1552) as background DNA. The addition of this human genomic DNA allowed us to simulate the conditions under which the reaction would occur in biological samples, since the viral genomes would be in a cellular DNA background. Non-template controls were included in every run. Each qPCR assay was run in triplicate. The cycling conditions were the following: a uracil-DNA glycosylase (UDG) incubation step at 52 °C for 2.5 min, an initial denaturation and polymerase activation step at 95 °C for 15 min, followed by 50 cycles of denaturation at 95 °C for 15 s and annealing/elongation at 60 °C for 1 min.

Efficiency (E), predictability (R^2^) and limit of detection (LOD) were determined from 17 standard curves run in triplicate (*N* = 51) (see Section 2.8 “Statistical Analysis” for a most detailed explanation). We selected 17 as a high number of standard curves that would allow us to reduce the variability of individual assays and thus to report the parameters of the qPCR with high reliability. Repeatability (intra-assay variation of replicates) and reproducibility (inter-assay variation of replicates) were calculated for each standard (10^6^–10^1^) of the curves. Repeatability was obtained from the replicates of each qPCR assay (*N* = 3) and reproducibility was derived from qPCR assays run independently (*N* = 9). Viral detection was carried out in individual and multiplex qPCR reactions. Individual qPCR assays were performed under identical conditions as the multiplex assays, except that only one set of primers/probe was included. We used the coefficient of correlation (R) to evaluate the correlation between the number of copies we could detect in simple vs. multiplex assays.

Cross-reactivity assays were performed using 10^4^–10^6^ complete viral genomes of each virus as template instead of the standard plasmids (containing only a short viral fragment). We used the same conditions of the two-tube multiplex qPCR, including the same mix of primers and Taqman probes. In this manner, we tested whether the sets of primers/probes were specific for the designed virus. The sources of viral genomes are described in Section 2.1 “Viruses”.

### 2.5. Patients and Clinical Samples

This study was approved by the Ethical, Biosecurity and Scientific review boards of the Children’s Hospital of Mexico Federico Gómez (Registry HIM-2016-021). Clinical data were obtained from medical records. Graft rejection was diagnosed from clinical, laboratory and histopathological data. Prior to sample collection, patients and their parents/guardians were informed about the nature of the study and those who were willing to participate signed a letter of consent (parents/guardians) and a letter of assent (children older than 10 years). Children with incomplete follow-up or suffering hyperacute graft rejection were excluded from the study, but represented less than 15% of the cohort. All enrolled patients were treated according to the ethical guidelines of our institution. Thirty-four pediatric patients who received kidney or liver transplants between February 2016 and August 2017 were included. The follow-up time was 12 months for 28 patients and 8 months for 5 patients. One patient was only followed-up for 1.5 months because of loss of the transplanted organ after acute graft rejection. Blood samples (*N* = 495) were prospectively obtained from subjects biweekly (+/− 8 days) during the first three months post-transplant and monthly (+/− 10 days) thereafter, according to the patient’s requested medical check-ups. A pre-transplantation sample was also collected for all patients just before surgery, adding up to a total of between 4 and 17 peripheral blood samples collected per patient (median 15). A quantity of 2–4 mL of peripheral blood was collected by venous puncture in EDTA tubes (BD Vacutainer, Franklin Lakes, NJ, USA). Blood was centrifuged and plasma was aliquoted and frozen until use. Leukocytes were purified after lysing erythrocytes with buffer EL (Qiagen, Hilden, NRW, Germany) and frozen until use.

### 2.6. Viral Detection in Clinical Samples

DNA was purified from 2–4 × 10^6^ leukocytes using QIAamp DNA Mini Kit (Qiagen, Hilden, NRW, Germany) according to manufacturer’s instructions. Quantification was done in a nanodrop 1000 spectrophotometer (Thermo Fisher Scientific). The purity and integrity of DNA were evaluated through optical density (260/280 ratio), 1.5% agarose gel electrophoresis and amplification of the β-actin endogenous gene. Viral detection was conducted using 100 ng of DNA and then expressed as copy number per µg of DNA (Conversion factor = 10). We used 200 µL (1/5 of 1 mL) of plasma for DNA purification using QIAsymphony virus/bacterium mini kit (Qiagen, Hilden, NRW, Germany) in QIAsymphony SP equipment according to the manufacturer’s instructions. DNA was eluted in 60 µL of elution buffer of the kit. Virus detection was conducted in 5 µL (1/12 of the eluate) and then expressed as copy number per ml of plasma (conversion factor = 60 (12 × 5)). A total of 495 paired samples (leukocytes-plasma) were tested by triplicate with the two-tube multiplex qPCRs. The number of viral copies per sample was calculated from the standard curves and multiplied by the corresponding conversion factor to be reported throughout the text as viral copies per μg of DNA (from leukocytes) or per mL of plasma.

### 2.7. Sanger Sequencing

We used Sanger sequencing to confirm the identity of cloned viral fragments and the PCR products of each virus of the first positive in clinical samples. Both forward and reverse strands were sequenced and results were blasted at the NCBI web site confirming their viral identity. Sequencing was carried out at the Institute of Cellular Physiology, Nacional Autonomous University of Mexico.

### 2.8. Statistical Analysis

GraphPad Prism 7 and Excel Software were used to construct graphs and perform most of data analysis (except where it is specified). Venn diagrams were constructed using a free online tool (http://bioinformatics.psb.ugent.be/webtools/Venn/). The coefficient of correlation (R^2^) was used to evaluate linearity of standard curves in relation to input of control plasmid and the cycle threshold (Ct) value. A regression analysis using Pearson correlation coefficient (R) compared the multiplex vs. simplex assays. For evaluation of the multiplex qPCRs’ performance, 51 measurements obtained from 17 standard curves run in triplicate (17 × 3) were analyzed. Efficiency (calculated from slope M) and predictability (coefficient of determination R^2^) were obtained directly from the software Rotor-Gene Q Software 2.3. We only calculated the mean of these values from the 17 assays. Limits of detection (LOD) were determined from the minimum number of copies (10 in our standard curve) that could be amplified with a 95% confidence. The mean of 51 measurements was calculated and added to 1.96 times its standard deviation: LOD = x × 1.96σ (1.96 was obtained from the Standard Normal Distribution z-Table) [23]. Repeatability and reproducibility were calculated for each standard (10^6^–10^1^) of the curves using the coefficient of variation of Ct values: CV = (σ/x) × 100. An adequate coefficient of variation must be below 5% (95% confidence) [24]. The Chi-squared test and Fisher’s exact test were used to analyze the significance of the association of infection to a particular blood fraction (leukocytes and plasma). The Chi-squared test and Fisher’s exact test were calculated using a free online tool (https://www.socscistatistics.com/Default.aspx). The relative risk (RR) analysis was calculated using the free online MedCalc statistical software. RR was calculated from a 2 × 2 cross-tabulation in which the following variables were contrasted: exposition (simple infection/coinfection, any combination found) and outcome (allograft rejection). For each day in which we tested a blood sample, we investigated whether the patient had been diagnosed with allograft rejection according to the medical records. Infection and rejection were both considered as categorical dichotomous variables. We established the presence/absence of infection for every sample of every patient (presence: single or coinfection without considering the magnitude of viral load nor the blood fraction, leukocytes or plasma) and the presence/absence of allograft rejection. The RR represents the ratio of rejection episodes co-presenting with infection/coinfection vs. rejection episodes with absence of infection.

## 3. Results

### 3.1. Specificity of the Primers on a Human DNA Background

**Figure 1:**
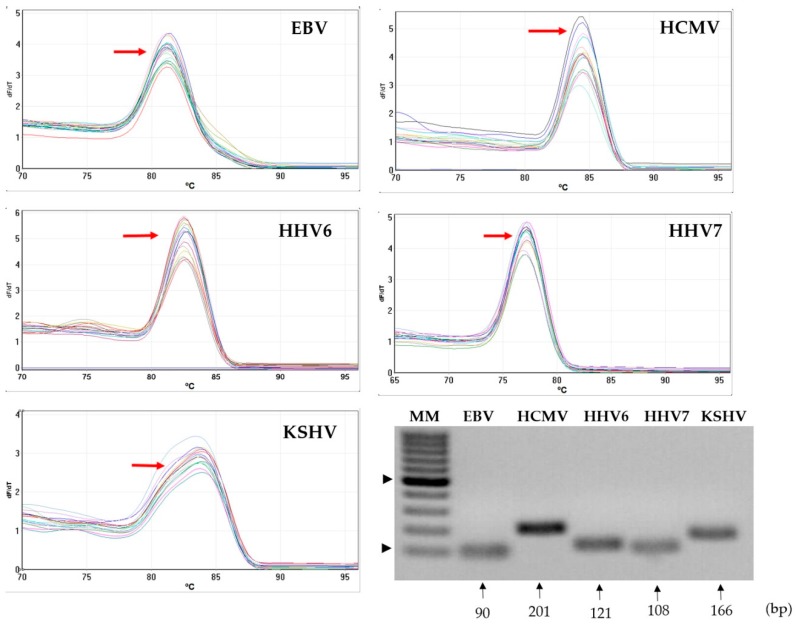
Assessment of specificity of primers in a human DNA background. MELT curves from each Sybr Green-based PCR detected the indicated virus. A single peak of dissociation (red arrow) was observed from each individual reaction ran in the presence of human genomic DNA from the MOLT3 cell line. Agarose gel electrophoresis (bottom right) showed that a unique PCR product of the expected size was amplified in each individual Sybr green-based PCR. MM: 100 bp (base pairs) molecular weight marker. Black arrowheads point at bands corresponding to 100 bp and 500 bp.

To develop the multiplex qPCR for the simultaneous detection of EBV, HCMV, HHV6, HHV7 and KSHV, we first ran every reaction individually using Sybr green (Thermo Fisher Scientific). Serial dilutions of standard plasmids (10^6^–10^1^) were mixed with human genomic DNA from the cell line MOLT3 (which is negative to all studied viruses), and this mix was used as template of the qPCRs. Specificity of primers was initially evaluated by a dissociation analysis using MELT curves. Dissociation kinetics showed a single peak between 80 °C and 85 °C for each individual PCR (Figure 1). The presence of a single product of the expected size was confirmed by agarose gel electrophoresis (Figure 1). These results indicated that primers were not amplifying nonspecific products in the human genomic DNA background.

### 3.2. Performance of the Multiplex qPCRs

We standardized a two-tube multiplex qPCR using specific fluorescent probes. EBV, HCMV and β-actin were detected in one tube and HHV6, HHV7 and KSHV were detected in another tube. Serial dilutions of standard plasmids (10^6^–10^1^) were mixed with human genomic DNA from the MOLT3 cell line (negative to all viruses studied) and used as template.

**Figure 2:**
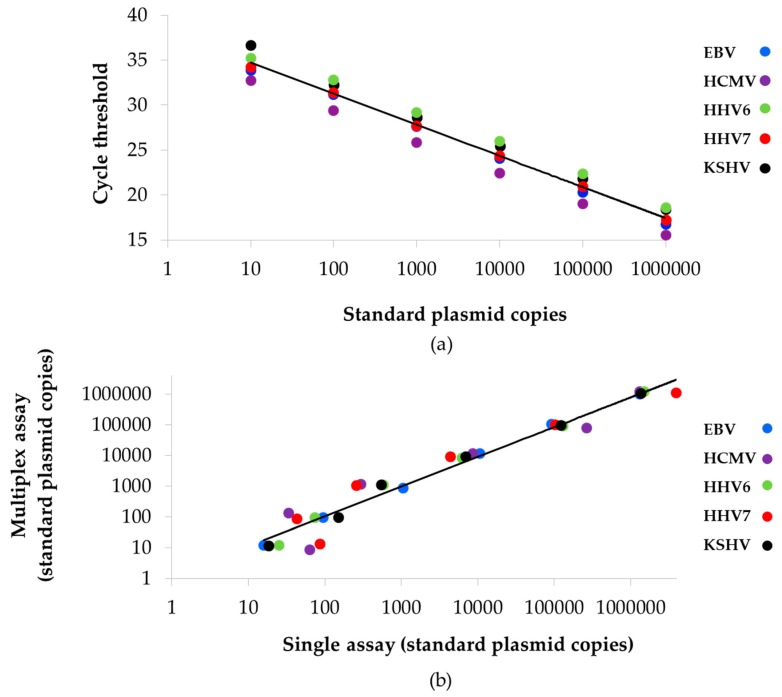
Standardization of the two-tube multiplex qPCR. (**a**) Serial dilutions of standard plasmids (10^6^–10^1^ copies) were used to construct standard curves. The number of copies detected for each standard plasmid dilution was plotted against the average of the cycle threshold. Efficiencies and coefficient of determination R^2^ values (predictability) are shown in Table 2. (**b**) Correlation of viral copy numbers detected in multiplex vs. single assays. Correlation coefficients (R) are shown in Table 2.

We ran 17 standard curves in triplicate (*N* = 51) to evaluate the performance of the multiplex qPCR. The dynamic range of multiplex qPCR comprised six orders of magnitude, with a strong linear relationship between input of standard plasmid and cycle threshold (Figure 2a) with coefficients of determination (R^2^) of 0.989–0.998 (Table 2). Efficiencies were also high, ranking between 92% and 96%, and LODs were between 18 and 25 copies (Table 2). When we contrasted copy numbers detected in single vs. multiplex assays we found that correlation coefficients (R) were greater than 0.99, with values of *p* < 0.001 for all viral targets (Figure 2b and Table 2). These results indicated that there were not quantitative changes between multiplex and individual assays.

The precision of each viral qPCR was obtained from the coefficient of variation for each standard plasmid throughout all serial dilutions. Both intra-assay repeatability and inter-assay reproducibility were evaluated. The highest variability was found with the lower standard (10^1^). Even so, the variability was ≤3.87%, further indicating that the technique was highly accurate (Table 3). These results supported the assumption that the standard curves could accurately predict the number of viral copies of beta and gamma herpesviruses in biological samples.

### 3.3. Cross-Reactivity Test

**Figure 3:**
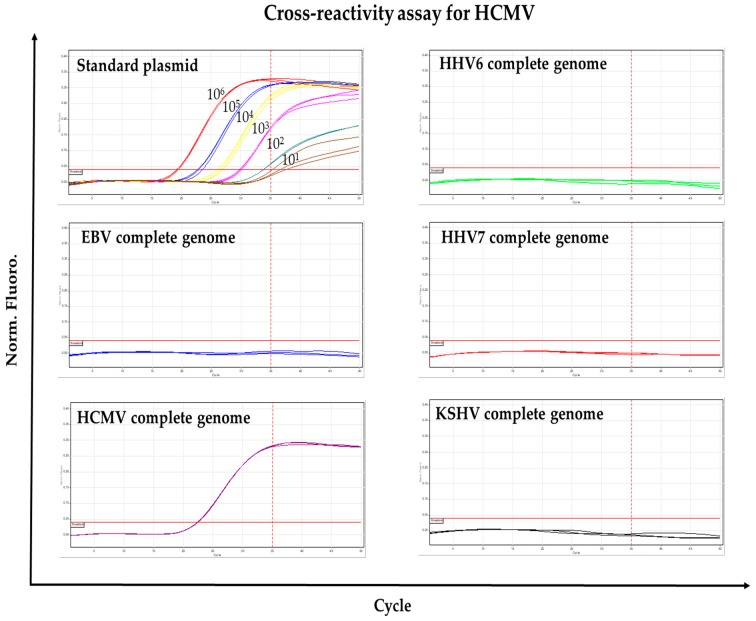
Cross-reactivity assay for HCMV. The primers and fluorescent probe used for detecting HCMV were tested for cross-reactivity with the other viruses (as is indicated in the top of each panel). Positive fluorescent signal was only detected when HCMV DNA or serial dilutions (10^6^–10^1^) of HCMV standard plasmid were used as template. For all panels, amplification cycle (Cycle) is shown in the X axis, while the normalized fluorescence (Norm. Fluoro.) is shown in the Y axis. Horizontal red lines indicate the Cycle Threshold. Dotted vertical red lines indicate the LOD. Similar results were obtained for EBV, HHV6, HHV7 and KSHV (Supplementary Figure S1).

To rule out cross-reactivity, the same sets of primers/probes used in the two-tube multiplex qPCR were tested for reactivity with DNA (complete genome) of each virus individually. No set of primers/probes showed reactivity (seen by fluorescence detection) to any nonspecific viral DNA indicating that cross-reactivity did not occur (Figure 3 and Supplementary Figure S1).

### 3.4. Detection of Beta and Gamma Herpesviruses in Pediatric Transplanted Patients

#### 3.4.1. Analysis of Single and Multiple Infections

Because pharmacologically immunosuppressed post-transplant patients are very susceptible to beta and gamma herpesvirus primo-infection and reactivation, these patients would benefit from the routine monitoring of these viruses. The Children’s Hospital of Mexico Federico Gómez is one of the pioneers and leader reference centers for pediatric transplantation in the country, with three transplant programs for solid organs (kidney, liver and heart), and one for hematopoietic progenitor cells. We applied the multiplex qPCRs to a cohort of 34 pediatric patients, transplanted with solid organs during the period between February 2016 and August 2017. Demographic and clinical characteristics of the patients are shown in Table 4. Peripheral blood samples were collected for up to 12 months (see Section 2 “Materials and Methods”). In total, 495 leukocytes and plasma paired samples were analyzed, with a median of 15 samples per patient, including a pre-transplant sample collected the same day of surgery.

Only samples whose viral loads exceeded the LODs indicated in Table 2 were considered positive. Viral detection was conducted from 100 ng of DNA of leukocytes or 5 µL of plasma, and then the results were expressed as copies per 1 µg of DNA from leukocytes or copies per 1 mL of plasma (conversion factors = 10 and = 60 for leukocytes and plasma, respectively, see Section 2 “Materials and Methods”).

**Figure 4:**
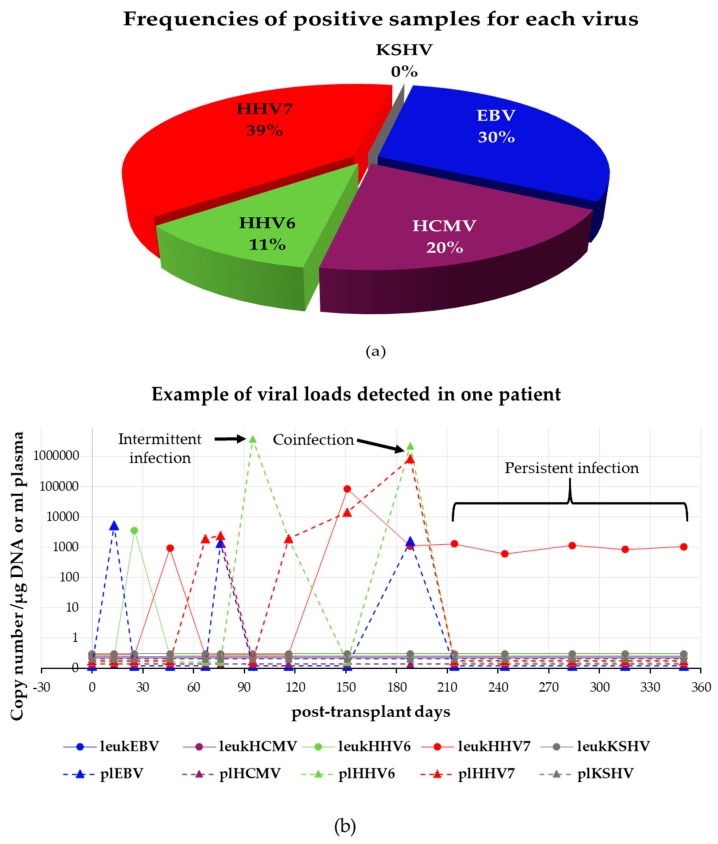
Detection of beta and gamma herpesviruses in pediatric transplanted patients. (**a**) Distribution of viral positivity considering leukocytes and plasma samples together (*N* = 171). (**b**) Example of a patient’s dynamics of infection. The follow-up period is shown in the X axis. Samples of day 0 were collected on the same day of transplant just before the surgery. Viral loads determined as the copy number/μg of DNA (leukocytes) or copy number/mL (plasma) are shown in the Y axis. This patient was chosen because presented intermittent, persistent and coinfection episodes as indicated with arrows and bracket. Viruses are color-coded. Infections detected in leukocytes (leuk) are drawn as continuous lines while those detected in plasma (pl) are drawn as dotted lines. Horizontal lines below one viral copy indicate that virus was undetected.

A total of 29 patients (85%) tested positive in at least one of their samples to at least one of the herpesviruses analyzed. Of 495 samples, 125 were positive for one or more viruses, in one or both blood fractions tested. In total, 171 viral measurements were positive, of which 67 were positive to HHV7, 51 to EBV, 35 to HCMV and 18 to HHV6. KSHV was never detected. Figure 4a shows the frequency of positive samples for each virus considering leukocytes and plasma together. Figure 4b depicts an example of the type of infection episodes observed in one patient. The kinetics of infection of the rest of the patients with positive samples are shown in Supplementary Figure S2 (kidney-transplanted patients) and Figure S3 (liver-transplanted patients).

**Figure 5:**
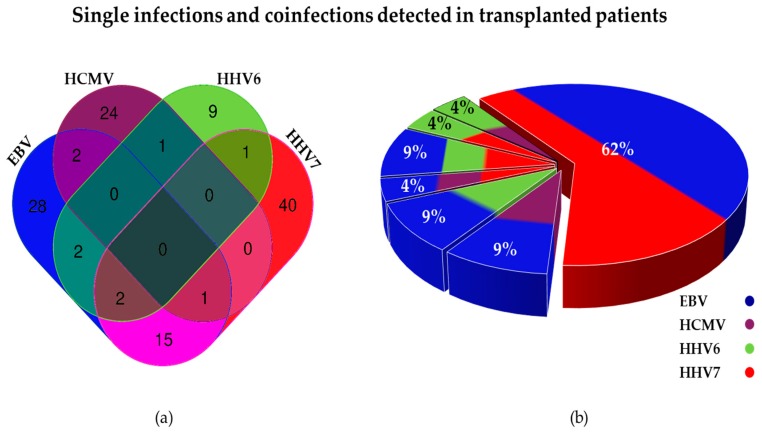
Single and multiple infections by beta and gamma herpesviruses detected in transplanted patients. (**a**) Venn diagram showing number of positive samples for each herpesvirus. Number of single infections can be seen at the ends of the diagram, while coinfections can be seen at overlapping areas (*N* = 125). (**b**) Pie diagram showing proportions of multiple infections (*N* = 24).

A total of 24 samples (from 10 patients) showed positive viral loads to more than one herpesvirus: 21 were double infections and 3 were triple infections. The most frequent coinfection was double infection with EBV and HHV7 (62%), followed by double infection with EBV and HHV6 (9%), and triple infection with EBV, HHV6 and HHV7 (9%) (Figure 5). Quadruple coinfections were never detected. The first PCR products found positive to viruses in single infection or coinfection were Sanger-sequenced to confirm their viral identity.

#### 3.4.2. Comparison between Viral Loads Detected in Leukocytes vs. Plasma

**Figure 6:**
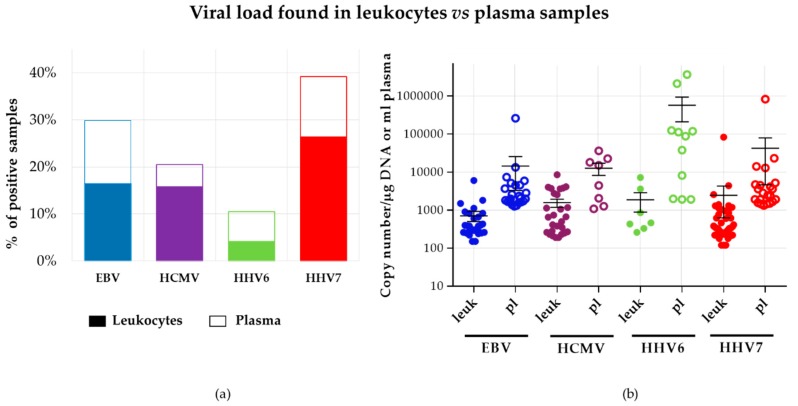
Comparison between viral loads found in leukocytes vs. plasma samples. Panel (**a**) depicts percentages of positive samples. Virus positive in leukocytes or plasma is represented in closed or open bars, respectively. Panel (**b**) depicts the number of viral copies. Leuk: leukocytes, pl: plasma.

We then analyzed the distribution of infection positive samples in the two blood fractions. Of all positive viral detections (*N* = 171), 64 (37.6%) were detected in plasma and 107 (62.4%) were detected in leukocytes. Figure 6a shows frequency of positive samples per each virus in both blood fractions. Overall, the distribution range of viral loads in this study was wide, from 120 to 82,470 copies/μg of DNA in leukocytes and from 1080 to 3,675,240 copies/mL in plasma. Of note, the majority of positive samples were concentrated in a single log with similar medians within its blood fraction. Thus, positive samples found in leukocytes were around 10^2^–10^3^ copies/µg while, for plasma, they were located one log higher: 10^3^–10^4^ copies/mL, with the exception of HHV6 that was two logs higher in plasma. HHV6 and HHV7 were the viruses with the highest load in plasma and leukocytes, respectively (Figure 6b).

**Figure 7:**
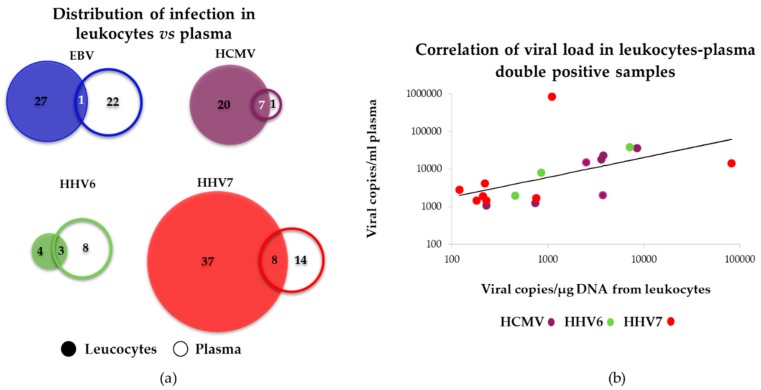
Comparison between viral loads found in leukocytes vs. plasma blood fractions. (**a**) Venn diagrams showing the distribution of the different viruses. For each virus the filled circle indicates the number of positive samples detected in leukocytes, while the empty circle indicates the number of positive samples detected in plasma. Circles are scaled according to the number of positive samples. (**b**) Correlation of viral loads in leukocytes and plasma for samples with viruses detected in both fractions. Coefficient of correlation (R) were the following: HCMV (*N* = 7): R = 0.8648, *p* = 0.012; HHV6 (*N* = 3): R = 0.994, *p* = 0002; HHV7 (*N* = 7): R = 0.8192, *p* = 0.0495. For HHV7 one sample did not show correlation and was not considered for the analysis. EBV was not estimated.

Interestingly, 85% of positive samples were detected exclusively in one of the two blood fractions analyzed. Figure 7a shows the distribution of positive samples in leukocytes and plasma for each virus. The detection of viruses exclusively in plasma indicated that non-blood cells were the sources of viral DNA. These cells could include oropharyngeal epithelial cells and nerve cells, which are targets of herpesvirus infection. Only in 19 samples was a particular virus detected in both blood fractions; in those double positives, with the exception of one HHV7 sample, a high correlation was observed in the viral loads detected in leukocytes and plasma (Figure 7b). EBV, HCMV and HHV7 were more often found in leukocytes, while HHV6 was more often found in plasma.

#### 3.4.3. Persistence of Infections

**Figure 8:**
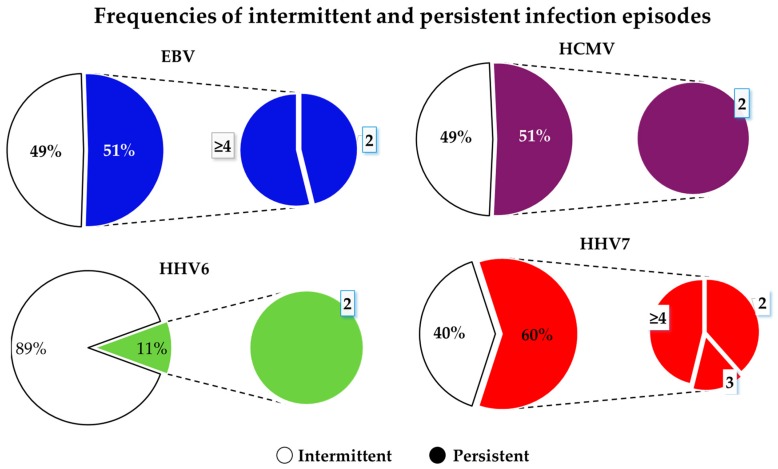
Plots showing frequency of intermittent and persistent infections. Large pie charts (**left**) show percentages of intermittent and persistent infections for each virus as indicated. Small pie charts (**right**) show proportions of 2, 3, and ≥4 sustained episodes of consecutive positive detection.

Infection episodes were analyzed according to the bi-weekly/monthly follow-up. Intermittent episodes were those positive detections that were not consecutive in time. Persistent episodes were those positive in ≥2 consecutive samples. Fifty percent of all infection episodes were persistent and 16 (47%) patients presented persistent episodes. HHV7 showed the most stable infection with 60% of positive episodes exhibiting sustained detection. Fifty-one percent of EBV and HCMV positive episodes were persistent, while only 11% for HHV6. HHV7 and EBV showed the longest-lasting periods of sustained detectable infection (Figure 8).

#### 3.4.4. Association between Viral Infection and Episodes of Graft Rejection

Among the most severe complications after transplantation is rejection of the transplanted organ and development of lymphoproliferative disease (LPD). Of 34 patients analyzed, none developed LPD, but 10 (29%) had episodes of graft rejection, with 82% of rejection episodes coinciding with an episode of viremia. Relative risk was determined as an index of association between episodes of graft rejection and positivity to viral infection. In kidney transplantation, we observed that single infection with HHV6 and double coinfection with EBV and HHV7 increased the risk of graft rejection almost six-fold. The highest observed rejection risk in renal recipients was due to the EBV, HHV6 and HHV7 triple coinfection being 17.6-fold. In hepatic recipients, only HCMV increased risk of rejection, but it was up to 40.3-fold (Table 5).

## 4. Discussion

Multiplex PCRs have allowed the identification of pathogens in many clinical and epidemiological settings [25]. Most multiplex techniques developed to date that simultaneously detect three to six beta and gamma herpesviruses are qualitative techniques that use nested strategies [26,27,28,29,30]. These approaches increase the probability of contamination and false positives due to major sample manipulation [25]. Other multiplex qPCRs only detect two to three herpesviruses leaving out important agents such as HHV7 and KSHV [16,17]. Also, previous strategies did not include a control for DNA quality. To our knowledge, the methodology developed here is the first that includes quantitative detection of EBV, HCMV, HHV6, HHV7 and KSHV, and that simultaneously evaluates the β-actin cellular gene. This multiplex qPCR showed high efficiency and predictability, and was as sensitive and specific as simplex reactions within a dynamic range of at least six orders of magnitude, which is the coverage recommended [31]. In addition to post-transplant patients, we have used this technique to provide clinical advice for patients with: primary and acquired immunodeficiencies, auto-immune diseases, LPDs/lymphoma, encephalitis, Lyme disease, hemophagocytic syndromes and pediatric idiopathic hepatitis. We have also used fresh or paraffin-embedded tissue and cerebrospinal fluid with equally positive results.

We made several important observations in the cohort of pharmacologically immunosuppressed pediatric patients due to liver and kidney transplantation. For instance, HHV6 loads were the highest among the tested herpesviruses, while HHV7 was the most common virus detected in both single and coinfections. HHV6 and HHV7 are generally never monitored in post-transplant patients, and infection by these viruses has been associated with several diseases going from mild fever and rash to more severe pneumonitis and encephalitis, which could upset the well-being of the transplanted patient. Since we cannot differentiate between HHV6A and HHV6B, it would be interesting in future studies to assess which of these viruses is the one responsible for these high loads. Also, we did not detect KSHV positive patients. There are not epidemiological studies addressing the prevalence of KSHV in Mexico, and KSHV serology is not tested in our transplant centers nor in blood banks. It is possible that KSHV infection becomes more clinically relevant in adults with an immunocompromised system.

Beta and gamma herpesviruses are characterized by a biphasic life cycle, with a latent cycle mainly in hematopoietic cells and a lytic cycle mainly in tissues, such as the oropharyngeal epithelial cells in which it serves to disseminate the virus to new hosts. Monitoring of these viruses in clinical practice is usually done in whole blood or plasma [32], since leukocyte purification is a time- and budget-consuming step. We thought that leukocytes and plasma could better hint of unchecked events of latent and lytic infection, respectively. We observed that viral loads were 10–100-fold higher in plasma than in leukocytes, which may be explained by the high number of viral genomes produced during active replication. Still, viral loads detected in plasma and leukocytes may not be comparable since copy numbers are reported in different units (µg of DNA vs. mL of plasma). More interestingly, we observed that the viruses tended to be present in leukocytes or plasma in an almost mutually exclusive manner, with all viruses appearing to behave dissimilarly. In this cohort of patients, HCMV and HHV7 were surprisingly more frequently elevated in leukocytes, probably suggesting states of unregulated latency. Elevated HHV6 was more often found in plasma, suggesting an unregulated lytic phase, while high copy numbers of EBV were evenly distributed in both blood fractions. We were surprised to find viruses in plasma without a concomitant rise of viruses in the cellular fraction. These episodes may result from lytic infection in non-blood tissues. Unchecked lytic phases are associated with local tissue damage [33], while EBV unchecked latent phase is associated with LPDs and sarcomas [34]. An unregulated lytic cycle has another important clinical implication, since it can be successfully treated with acyclovir-related drugs that specifically impair the activity of viral DNA polymerases. We did not observe LPDs in this cohort of patients, but expansion of infected cells is associated with latent infection and it is dependent on cellular polymerases that are not affected by this kind of drugs.

The simultaneous monitoring of beta and gamma herpesviruses also allowed us to look for potential evidence of viral cooperation. We observed numerous events of multiple infection, and EBV/HHV7 was the most common coinfection observed. However, the viruses were most frequently present in single infection rather than in coinfection episodes (97 out of 125 events) and the most robust numbers were also observed in single infection (although without statistically significant differences). In spite of not observing differences between viral loads in single or multiple infection episodes, we found evidence that coinfections significantly increase the risk of organ rejection. Particularly, EBV and HHV7 together were associated with a 5.6-fold increased risk of events of kidney rejection. Interestingly, single infection with HHV6 also increased the risk of rejection of the transplanted kidney by 5.6-fold, but, together with EBV and HHV7, increased rejection risk up to almost 18 times. These rejection episodes correlating with coinfection may hint to potential mechanisms of viral increased pathogenicity. HCMV was the only virus associated with risk of rejection of the transplanted liver. However, this was the highest number observed with 40-fold increased risk. HCMV single infection was also nearly significantly associated with risk of kidney rejection. Because we almost did not observe episodes of HCMV coinfection with the other herpesviruses we do not know whether HCMV coinfections further aggravate the fate of the transplanted organ. Several previous studies support the notion that HCMV increases the risk of graft rejection [2,35,36]. On the contrary, single infection with EBV or HHV7 does not seem to increase the risk of graft rejection. We believe that this study supports the idea that HHV6 and HHV7 should be included in routine follow-up tests of transplanted patients and perhaps in other immunocompromised individuals. It is also important to consider that EBV and HCMV are continuously monitored and the physicians overseeing patients are dynamically modifying the dosage of immunosuppressive drugs in response to the viral titers observed. Because of this, it is possible that we were restricted or biased to events of increased pathogenicity triggered by HHV6 and HHV7. Since cooperative coinfections have been described between EBV and HIV, *Plasmodium falciparum*, *Helicobacter pylori* and other herpesviruses [3,4,5,6,7], in future studies, it will be informative to assess mechanisms of viral cooperation between beta and gamma herpesviruses in the context of immunosuppression.

Because only EBV and HCMV are tested during transplantation, we only know the pre-transplant serologic status for these viruses in some of the patients, while we ignore the serological status of HHV6 and HHV7. However, according to the data that we have available, there was a high frequency of positive serology for recipients: EBV: 92% and 82% (for kidney and liver, respectively), and HCMV: 75% and 67% (for kidney and liver, respectively). On the other hand, viral DNA was not detected on day 0 for most of the patients (pre-transplant sample), except for 4/34 patients. Three of the positive patients presented single positive infection with EBV (plasma), HCMV (leukocytes) and HHV7 (leukocytes), respectively, while one patient presented coinfection with EBV and HHV7 (both viruses in leukocytes). Interestingly, two of the four patients who had positive viral loads at time 0 also presented graft rejection. Because most patients are already seropositive at transplantation, most probably these picks of viral loads were due to the immunosuppression associated with the undernourishment that is frequently observed in patients with end-organ disease. We believe that these data highlight the importance of monitoring viral loads of beta and gamma herpesviruses rather than only EBV and HCMV serology in patients undergoing transplantation.

We also analyzed whether rejection was associated with the presence of viruses in a particular blood fraction. In three out of four rejection events correlating with HCMV positivity, the virus was present in both leukocytes and plasma, which prevented those events from being included in the analysis. For the rest of the rejection events, we did not find significant associations with any blood fraction either with single or coinfection. We originally thought that rejection was more likely to be associated with viral loads in plasma, reflecting local lytic cycle and tissue inflammation contributing to rejection. However, it is likely that uncontrolled latent infection in immunosuppressed patients may also lead to the emergence of a strong immune response that contributes to graft rejection. We did not characterize the cellular and viral inflammatory factors that were present during the episodes of infection; in later studies, it would be mandatory to explore this idea. The abortive lytic cycle described for EBV, HCMV and KSHV is defined as the initiation of the lytic phase with the consequent expression of some immunomodulatory lytic genes without amplification of the viral genome nor the formation and budding of viral particles from the host cells [37]. However, this abortive cycle is characterized by the expression of a large number of viral cytokines/chemokines and of a counteracting inflammatory response. It may be possible that in leukocyte-restricted high viral loads, the abortive lytic cycle-induced inflammation is also cooperating with graft rejection.

## Figures and Tables

**Table 1:** Characteristics of primers and probes used in two-tube multiplex qPCR for detection of beta and gamma herpesviruses.

Tube ^1^	Viral/Cellular Target	Gene	F-Q	Sequence 5′→3′	Amplicon Size (bp)	Ref.
1	EBV	BALF5	FAM-BHQ1a	**F** CCCTGTTTATCCGATGGAATG **R** CGGAAGCCCTCTGGACTTC **P** TGTACACGCACGAGAAATGCGCC	90	[19]
	HCMV	UL123	JOE-BHQ1a	**F** GCTACAATAGCCTCTTCCTCATCTG **R** GACTAGTGTGATGCTGGCCAAG **P** AGCCTGAGGTTATCAGTGTAATGAAGCGCC	201	[20]
	Endogenous human gene	β-actin	Cy5-BHQ3a	**F** CCAGGCTAACCTCGGAAATCT **R** CATCGTCATTCCTGTGCAACT **P** TGGGGTGCCGGCTCTCTGCT	225	This study
2	HHV6	U31	FAM-BHQ1a	**F** CGACTCTCACCCTACTGAACGA **R** GAGGCTGGCGTCGTAGTAGAA **P** AGCCACAGCAGCCATCTACATCTGTCAA	121	[20] and this study ^2^
	HHV7	U57	JOE-BHQ1a	**F** CGGAAGTCACTGGAGTAATGACAA **R** ATGCTTTAAACATCCTTTCTTTCGG **P** CTCGCAGATTGCTTGTTGGCCATG	108	[21]
	KSHV	LANA	Cy5-BHQ3a	**F** AGTTATGGGCGACTGGTCTG **R** GGATGGAAGACGAGATCCAA **P** AAGTCCGTATGGGTCATTGC	166	This study

F-Q: Fluorophore-Quencher, FAM: 6-Carboxyfluorescein, JOE: 6-Carboxi-4′,5′-Dichloro-2′,7′-Dimethoxyfluorescein, succinimidyl ester, Cy5: Cyanine 5, BHQ: Black Hole Quencher, F: Forward, R: Reverse, P: Probe. ^1^ Viral detection was carried out through a two-tube multiplex qPCR; Epstein-Barr Virus (EBV), human cytomegalovirus (HCMV) and β-actin were detected in one tube (named tube 1), while human herpesvirus (HHV) 6, HHV7 and Kaposi Sarcoma Herpesvirus (KSHV) were detected in a separate tube (named tube 2). ^2^ Forward and reverse primers were de novo designed for this study while the probe sequence was previously described [20].

**Table 2:** Efficiency, predictability and limit of detection of the multiplex standard curves and correlation of single vs. multiplex qPCR.

Target	E (%)	Predictability (R^2^)	LOD (Copy Number)	Correlation of Single vs. Multiplex qPCR (R)
EBV	94	0.993	21	0.9994
HCMV	92	0.988	18	0.9904
HHV6	96	0.988	25	0.9998
HHV7	95	0.993	21	0.9981
KSHV	95	0.996	18	0.9999

E: Efficiency, R^2^: Coefficient of determination (correlation between standard plasmid copies and cycle thresholds (also in Figure 2a)), LOD: Limit of detection, R: Coefficient of correlation (correlation of copy number in single vs. multiplex qPCR (also in Figure 2b)).

**Table 3:** Precision of multiplex qPCR.

Standard (Plasmid Copies)	Coefficients of Variation (%)									
	EBV		HCMV		HHV6		HHV7		KSHV	
	Intra ^1^	Inter ^2^	Intra ^1^	Inter ^2^	Intra ^1^	Inter ^2^	Intra ^1^	Inter ^2^	Intra ^1^	Inter ^2^
10^6^	0.31	0.96	0.73	3.12	0.44	1.89	0.54	0.49	0.31	0.66
10^5^	0.77	1.77	2.93	2.89	0.91	1.21	0.44	0.76	0.31	0.96
10^4^	0.96	1.49	1.64	2.94	0.93	1.33	0.69	0.91	1.27	1.13
10^3^	1.43	1.75	3.30	2.35	0.50	1.95	1.05	0.85	0.67	0.57
10^2^	1.82	1.88	1.65	2.53	0.43	1.63	0.87	0.69	0.69	1.09
10^1^	2.54	2.09	3.55	3.87	0.65	0.72	1.43	2.67	1.19	2.60

^1^ Refers to intra-assay repeatability. ^2^ Refers to inter-assay reproducibility.

**Table 4:** Demographic and clinical characteristics of the patients (*N* = 34).

Transplanted Organ	Kidney *N* = 22	Liver *N* = 12
**Age at transplant**		
Median of years (range)	15 (6–17)	6 (4–17)
**Gender**		
Female	33%	58%
Male	67%	42%
**Kind of donor**		
Cadaveric	57%	92%
Living-donor	43%	8%
**EBV serostatus N (%) ^1^**		
Donor+ Recipient+	11 (92%)	6 (55%)
Donor+ Recipient−	1 (8%)	2 (18%)
Donor− Recipient+	0 (0%)	3 (27%)
Donor− Recipient−	0 (0%)	0 (0%)
**HCMV serostatus N (%) ^1^**		
Donor+ Recipient+	13 (62%)	6 (50%)
Donor+ Recipient−	3 (19%)	3 (25%)
Donor− Recipient+	2 (13%)	2 (17%)
Donor− Recipient−	1 (6%)	1 (8%)
**Pre-transplant diagnosis** **N (%)**	ESRD of unknown etiology 15 (68%) Focal and segmental glomerulosclerosis 2 (9%) ESRD secondary to complex uropathy 2 (9%) ESRD secondary to hypoplasia JRA 1 (5%) Microscopic polyangiitis 1 (5%) Posterior urethral valves 1 (5%)	Biliary atresia 3 (25%)
		Fulminant hepatitis 2 (17%)
		Liver fibrosis 1 (8%)
		Neonatal giant cell hepatitis 1 (8%)
		Progressive intrahepatic family cholestasis 1 (8%)
		Bayler disease 1 (8%)
		Alalgille syndrome 1 (8%)
		Tyrosinemia 1 (8%)
		Cholesterol ester deposition 1 (8%)

^1^ Information of EBV and HCMV serostatus was only available from 23 and 31 pairs of donor/recipient, respectively. Percentages were calculated from these numbers. Most cases in which we lack serology were due to cases of cadaveric donors. ESRD: End-stage chronic kidney disease, JRA: Juvenile rheumatoid arthritis.

**Table 5:** Relative risk of organ rejection.

Infection/Coinfection	Kidney		Liver	
	Relative Risk	*p* Value	Relative Risk	*p* Value
EBV	2.74	0.20	2.68	0.41
HCMV	4.28	0.06	**40.33**	**0.0013**
HHV6	**5.60**	**0.03**	6	0.20
HHV7	3.04	0.12	1.27	0.87
EBV/HCMV	4.86	0.24	NOT DETECTED	
EBV/HHV6	4.86	0.24	9.07	0.10
EBV/HHV7	**5.60**	**0.03**	4.46	0.29
HCMV/HHV6	7.32	0.12	NOT DETECTED	
HCMV/HHV7	NOT DETECTED		NOT DETECTED	
HHV6/HHV7	4.86	0.24	NOT DETECTED	
EVB/HCMV/HHV6	NOT DETECTED		NOT DETECTED	
EBV/HCMV/HHV7	7.32	0.12	NOT DETECTED	
EBV/HHV6/HHV7	**17.64**	**0.0003**	NOT DETECTED	
HCMV/HHV6/HHV7	NOT DETECTED		NOT DETECTED	
EBV/HCMV/HHV6/HHV7	NOT DETECTED		NOT DETECTED	

Relative risks with significant *p* are in bold.

## Supplementary Materials

Supplementary materials can be found at http://www.mdpi.com/1999-4915/10/12/730/s1.
