# Supplementary material for: Simultaneous Detection of Beta and Gamma Human Herpesviruses by Multiplex qPCR Reveals Simple Infection and Coinfection Episodes Increasing Risk for Graft Rejection in Solid Organ Transplantation
Source: Viruses. 2018 Dec 19;10(12):730. doi: 10.3390/v10120730 (PMC6316002; doi:10.3390/v10120730)
Supplement: Supplementary file 1 [file viruses-10-00730-s001.zip › supplementary materials.pdf]

Supplementary information

Supplementary Figure S1. Cross-reactivity assays. The primers and fluorescent probe used to detect one virus were tested for cross-reactivity with the other viruses (as it is indicated in the top of each panel). (a) EBV: Positive fluorescent signal was only detected when EBV DNA, or serial dilutions (10^6-10^1) of the EBV standard plasmid were used as template. For all panels, the amplification cycle (Cycle) is shown in the X axis while the normalized fluorescence (Norm. Fluoro.) is shown in the Y axis. Horizontal red lines indicate the Cycle Threshold (Ct). Dotted vertical red lines indicate the limit of detection (LOD). When fluorescence crossed the Ct to the right of this dotted line, virus detection is considered negative. (b) HHV6, (c) HHV7 and (d) KSHV.

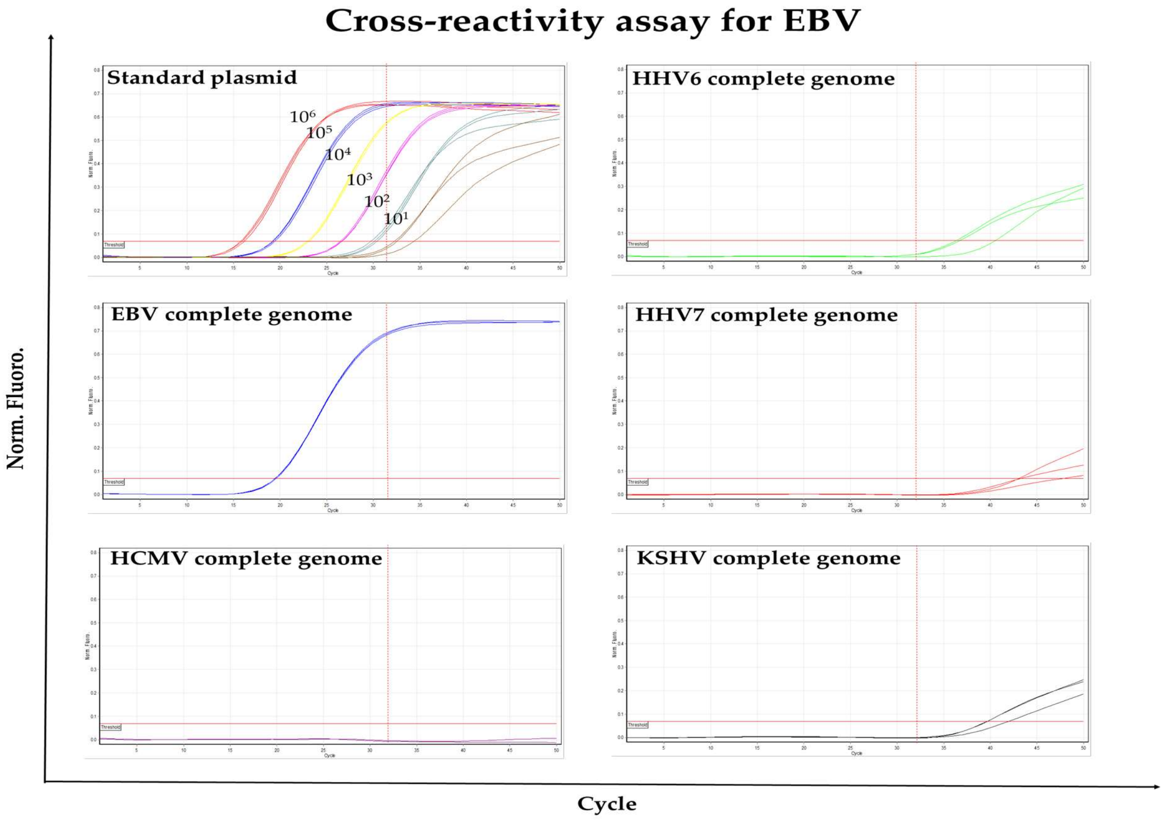

(a)

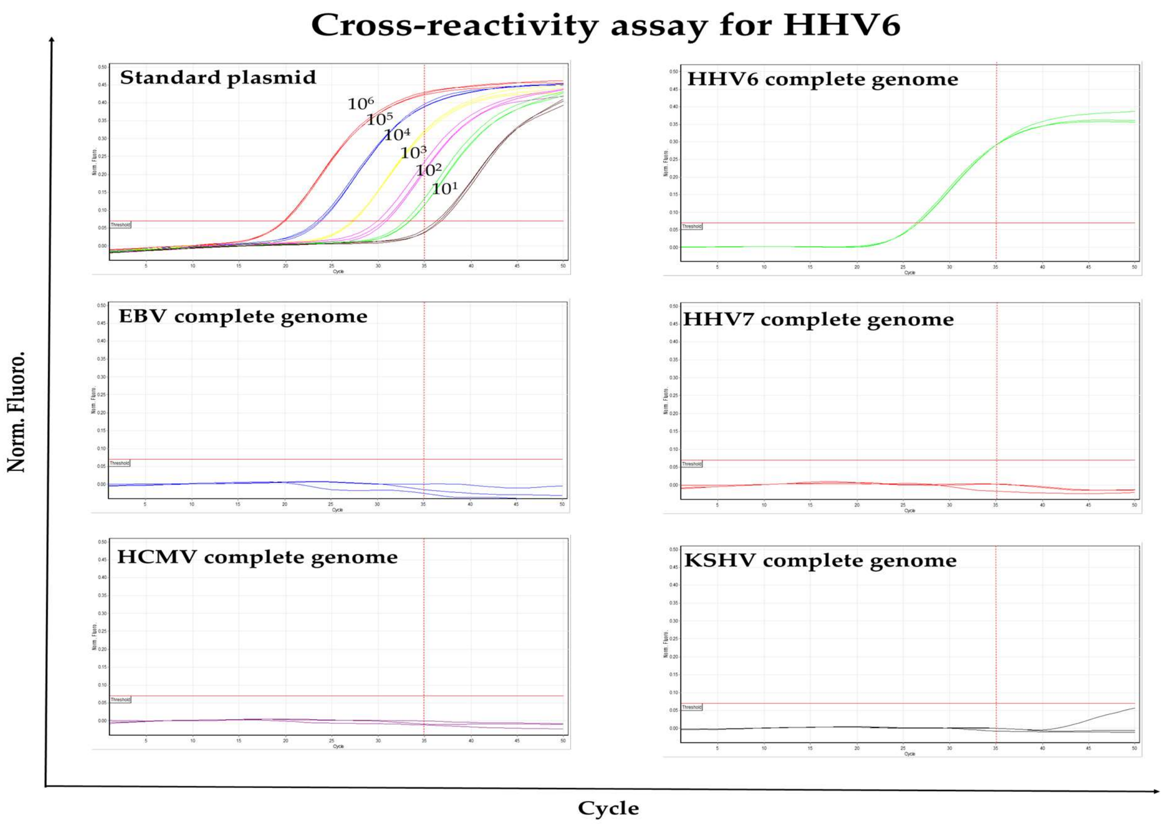
Cross-reactivity assay for HHV6
Standard plasmid
10⁶
10⁵
10⁴
10³
10²
10¹
HHV6 complete genome
EBV complete genome
HHV7 complete genome
HCMV complete genome
KSHV complete genome
Norm. Fluoro.
Cycle

(b)

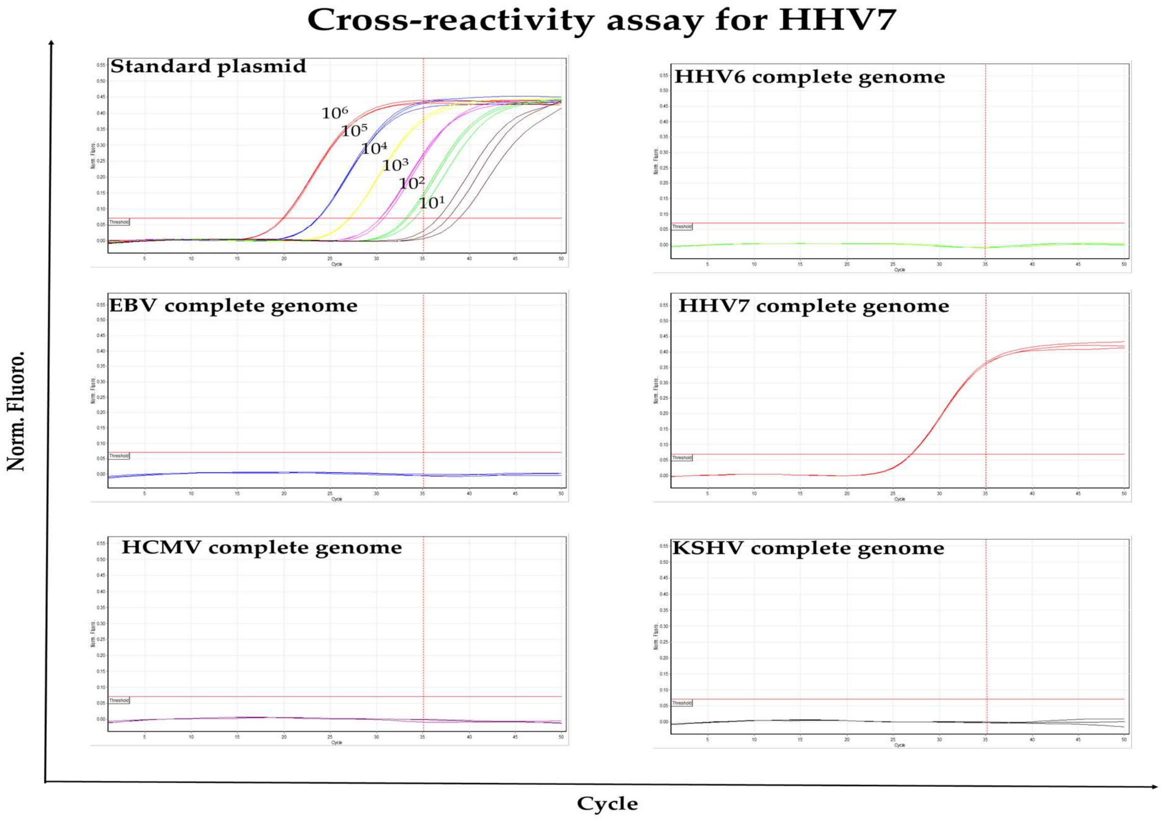
Cross-reactivity assay for HHV7
Standard plasmid
10^6
10^5
10^4
10^3
10^2
10^1
HHV6 complete genome
EBV complete genome
HHV7 complete genome
HCMV complete genome
KSHV complete genome
Norm. Fluoro.
Cycle

(c)

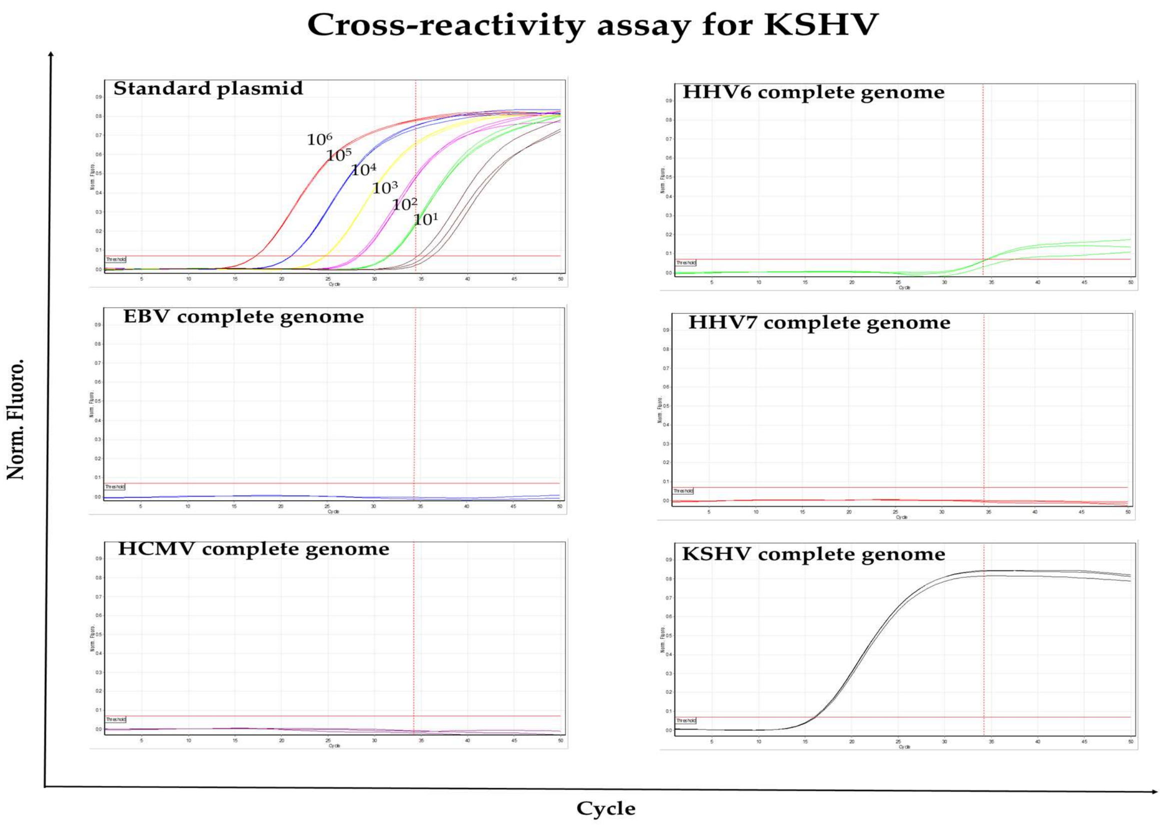

Cross-reactivity assay for KSHV
Norm. Fluoro.
Cycle
Standard plasmid
10^6
10^5
10^4
10^3
10^2
10^1
HHV6 complete genome
EBV complete genome
HHV7 complete genome
HCMV complete genome
KSHV complete genome

(d)

Supplementary Figure S2. Kinetics of infection of kidney-transplanted pediatric patients. (a)-(r): Patients 1 to 18. The follow up period is shown in the X axis. Samples of day 0 were collected on the same day of transplant just before the surgery. Viral loads determined as the copy number/µg of DNA (leukocytes) or copy number/ml (plasma) are shown in the Y axis. Viruses are color-coded as indicated. Infections detected in leukocytes (leuk) are drawn as continuous lines while those detected in plasma (pl) are drawn as dotted lines. Horizontal lines below one viral copy indicate that virus was undetected.

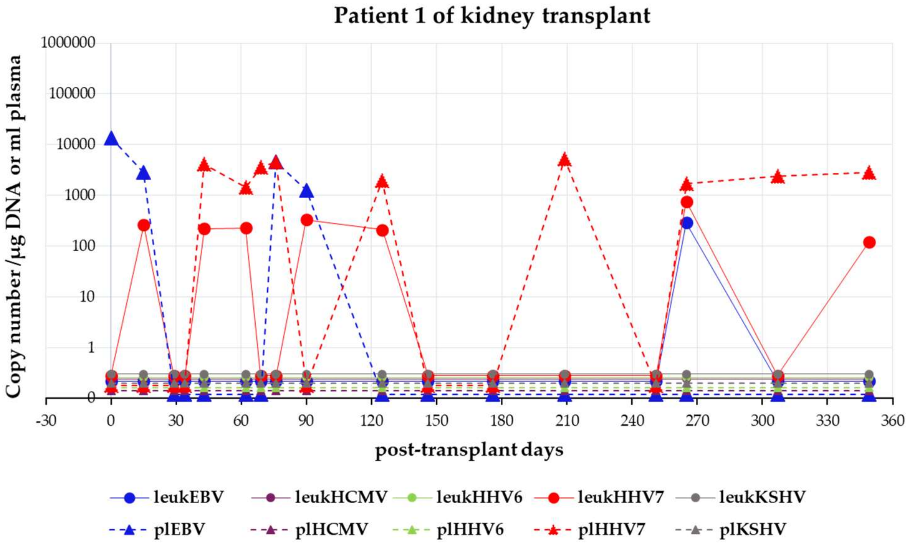

(a)

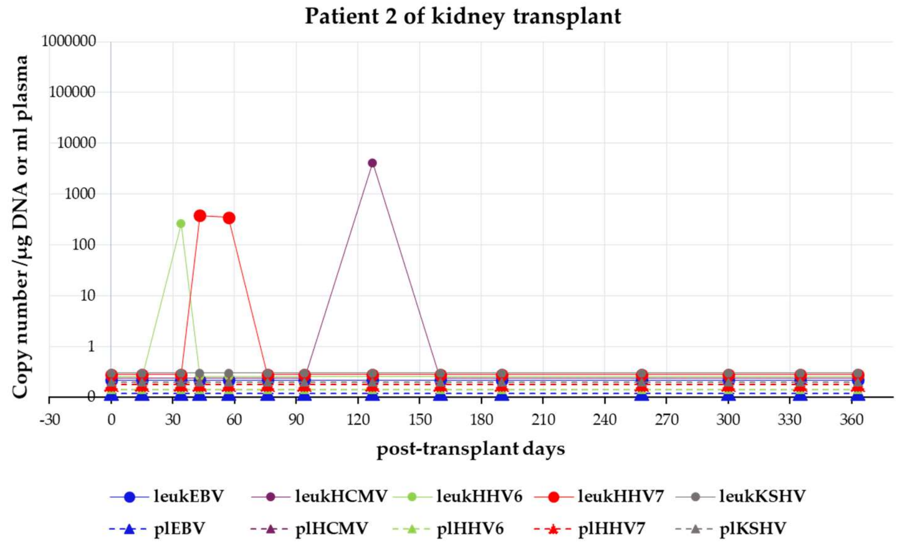

(b)

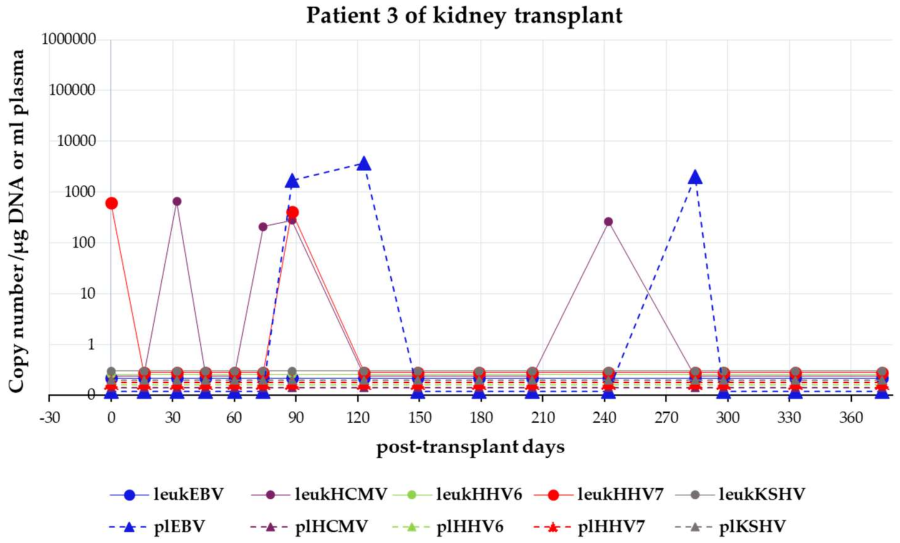
Patient 3 of kidney transplant
Copy number /µg DNA or ml plasma
1000000
100000
10000
1000
100
10
1
0
-30
0
30
60
90
120
150
180
210
240
270
300
330
360
post-transplant days
leukEBV
leukHCMV
leukHHV6
leukHHV7
leukKSHV
plEBV
plHCMV
plHHV6
plHHV7
plKSHV

(c)

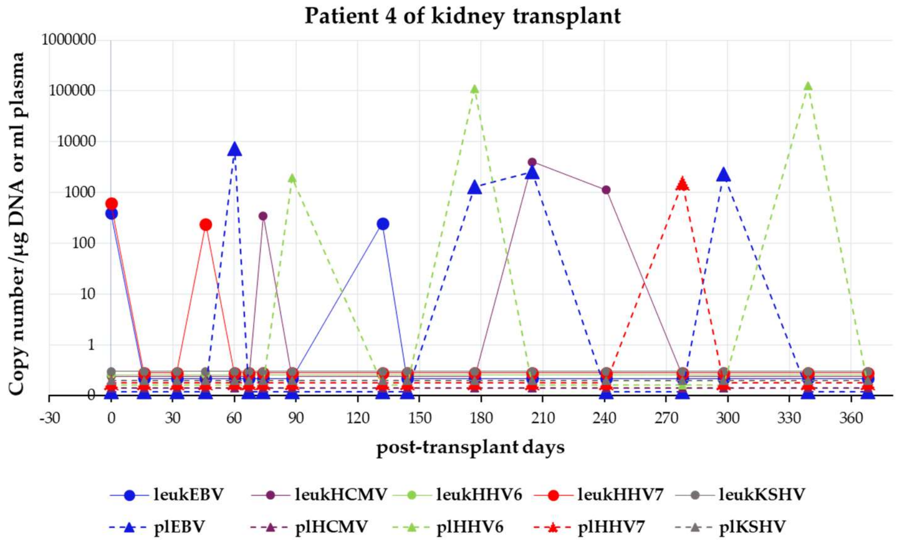
Patient 4 of kidney transplant
Copy number /µg DNA or ml plasma
1000000
100000
10000
1000
100
10
1
0
-30
0
30
60
90
120
150
180
210
240
270
300
330
360
post-transplant days
leukEBV
leukHCMV
leukHHV6
leukHHV7
leukKSHV
plEBV
plHCMV
plHHV6
plHHV7
plKSHV

(d)

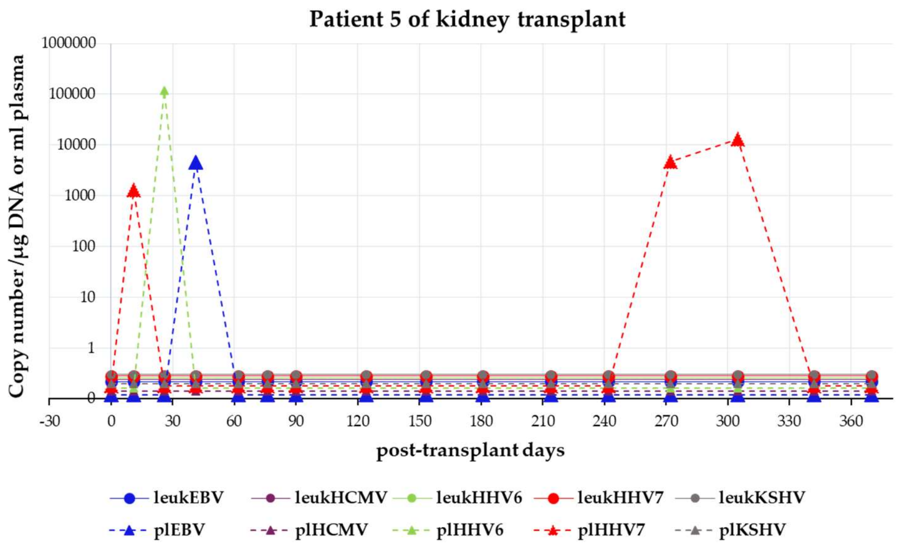
Patient 5 of kidney transplant
Copy number /µg DNA or ml plasma
1000000
100000
10000
1000
100
10
1
0
-30
0
30
60
90
120
150
180
210
240
270
300
330
360
post-transplant days
leukEBV
leukHCMV
leukHHV6
leukHHV7
leukKSHV
plEBV
plHCMV
plHHV6
plHHV7
plKSHV

(e)

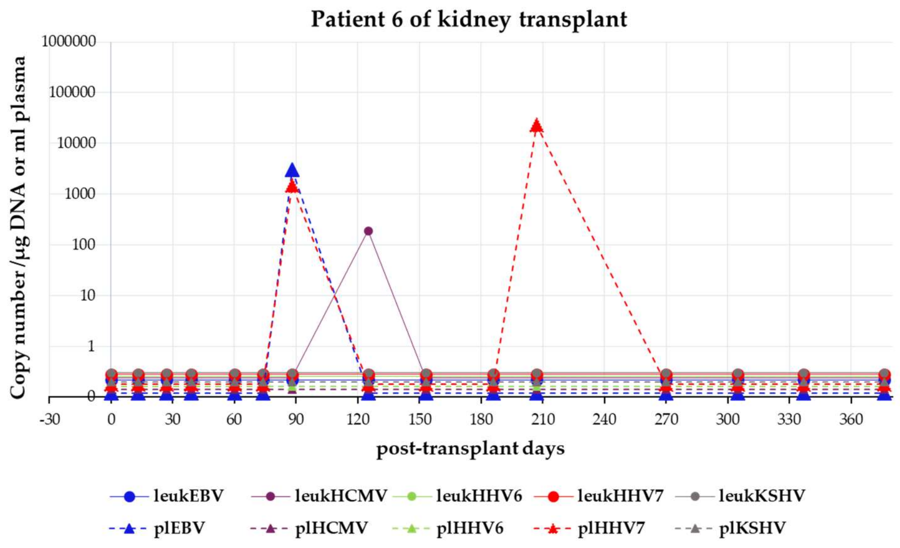
Patient 6 of kidney transplant
Copy number /µg DNA or ml plasma
1000000
100000
10000
1000
100
10
1
0
-30
0
30
60
90
120
150
180
210
240
270
300
330
360
post-transplant days
leukEBV
leukHCMV
leukHHV6
leukHHV7
leukKSHV
plEBV
plHCMV
plHHV6
plHHV7
plKSHV

(f)

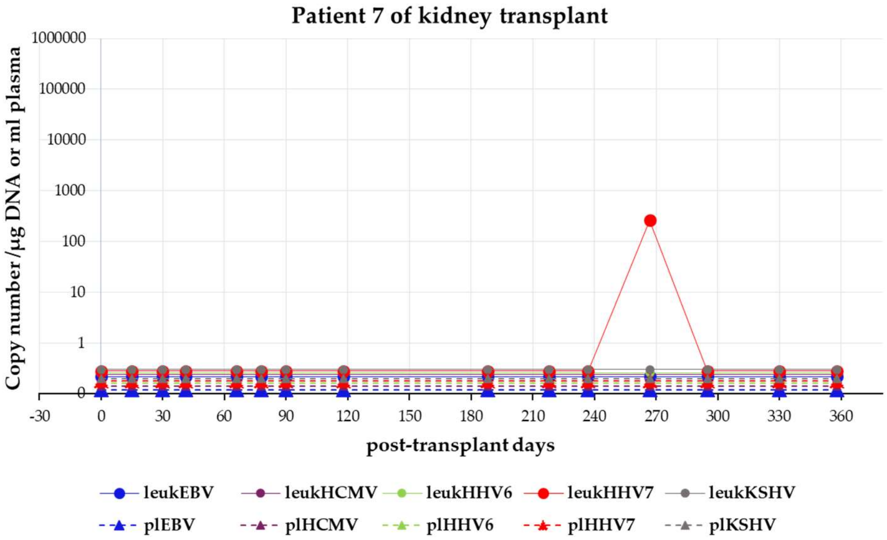
Patient 7 of kidney transplant
1000000
100000
10000
1000
100
10
1
0
-30
0
30
60
90
120
150
180
210
240
270
300
330
360
Copy number /µg DNA or ml plasma
post-transplant days
leukEBV
leukHCMV
leukHHV6
leukHHV7
leukKSHV
plEBV
plHCMV
plHHV6
plHHV7
plKSHV

(g)

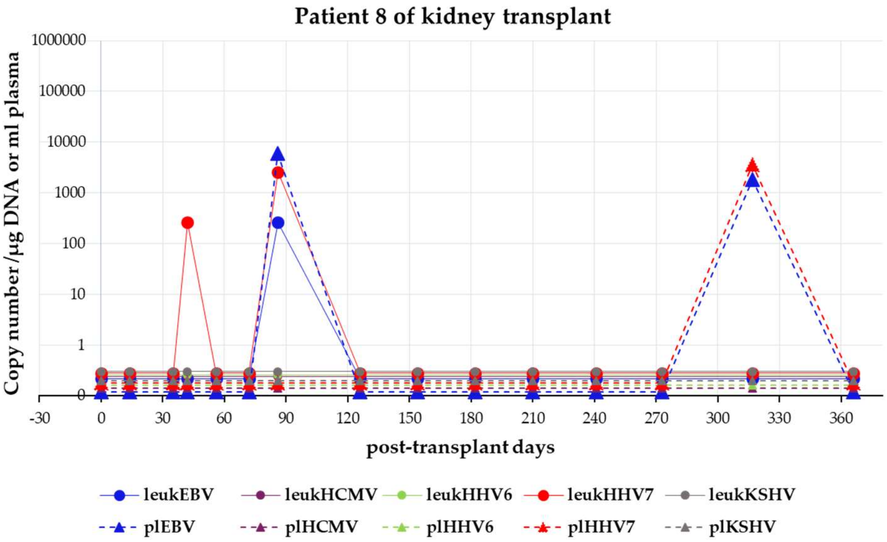
Patient 8 of kidney transplant
1000000
100000
10000
1000
100
10
1
0
-30
0
30
60
90
120
150
180
210
240
270
300
330
360
Copy number /µg DNA or ml plasma
post-transplant days
leukEBV
leukHCMV
leukHHV6
leukHHV7
leukKSHV
plEBV
plHCMV
plHHV6
plHHV7
plKSHV

(h)

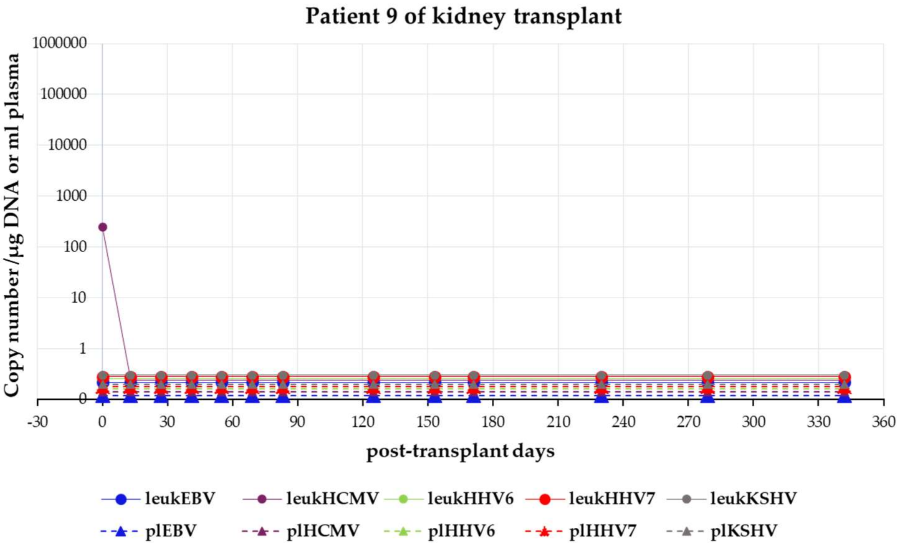
Patient 9 of kidney transplant
Copy number /µg DNA or ml plasma
1000000
100000
10000
1000
100
10
1
0
-30
0
30
60
90
120
150
180
210
240
270
300
330
360
post-transplant days
leukEBV
leukHCMV
leukHHV6
leukHHV7
leukKSHV
plEBV
plHCMV
plHHV6
plHHV7
plKSHV

(i)

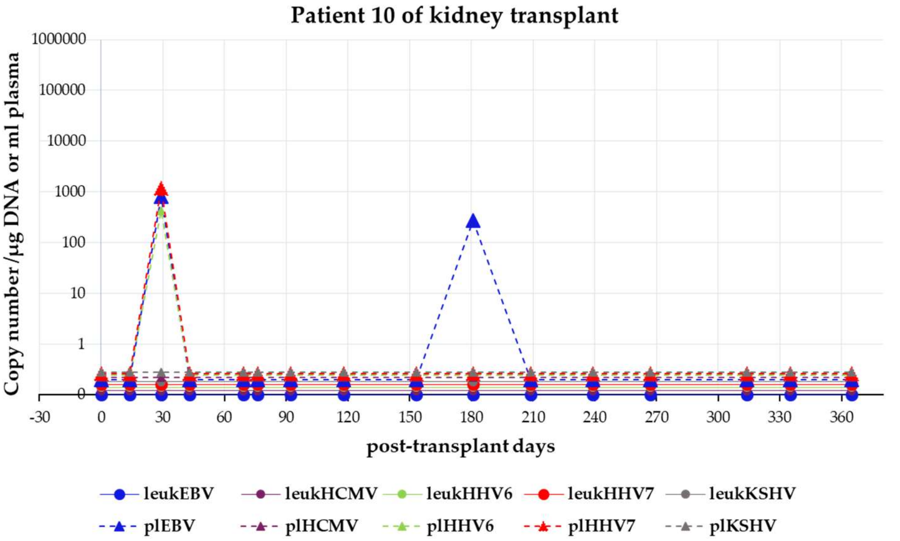
Patient 10 of kidney transplant
Copy number /µg DNA or ml plasma
1000000
100000
10000
1000
100
10
1
0
-30
0
30
60
90
120
150
180
210
240
270
300
330
360
post-transplant days
leukEBV
leukHCMV
leukHHV6
leukHHV7
leukKSHV
plEBV
plHCMV
plHHV6
plHHV7
plKSHV

(j)

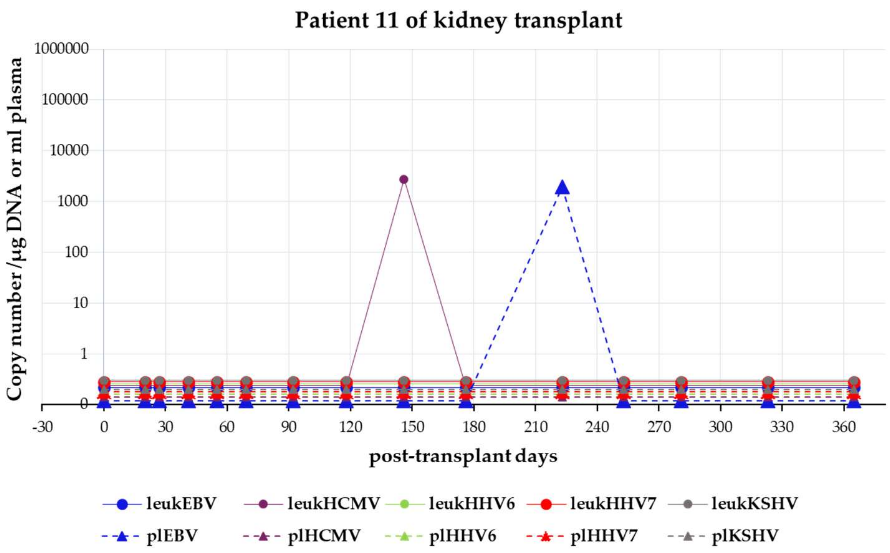

Patient 11 of kidney transplant
1000000
100000
10000
1000
100
10
1
0
Copy number/µg DNA or ml plasma
-30
0
30
60
90
120
150
180
210
240
270
300
330
360
post-transplant days
leukEBV
leukHCMV
leukHHV6
leukHHV7
leukKSHV
plEBV
plHCMV
plHHV6
plHHV7
plKSHV

(k)

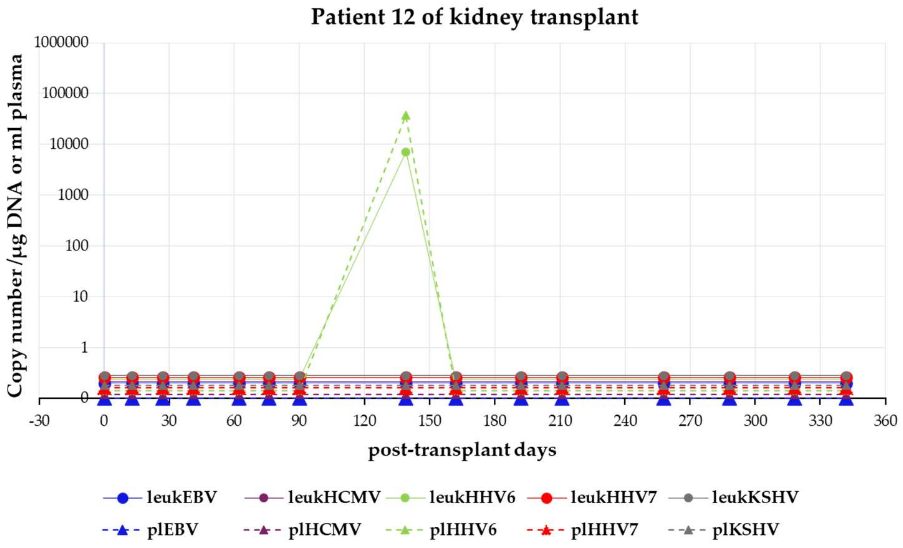

Patient 12 of kidney transplant
1000000
100000
10000
1000
100
10
1
0
Copy number/µg DNA or ml plasma
-30
0
30
60
90
120
150
180
210
240
270
300
330
360
post-transplant days
leukEBV
leukHCMV
leukHHV6
leukHHV7
leukKSHV
plEBV
plHCMV
plHHV6
plHHV7
plKSHV

(l)

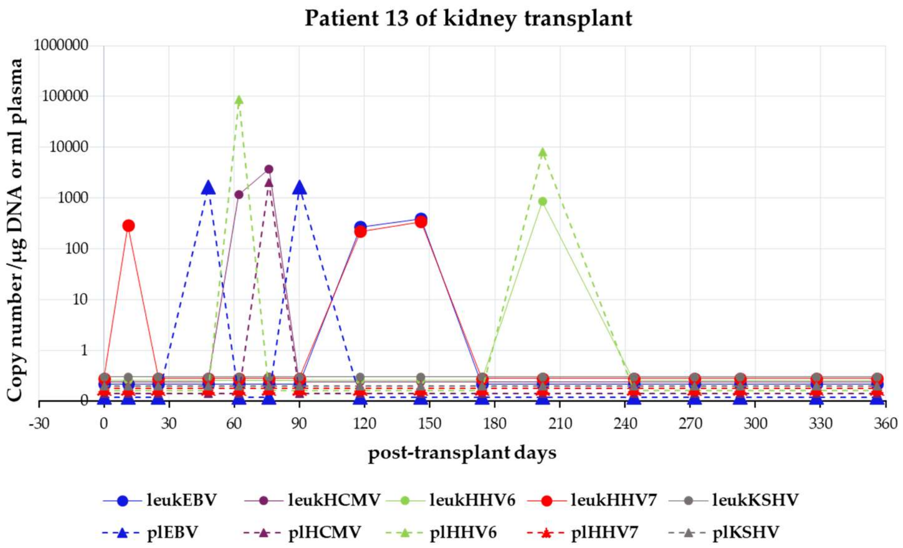
Patient 13 of kidney transplant
Copy number/µg DNA or ml plasma
1000000
100000
10000
1000
100
10
1
0
-30
0
30
60
90
120
150
180
210
240
270
300
330
360
post-transplant days
leukEBV
leukHCMV
leukHHV6
leukHHV7
leukKSHV
plEBV
plHCMV
plHHV6
plHHV7
plKSHV

(m)

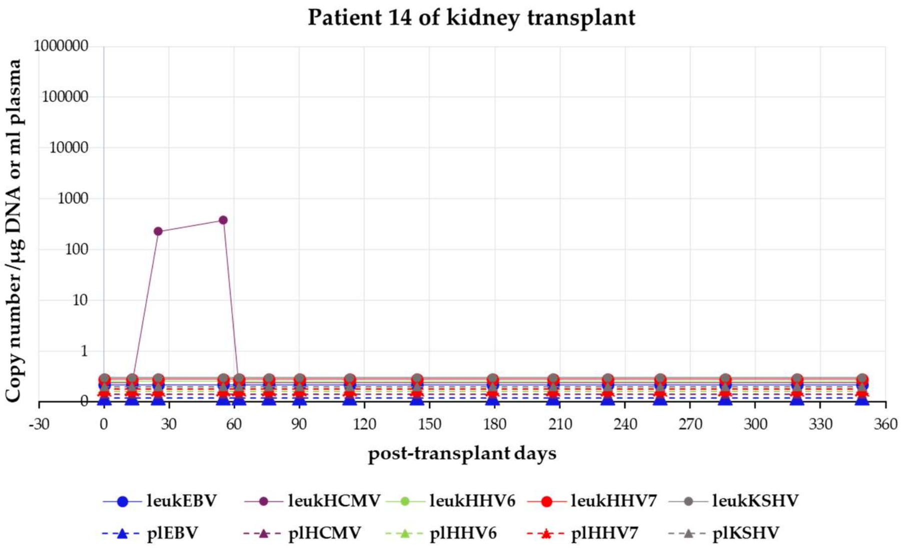
Patient 14 of kidney transplant
Copy number/µg DNA or ml plasma
1000000
100000
10000
1000
100
10
1
0
-30
0
30
60
90
120
150
180
210
240
270
300
330
360
post-transplant days
leukEBV
leukHCMV
leukHHV6
leukHHV7
leukKSHV
plEBV
plHCMV
plHHV6
plHHV7
plKSHV

(n)

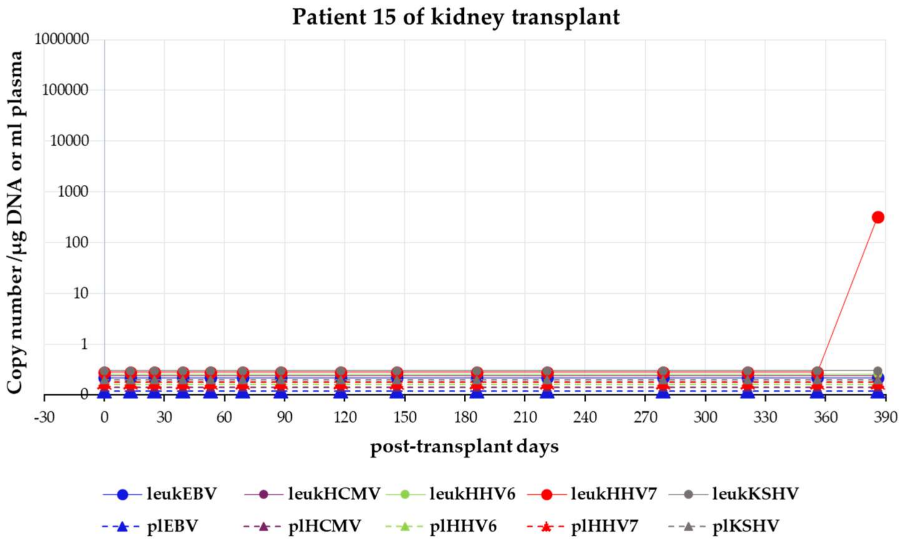
Patient 15 of kidney transplant
Copy number/µg DNA or ml plasma
1000000
100000
10000
1000
100
10
1
0
-30
0
30
60
90
120
150
180
210
240
270
300
330
360
390
post-transplant days
leukEBV
leukHCMV
leukHHV6
leukHHV7
leukKSHV
plEBV
plHCMV
plHHV6
plHHV7
plKSHV

(o)

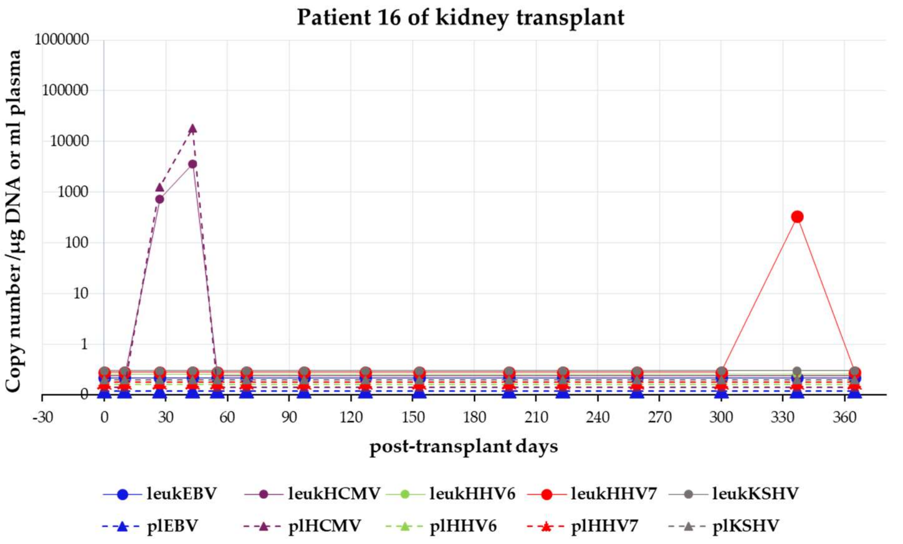
Patient 16 of kidney transplant
Copy number/µg DNA or ml plasma
1000000
100000
10000
1000
100
10
1
0
-30
0
30
60
90
120
150
180
210
240
270
300
330
360
post-transplant days
leukEBV
leukHCMV
leukHHV6
leukHHV7
leukKSHV
plEBV
plHCMV
plHHV6
plHHV7
plKSHV

(p)

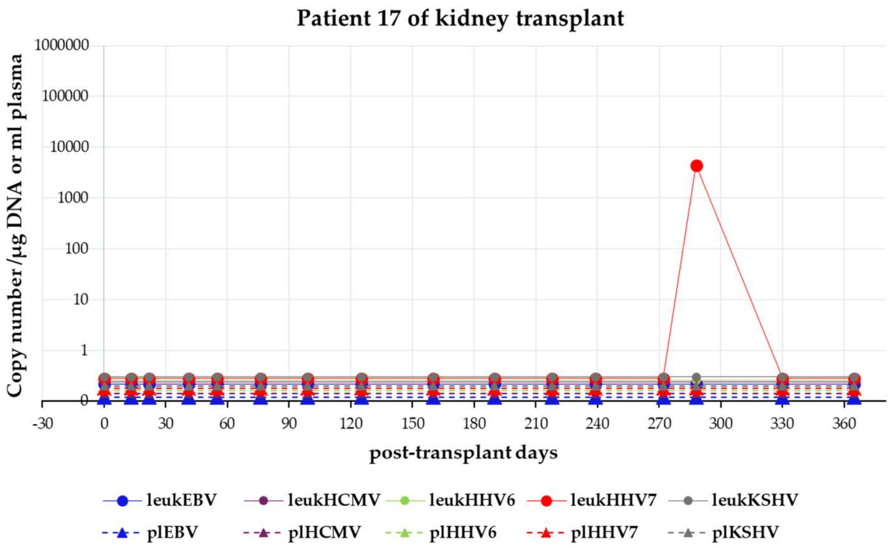
Patient 17 of kidney transplant
Copy number/µg DNA or ml plasma
1000000
100000
10000
1000
100
10
1
0
-30
0
30
60
90
120
150
180
210
240
270
300
330
360
post-transplant days
leukEBV
leukHCMV
leukHHV6
leukHHV7
leukKSHV
plEBV
plHCMV
plHHV6
plHHV7
plKSHV

(q)

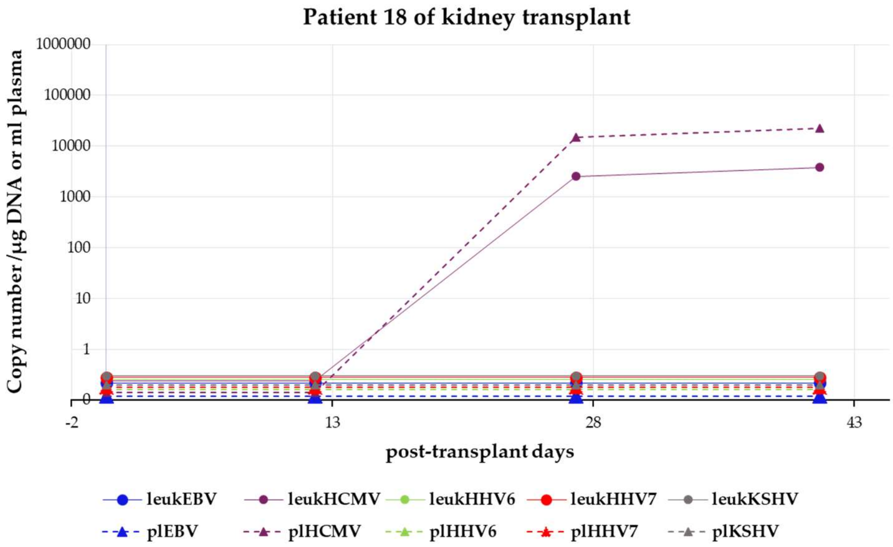
Patient 18 of kidney transplant
Copy number/µg DNA or ml plasma
1000000
100000
10000
1000
100
10
1
0
-2
13
28
43
post-transplant days
leukEBV
leukHCMV
leukHHV6
leukHHV7
leukKSHV
plEBV
plHCMV
plHHV6
plHHV7
plKSHV

(r)

Supplementary Figure S3. Kinetics of infection of liver-transplanted pediatric patients. (a)-(j): Patients 1 to 10. The follow up period is shown in the X axis. Samples of day 0 were collected on the same day of transplant just before the surgery. Viral loads determined as the copy number/μg of DNA (leukocytes) or copy number/ml (plasma) are shown in the Y axis. Viruses are color-coded as indicated. Infections detected in leukocytes (leuk) are drawn as continuous lines while those detected in plasma (pl) are drawn as dotted lines. Horizontal lines below one viral copy indicate that virus was undetected.

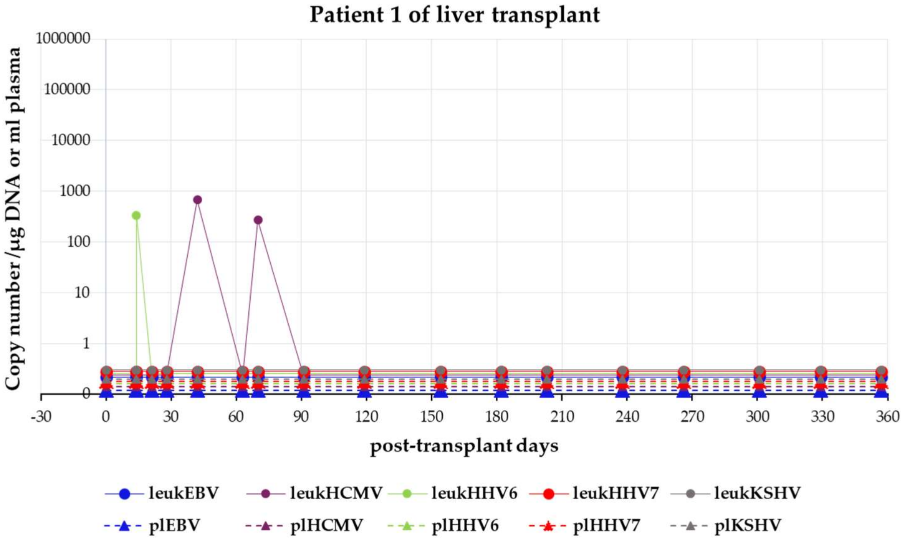

(a)

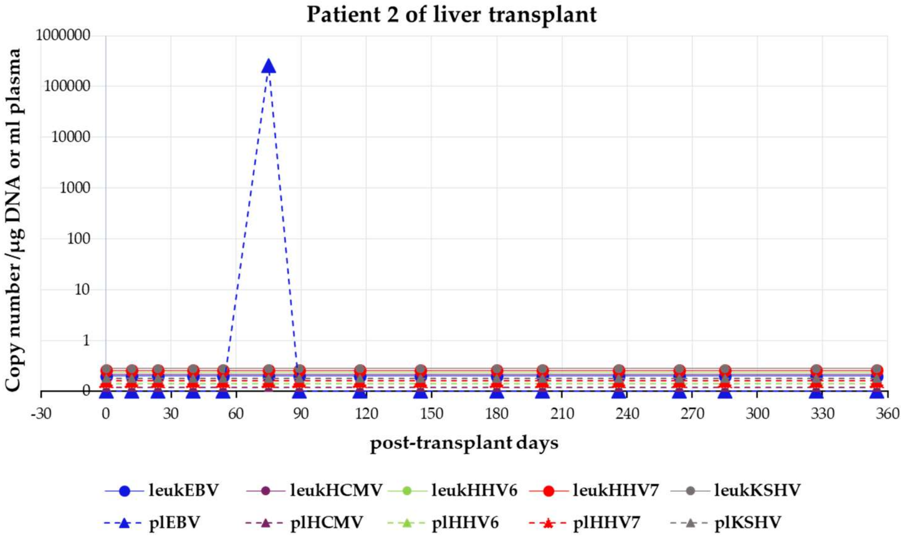

(b)

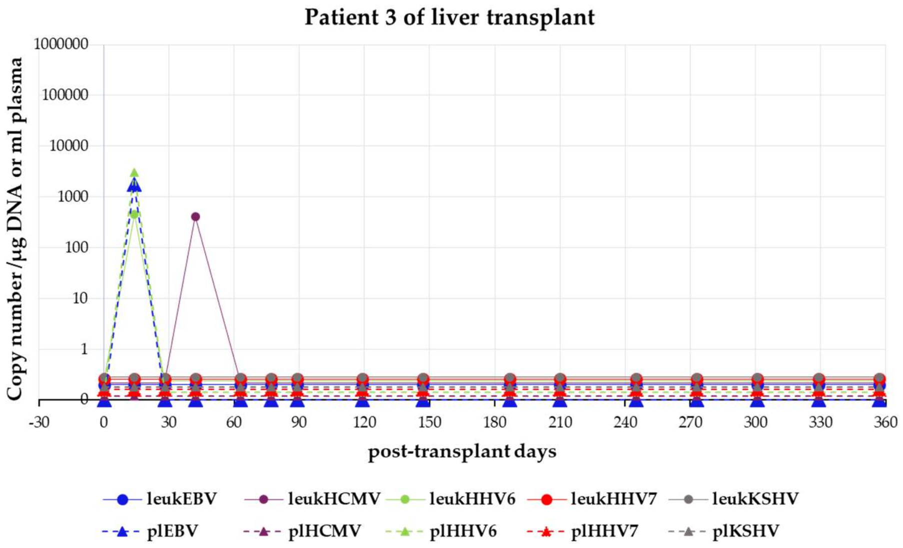
Patient 3 of liver transplant
1000000
100000
10000
1000
100
10
1
0
Copy number/µg DNA or ml plasma
-30
0
30
60
90
120
150
180
210
240
270
300
330
360
post-transplant days
leukEBV
leukHCMV
leukHHV6
leukHHV7
leukKSHV
plEBV
plHCMV
plHHV6
plHHV7
plKSHV

(c)

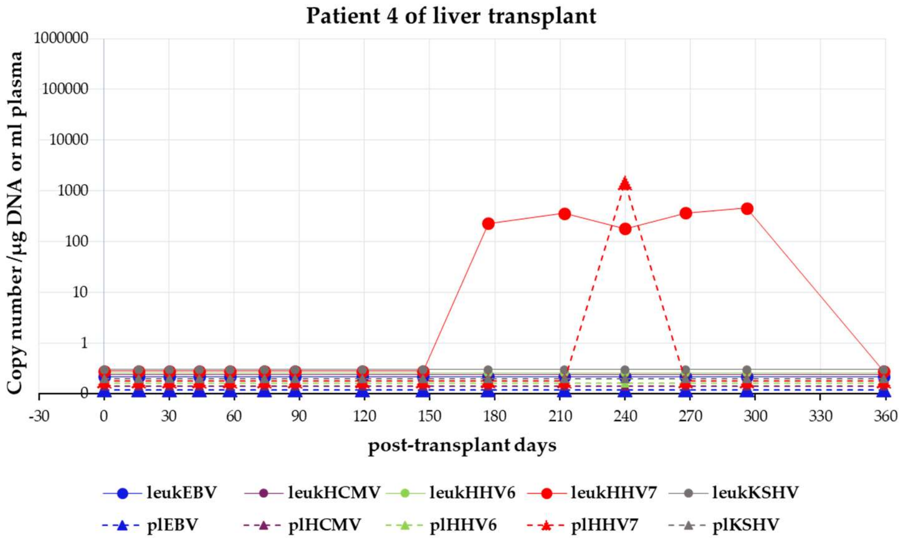
Patient 4 of liver transplant
1000000
100000
10000
1000
100
10
1
0
Copy number/µg DNA or ml plasma
-30
0
30
60
90
120
150
180
210
240
270
300
330
360
post-transplant days
leukEBV
leukHCMV
leukHHV6
leukHHV7
leukKSHV
plEBV
plHCMV
plHHV6
plHHV7
plKSHV

(d)

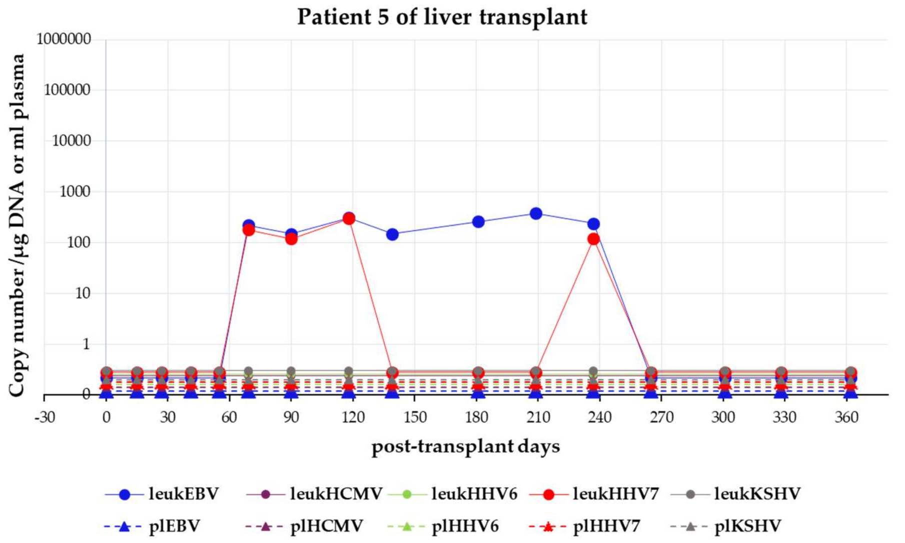
Patient 5 of liver transplant
Copy number/µg DNA or ml plasma
1000000
100000
10000
1000
100
10
1
0
-30
0
30
60
90
120
150
180
210
240
270
300
330
360
post-transplant days
leukEBV
leukHCMV
leukHHV6
leukHHV7
leukKSHV
plEBV
plHCMV
plHHV6
plHHV7
plKSHV

(e)

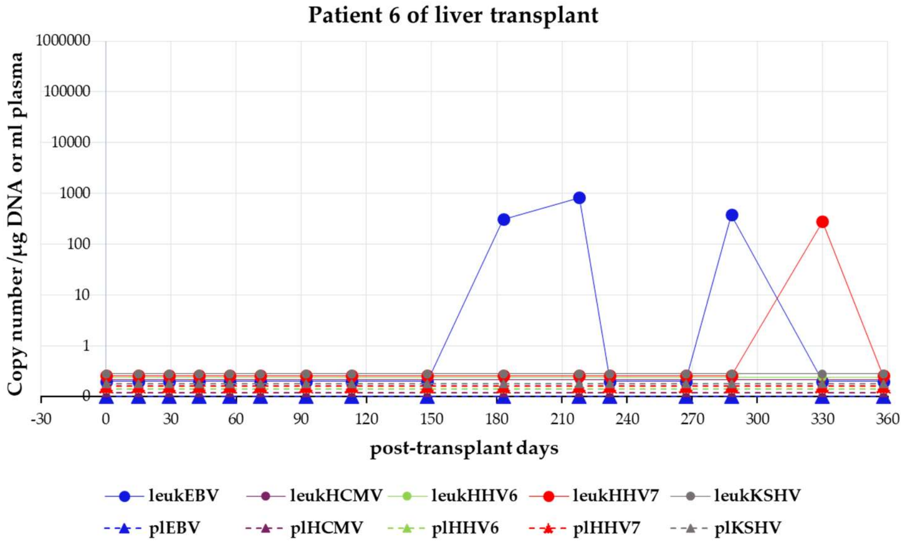
Patient 6 of liver transplant
Copy number/µg DNA or ml plasma
1000000
100000
10000
1000
100
10
1
0
-30
0
30
60
90
120
150
180
210
240
270
300
330
360
post-transplant days
leukEBV
leukHCMV
leukHHV6
leukHHV7
leukKSHV
plEBV
plHCMV
plHHV6
plHHV7
plKSHV

(f)

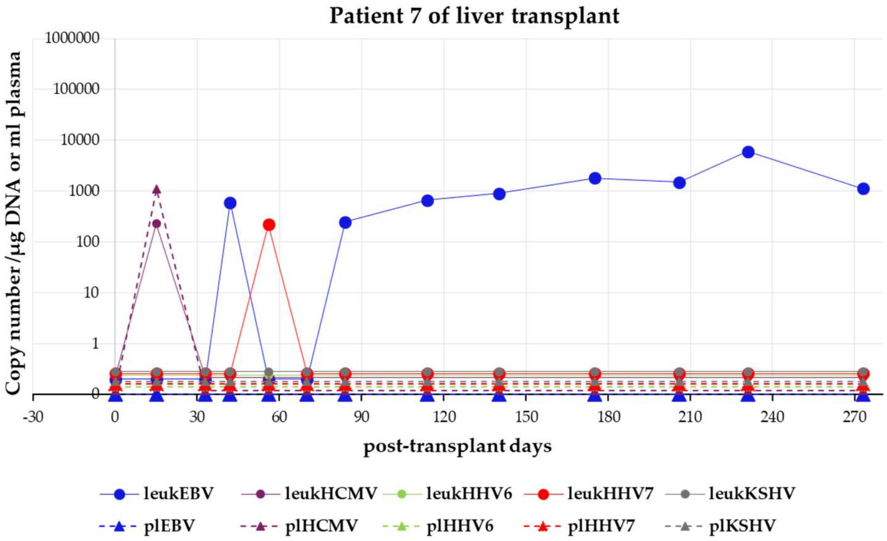
Patient 7 of liver transplant
Copy number/µg DNA or ml plasma
1000000
100000
10000
1000
100
10
1
0
-30
0
30
60
90
120
150
180
210
240
270
post-transplant days
leukEBV
leukHCMV
leukHHV6
leukHHV7
leukKSHV
plEBV
plHCMV
plHHV6
plHHV7
plKSHV

(g)

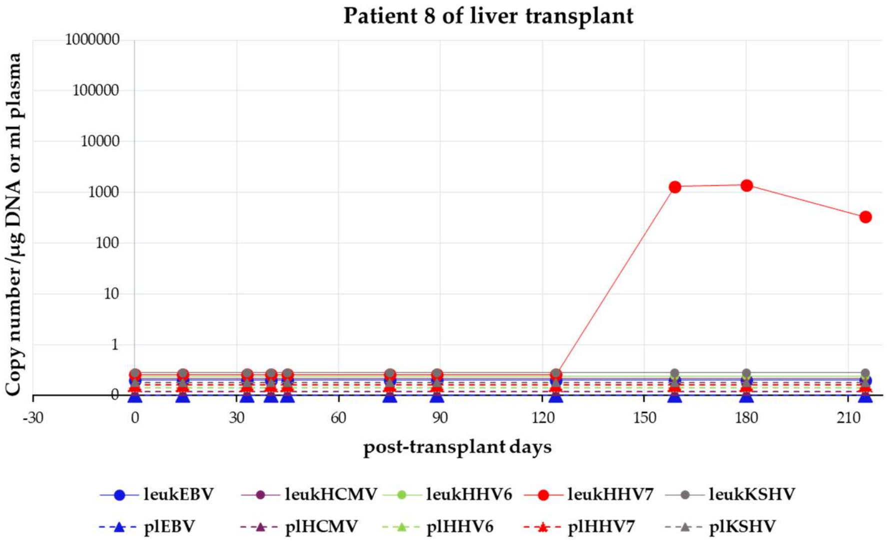
Patient 8 of liver transplant
Copy number/µg DNA or ml plasma
1000000
100000
10000
1000
100
10
1
0
-30
0
30
60
90
120
150
180
210
post-transplant days
leukEBV
leukHCMV
leukHHV6
leukHHV7
leukKSHV
plEBV
plHCMV
plHHV6
plHHV7
plKSHV

(h)

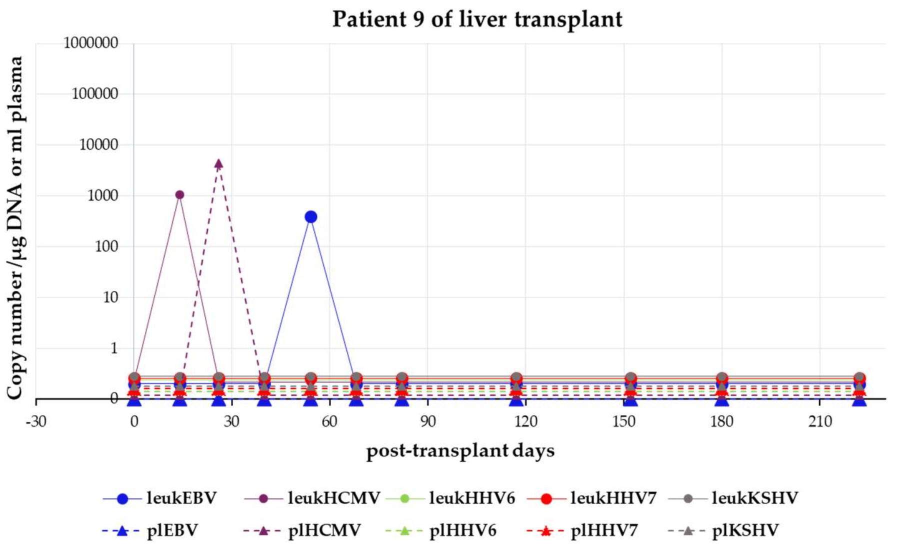

(i)

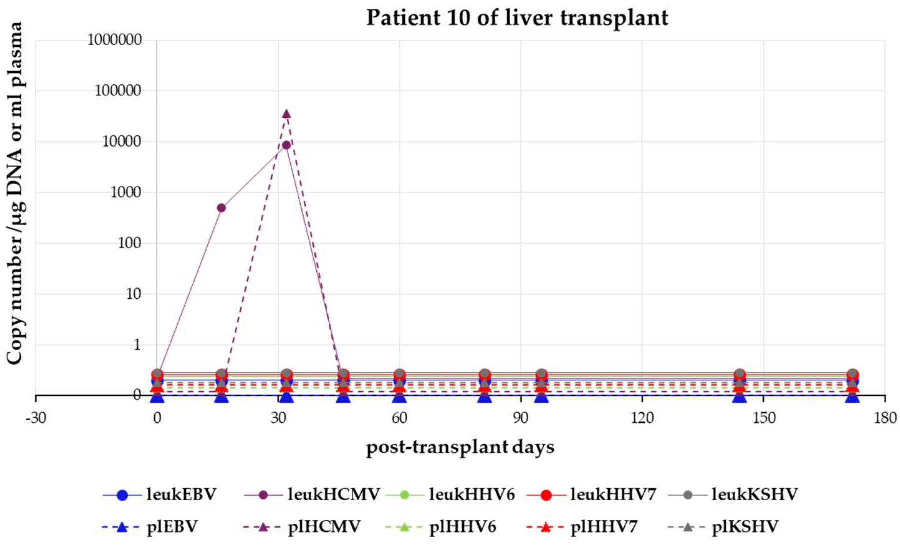

(j)

Supplementary Table 1. Analysis of formation of auto-dimers of the primers

and probes used for detection of EBV, HCMV and β-actin.

**Virus-gene (primer/probe)**	**Maximum G Delta (kcal/mol)**
EBV-BALF5 (F)	-5.12
EBV-BALF5 (R)	-4.64
EBV-BALF5 (P)	-9.89
HCMV- UL123 (F)	-13.19
HCMV- UL123 (R)	-5.7
HCMV- UL123 (P)	-9.89
Endogeneous human gene- β-actin (F)	-4.67
Endogeneous human gene- β-actin (R)	-7.05
Endogeneous human gene- β-actin (P)	-16.03

F: Forward, R: Reverse, P: Probe.

Supplementary Table 2. Analysis of formation of hetero-dimers of the primers and probes used for detection of EBV, HCMV and β-actin.

Virus-gene (primer/probe)/ virus-gene (primer/probe)	Maximum G Delta (kcal/mol)
EBV-BALF5 (F)/EBV-BALF5 (R)	-8.26
EBV-BALF5 (F)/EBV-BALF5 (P)	-3.61
EBV-BALF5 (R)/EBV-BALF5 (P)	-3.89
HCMV- UL123 (F)/HCMV- UL123 (R)	-6.21
HCMV- UL123 (F)/HCMV- UL123 (P)	-6.21
HCMV- UL123 (R)/HCMV- UL123 (P)	-8.2
Endogeneous human gene- β-actin (F)/Endogeneous human gene- β-actin (R)	-6.62
Endogeneous human gene- β-actin (F)/Endogeneous human gene- β-actin (P)	-6.68
Endogeneous human gene- β-actin (R)/Endogeneous human gene- β-actin (P)	-5.09
EBV-BALF 5 (F)/HCMV- UL123 (F)	-6.21
EBV-BALF 5 (F)/HCMV- UL123 (R)	-8.19
EBV-BALF 5 (F)/HCMV- UL123 (P)	-6.24
EBV-BALF 5 (R)/HCMV- UL123 (F)	-5.02
EBV-BALF 5 (R)/HCMV- UL123 (R)	-6.59
EBV-BALF 5 (R)/HCMV- UL123 (P)	-4.67
EBV-BALF 5 (P)/HCMV- UL123 (F)	-6.21
EBV-BALF 5 (P)/HCMV- UL123 (R)	-4.26
EBV-BALF 5 (P)/HCMV- UL123 (P)	-9.89
Endogeneous human gene- β-actin(F)/EBV-BALF5 (F)	-7.81
Endogeneous human gene- β-actin (F)/EBV-BALF5 (R)	-9.83
Endogeneous human gene- β-actin (F)/EBV-BALF5 (P)	-6.78
Endogeneous human gene- β-actin (R)/EBV-BALF5 (F)	-6.59
Endogeneous human gene- β-actin (R)/EBV-BALF5 (R)	-10.02
Endogeneous human gene- β-actin (R)/EBV-BALF5 (P)	-6.53
Endogeneous human gene- β-actin (P)/EBV-BALF5 (F)	-7.81
Endogeneous human gene- β-actin (P)/EBV-BALF5 (R)	-6.68
Endogeneous human gene- β-actin (P)/EBV-BALF5 (P)	-6.44
Endogeneous human gene- β-actin (F)/HCMV- UL123 (F)	-6.62
Endogeneous human gene- β-actin (F)/HCMV- UL123 (R)	-10.37
Endogeneous human gene- β-actin (F)/HCMV- UL123 (P)	-11.36
Endogeneous human gene- β-actin (R)/HCMV- UL123 (F)	-5.09
Endogeneous human gene- β-actin (R)/HCMV- UL123 (R)	-3.42
Endogeneous human gene- β-actin (R)/HCMV- UL123 (P)	-6.95
Endogeneous human gene- β-actin (P)/HCMV- UL123 (F)	-6.21
Endogeneous human gene- β-actin (P)/HCMV- UL123 (R)	-7.81
Endogeneous human gene- β-actin (P)/HCMV- UL123 (P)	-7.81

F: Forward, R: Reverse, P: Probe.

Supplementary Table 3. Analysis of formation of auto-dimers of the primers and probes used for detection of HHV6, HHV7 and KSHV.

**Virus-gene (primer/probe)**	**Maximum G Delta (kcal/mol)**
HHV6-U31 (F)	-3.61
HHV6-U31 (R)	-3.61
HHV6-U31 (P)	-4.89
HHV7-U57 (F)	-4.87
HHV7-U57 (R)	-8.74
HHV7-U57 (P)	-13.19
KSHV-LANA (F)	-5.99
KSHV-LANA (R)	-3.61
KSHV-LANA (P)	-3.61

F: Forward, R: Reverse, P: Probe.

Supplementary Table 4. Analysis of formation of hetero-dimers of the primers and probes used for detection of HHV6, HHV7 and KSHV.

Virus-gene (primer/probe)/ virus-gene (primer/probe)	Maximum G Delta (kcal/mol)
HHV6-U31 (F)/HHV6-U31 (R)	-3.61
HHV6-U31 (F)/HHV6-U31 (P)	-3.55
HHV6-U31 (R)/HHV6-U31 (P)	-9.76
HHV7-U57 (F)/HHV7-U57 (R)	-4.64
HHV7-U57 (F)/HHV7-U57 (P)	-5.24
HHV7-U57 (R)/HHV7-U57 (P)	-5.24
KSHV-LANA (F)/KSHV-LANA (R)	-6.97
KSHV-LANA (F)/KSHV-LANA (P)	-8.09
KSHV-LANA (R)/KSHV-LANA (P)	-8.09
HHV6-U31 (F)/HHV7-U57 (F)	-4.52
HHV6-U31 (F)/HHV7-U57 (R)	-5.19
HHV6-U31 (F)/HHV7-U57 (P)	-5.19
HHV6-U31 (R)/HHV7-U57 (F)	-3.61
HHV6-U31 (R)/HHV7-U57 (R)	-5.12
HHV6-U31 (R)/HHV7-U57 (P)	-8.16
HHV6-U31 (P)/HHV7-U57 (F)	-6.82
HHV6-U31 (P)/HHV7-U57 (R)	-6.69
HHV6-U31 (P)/HHV7-U57 (P)	-8.16
HHV6-U31 (F)/KSHV-LANA (F)	-6.14
HHV6-U31 (F)/KSHV-LANA (R)	-4.65
HHV6-U31 (F)/KSHV-LANA (P)	-7.48
HHV6-U31 (R)/KSHV-LANA (F)	-6.53
HHV6-U31 (R)/KSHV-LANA (R)	-6.53
HHV6-U31 (R)/KSHV-LANA (P)	-3.61
HHV6-U31 (P)/KSHV-LANA (F)	-5.02
HHV6-U31 (P)/KSHV-LANA (R)	-1.95
HHV6-U31 (P)/KSHV-LANA (P)	-6.5
HHV7-U57 (F)/KSHV-LANA (F)	-6.62
HHV7-U57 (F)/KSHV-LANA (R)	-8.19
HHV7-U57 (F)/KSHV-LANA (P)	-8.29
HHV7-U57 (R)/KSHV-LANA (F)	-6.24
HHV7-U57 (R)/KSHV-LANA (R)	-3.89
HHV7-U57 (R)/KSHV-LANA (P)	-6.68
HHV7-U57 (P)/KSHV-LANA (F)	-5.02
HHV7-U57 (P)/KSHV-LANA (R)	-6.97
HHV7-U57 (P)/KSHV-LANA (P)	-6.5

F: Forward, R: Reverse, P: Probe.
