# Supplementary figures and images for: Simultaneous Detection of Beta and Gamma Human Herpesviruses by Multiplex qPCR Reveals Simple Infection and Coinfection Episodes Increasing Risk for Graft Rejection in Solid Organ Transplantation
Source: Viruses. 2018 Dec 19;10(12):730. doi: 10.3390/v10120730 (PMC6316002; doi:10.3390/v10120730)

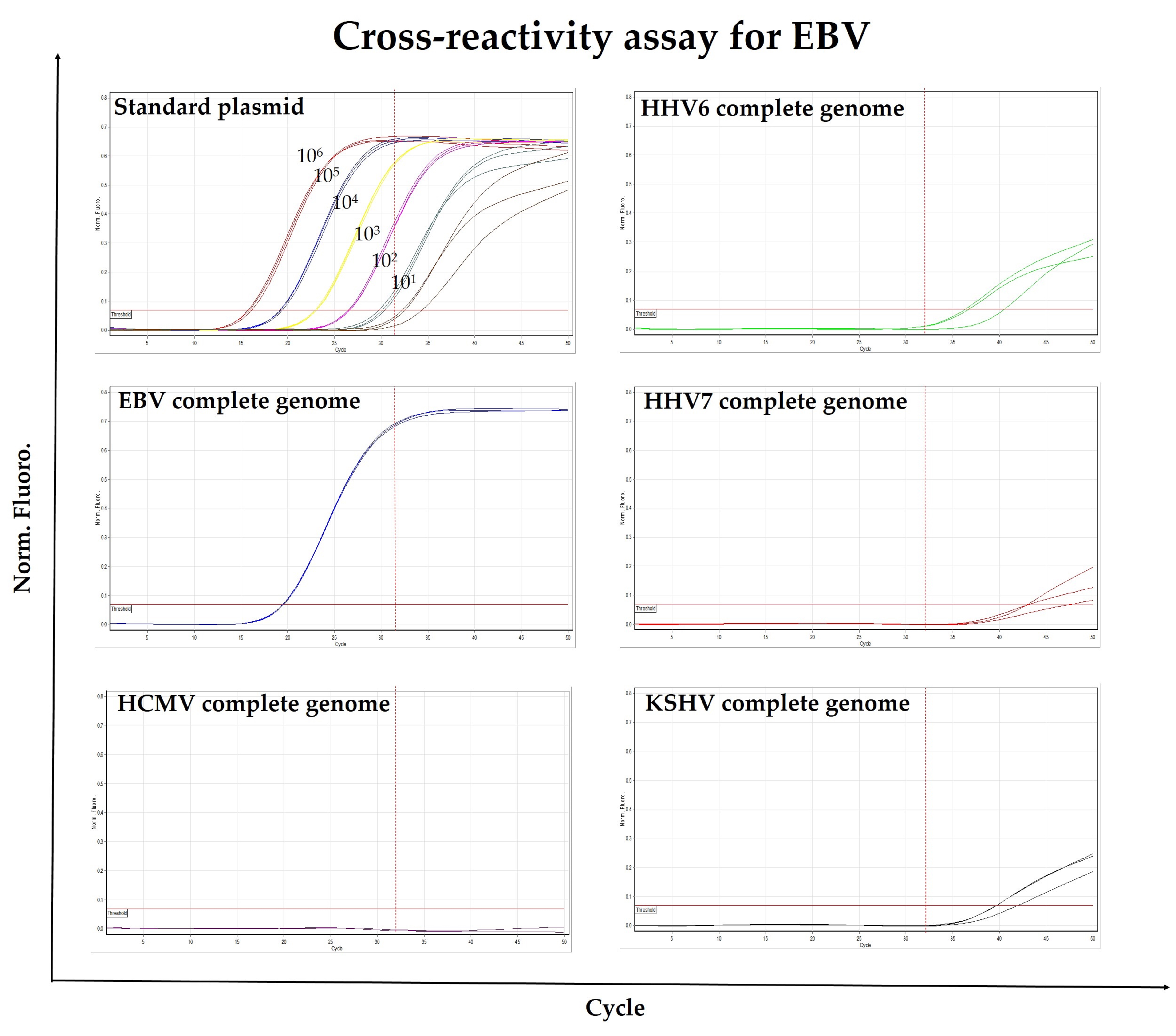

Supplement: Supplementary file 1 [file viruses-10-00730-s001.zip › Supplementary 1a.jpg]

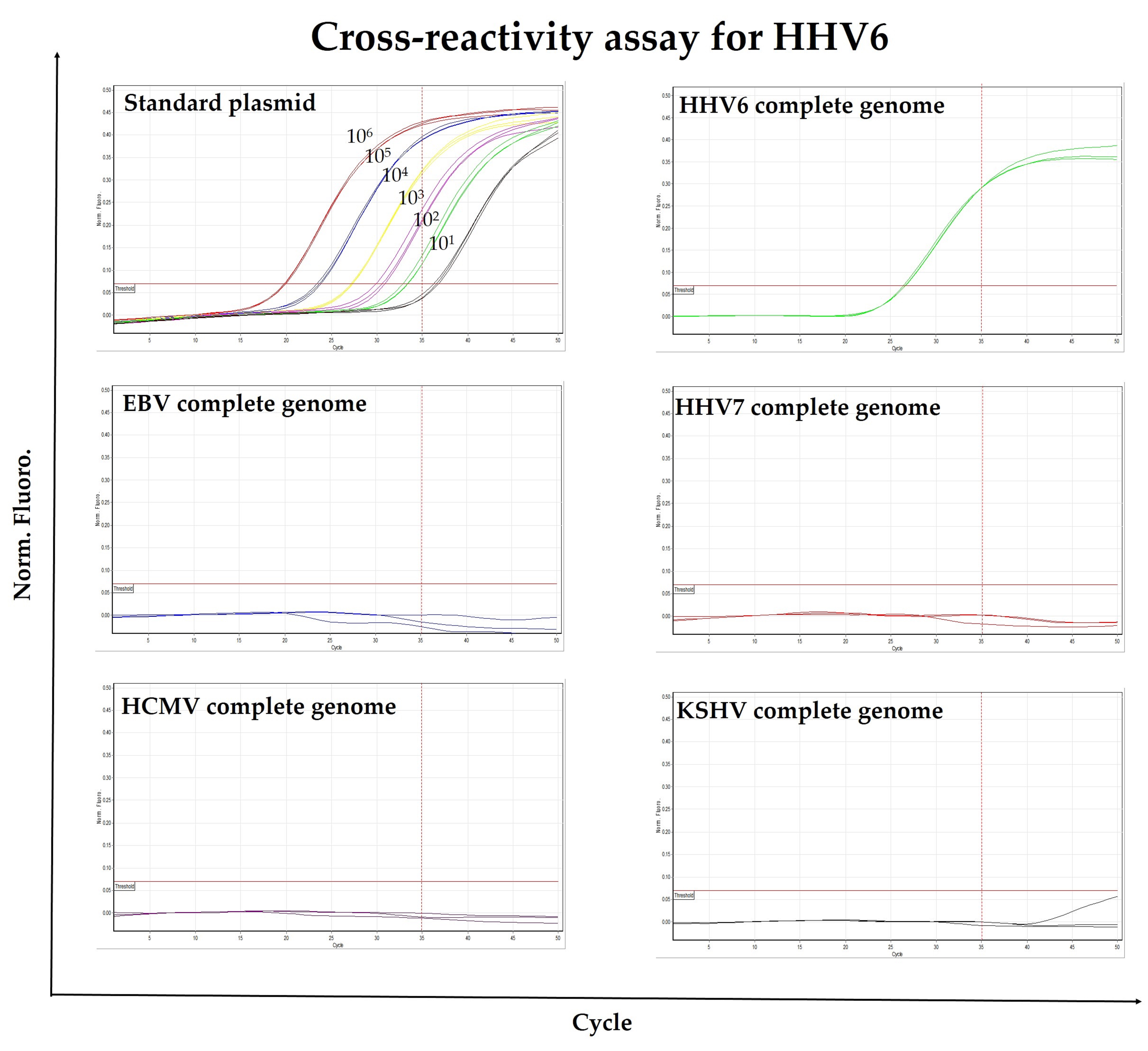

Supplement: Supplementary file 1 [file viruses-10-00730-s001.zip › Supplementary 1b.jpg]

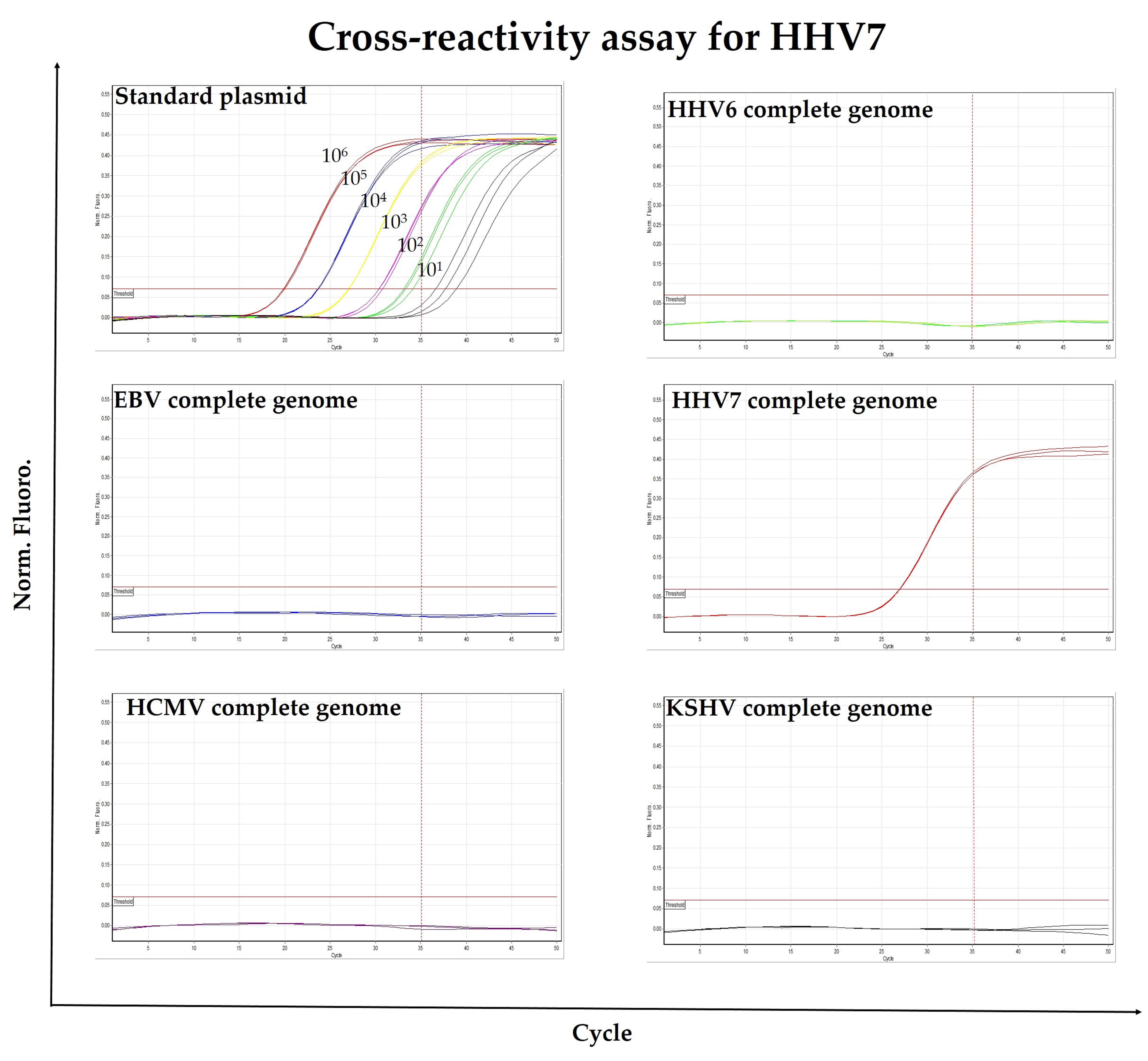

Supplement: Supplementary file 1 [file viruses-10-00730-s001.zip › Supplementary 1c.jpg]

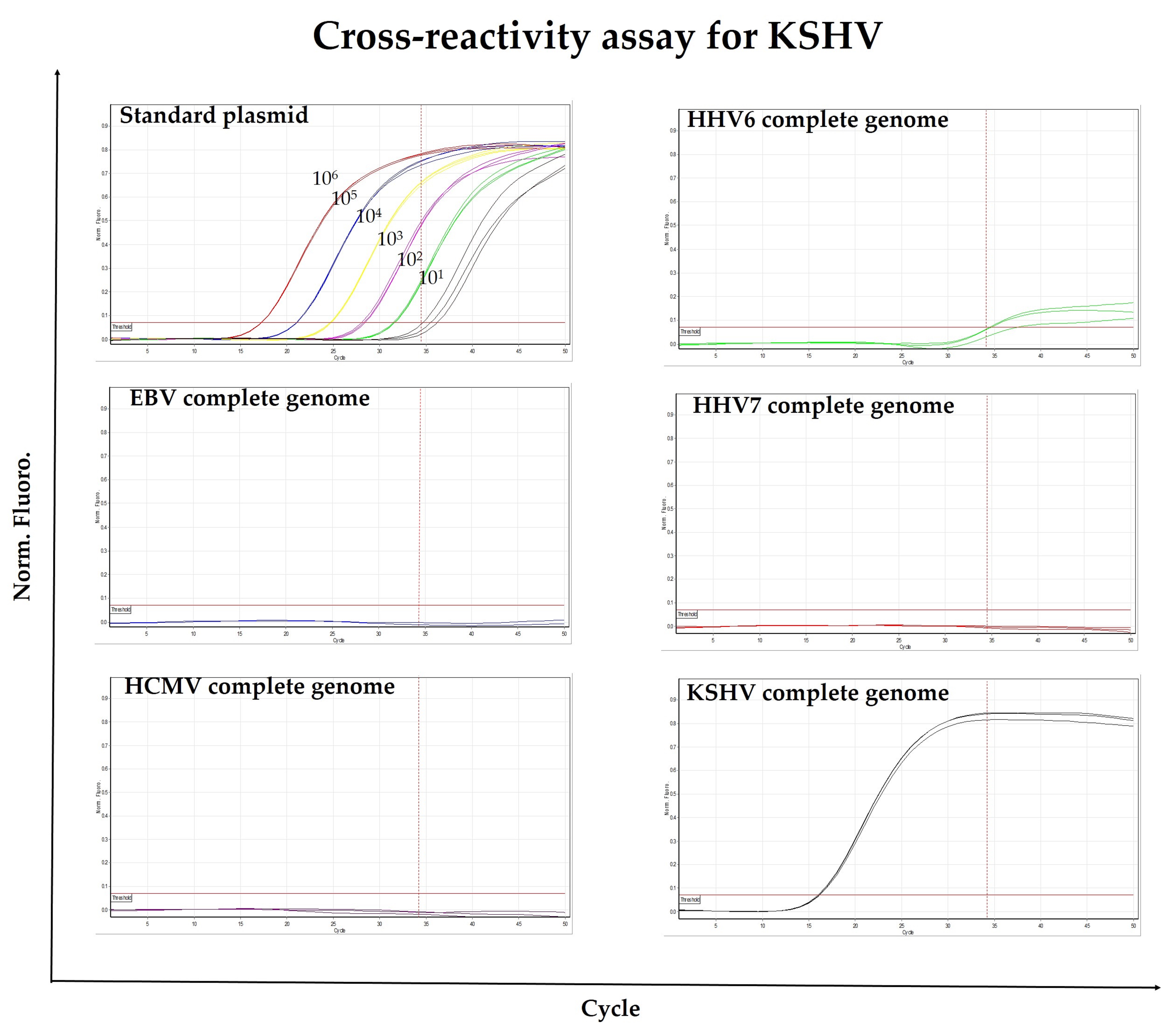

Supplement: Supplementary file 1 [file viruses-10-00730-s001.zip › Supplementary 1d.jpg]

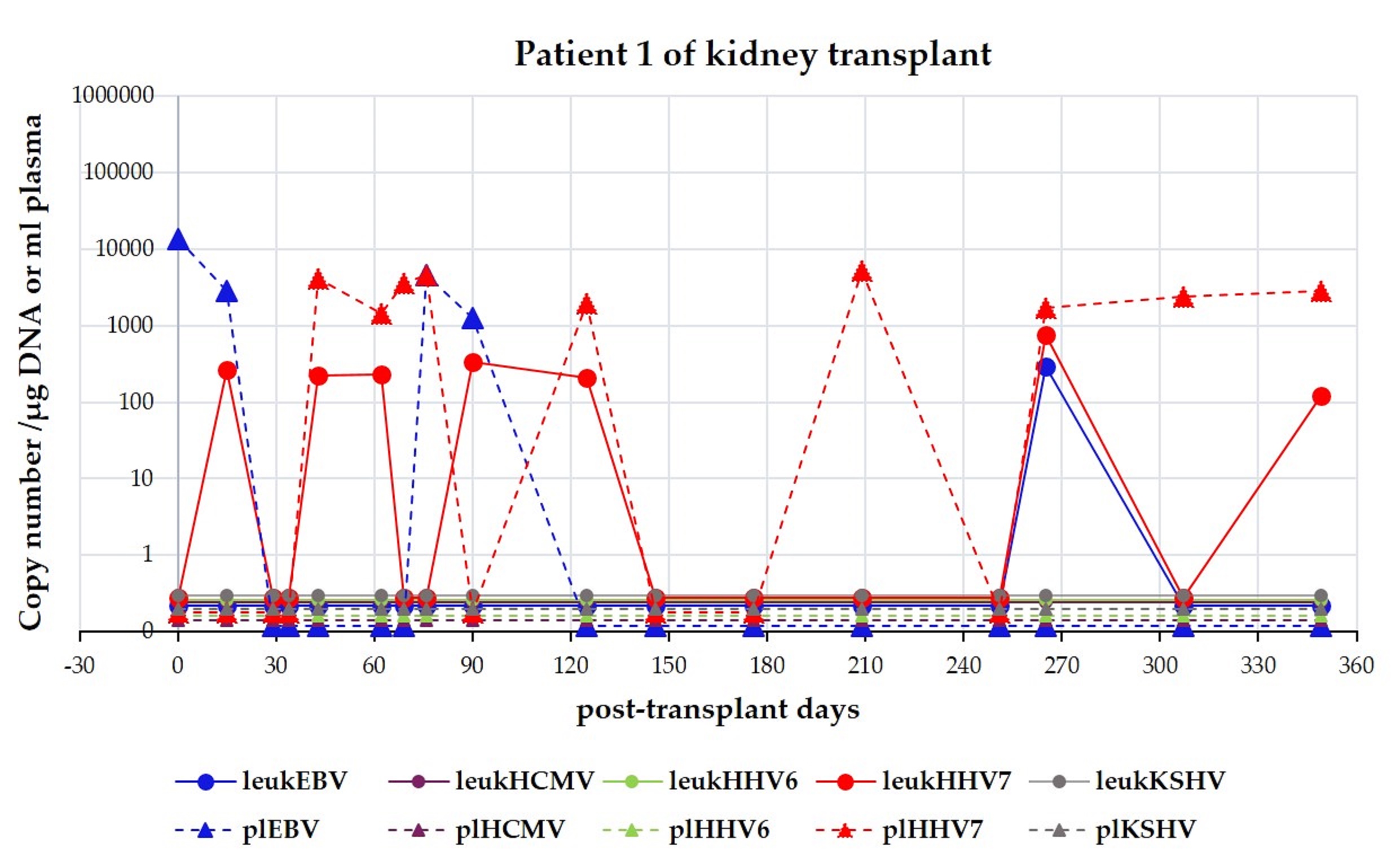

Supplement: Supplementary file 1 [file viruses-10-00730-s001.zip › Supplemntary 2a.jpg]

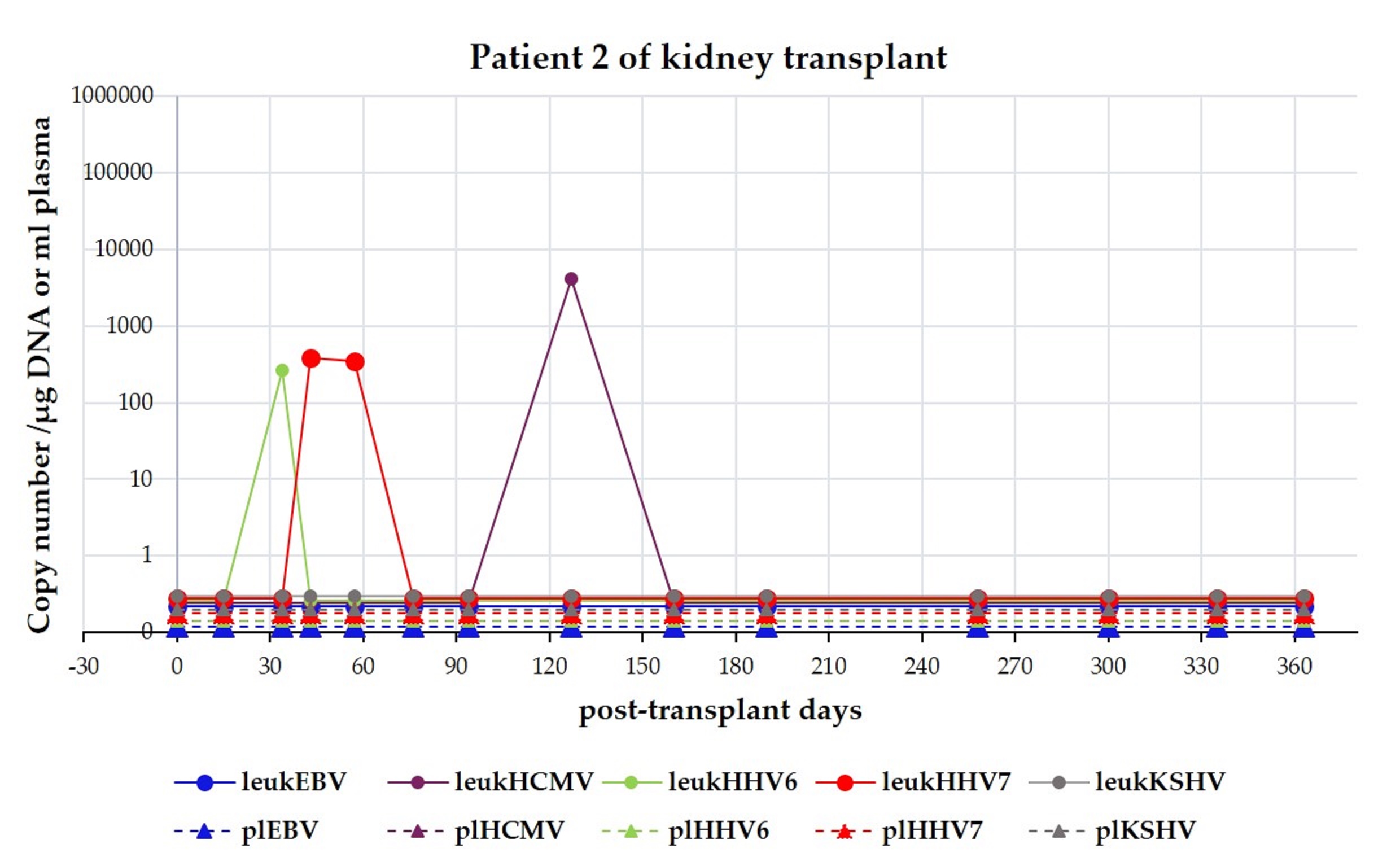

Supplement: Supplementary file 1 [file viruses-10-00730-s001.zip › Supplemntary 2b.jpg]

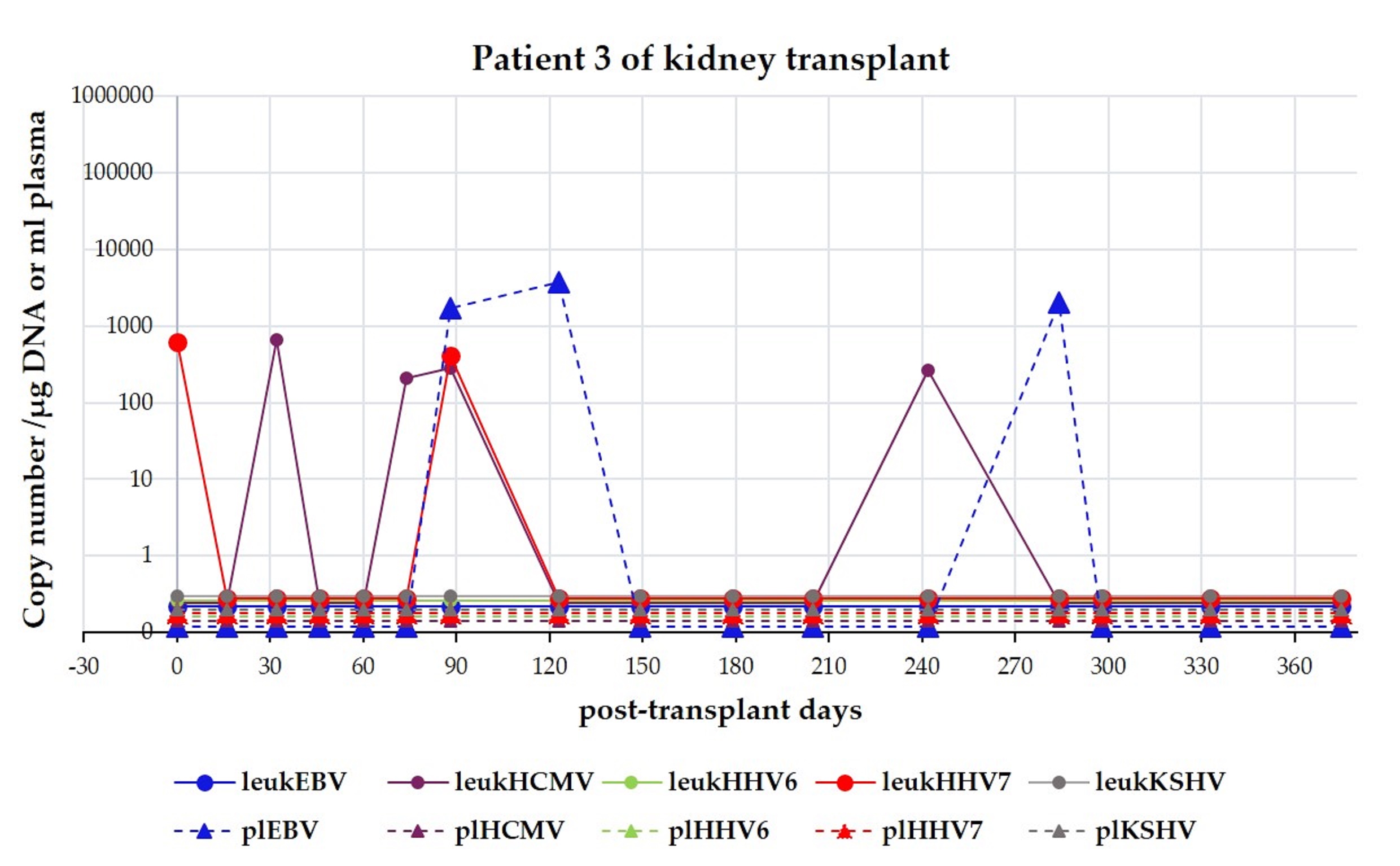

Supplement: Supplementary file 1 [file viruses-10-00730-s001.zip › Supplemntary 2c.jpg]

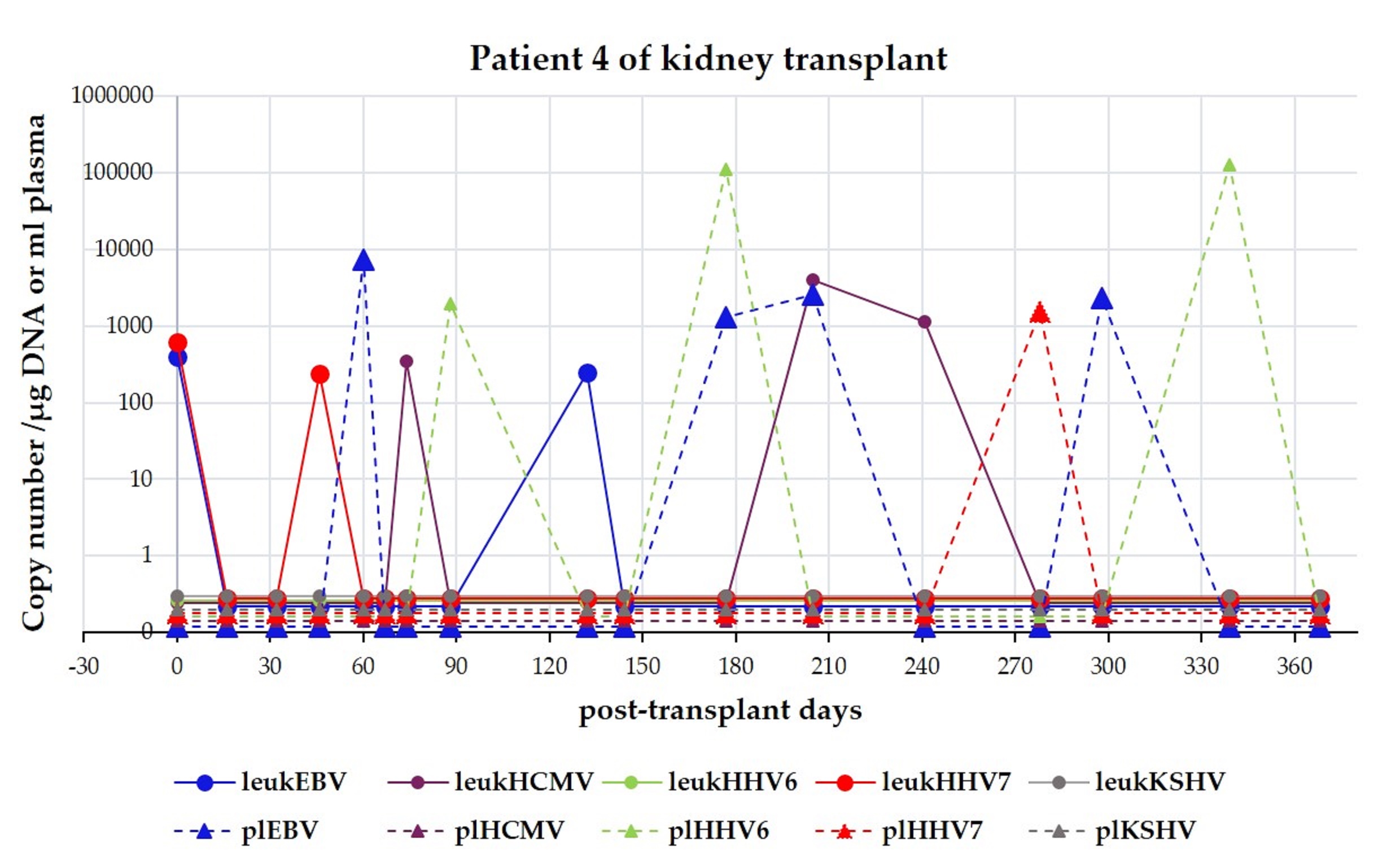

Supplement: Supplementary file 1 [file viruses-10-00730-s001.zip › Supplemntary 2d.jpg]

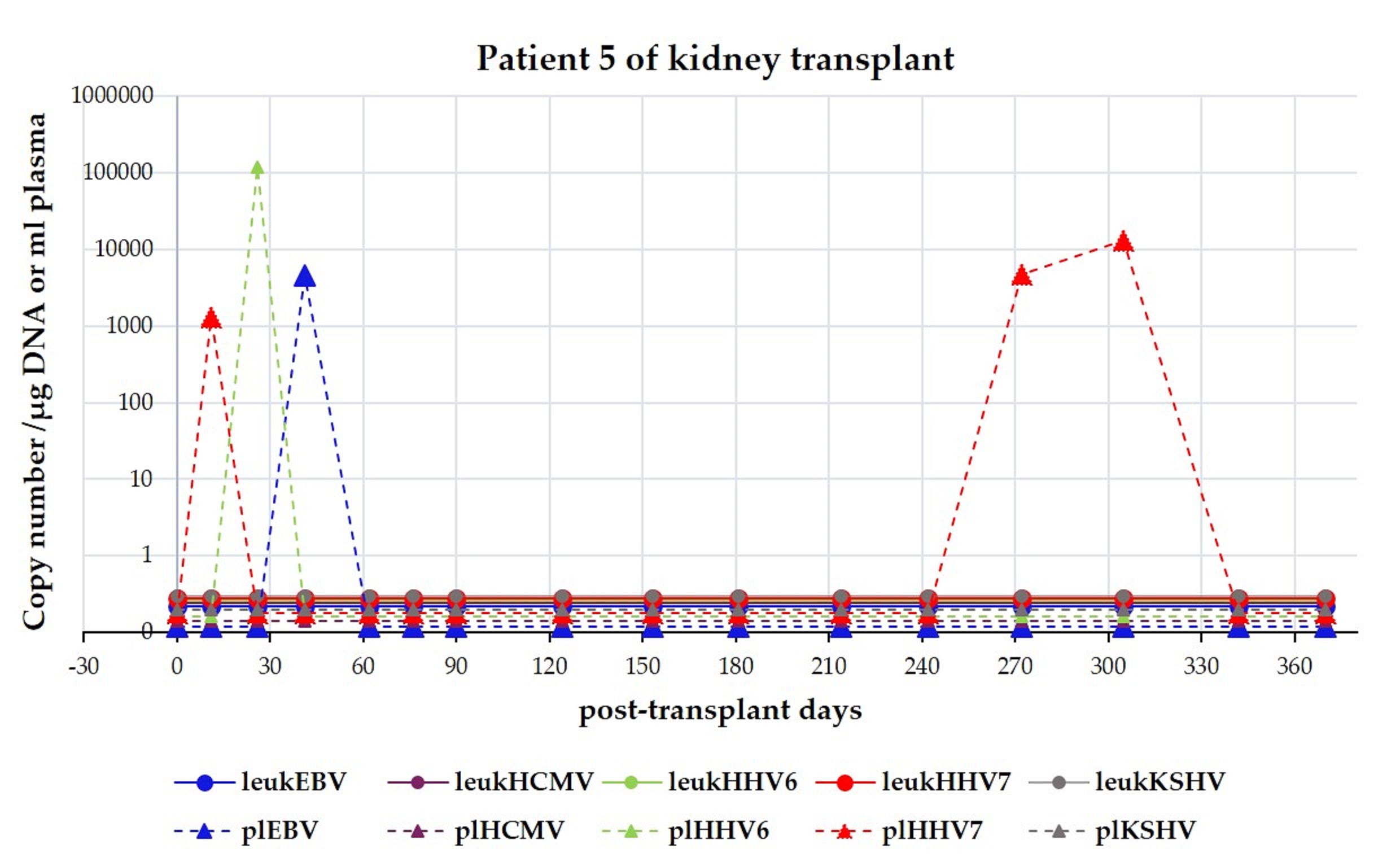

Supplement: Supplementary file 1 [file viruses-10-00730-s001.zip › Supplemntary 2e.jpg]

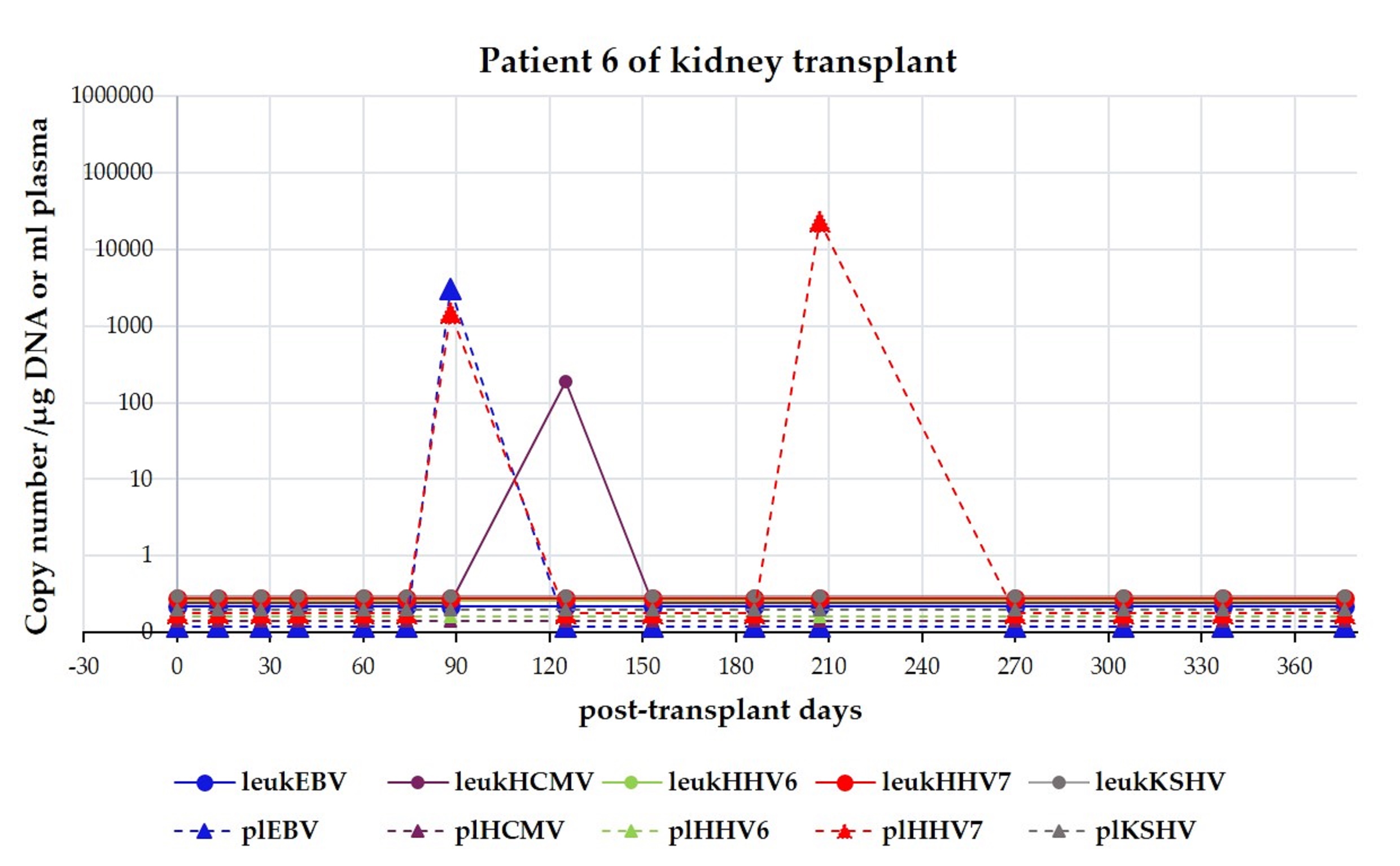

Supplement: Supplementary file 1 [file viruses-10-00730-s001.zip › Supplemntary 2f.jpg]

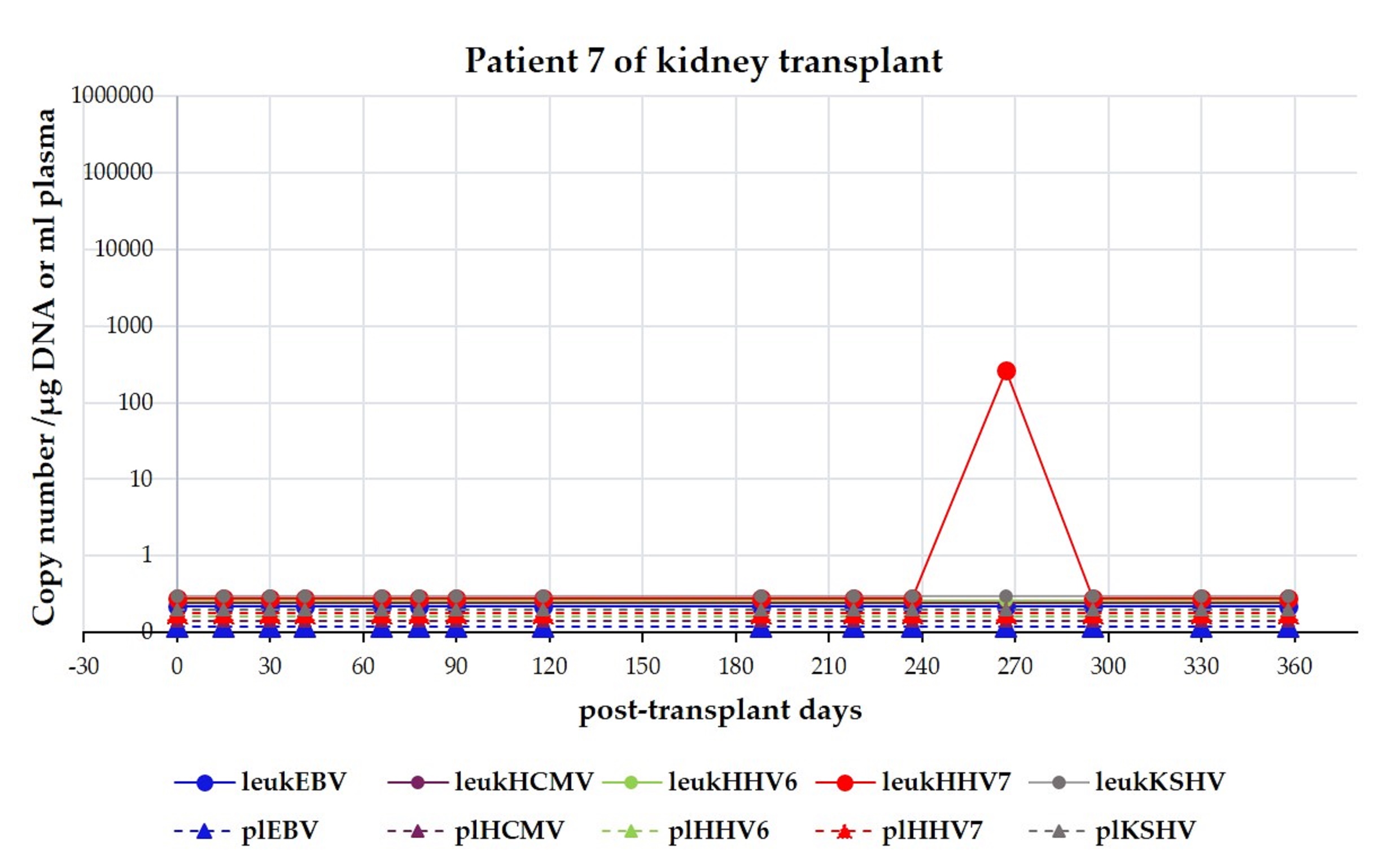

Supplement: Supplementary file 1 [file viruses-10-00730-s001.zip › Supplemntary 2g.jpg]

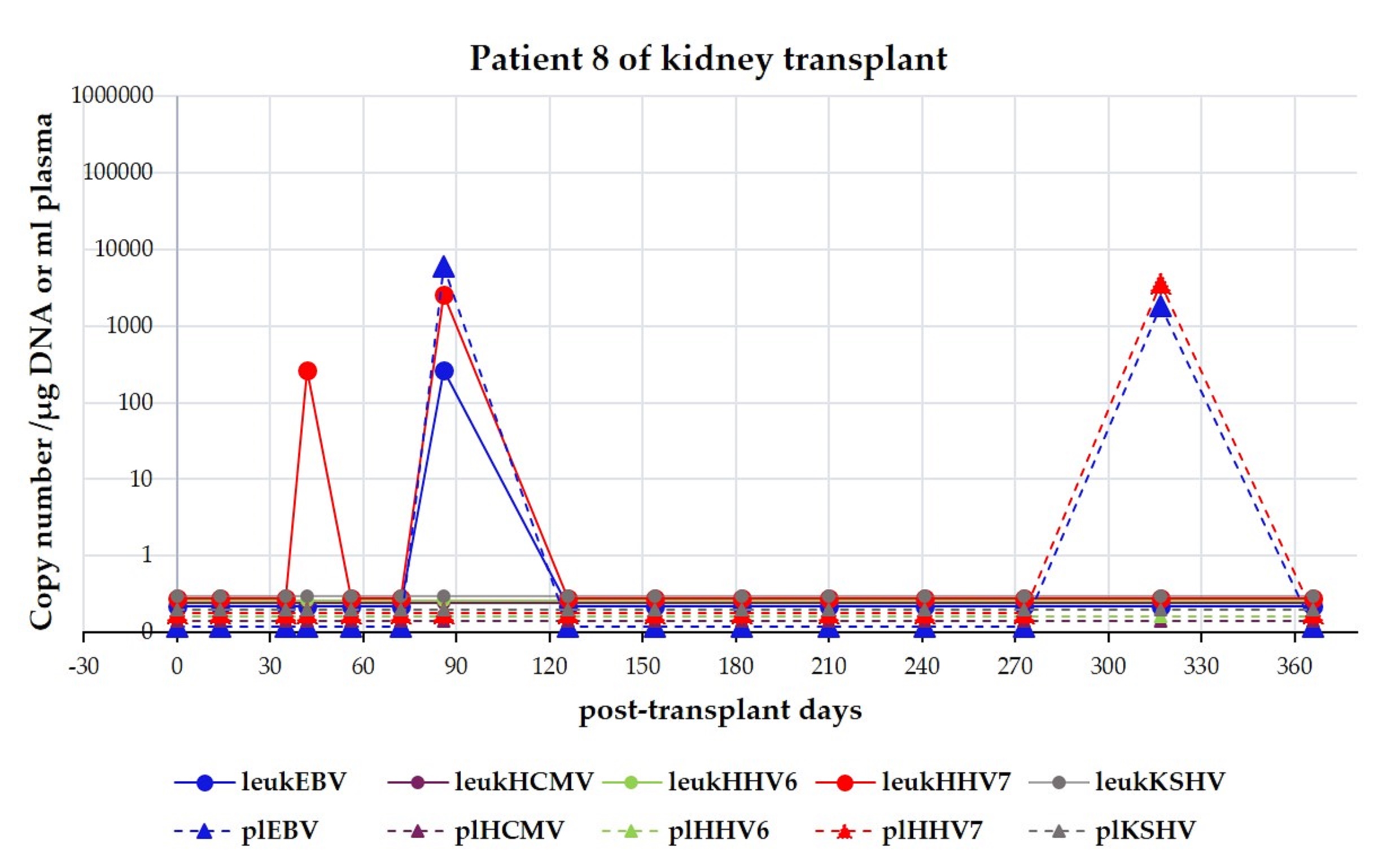

Supplement: Supplementary file 1 [file viruses-10-00730-s001.zip › Supplemntary 2h.jpg]

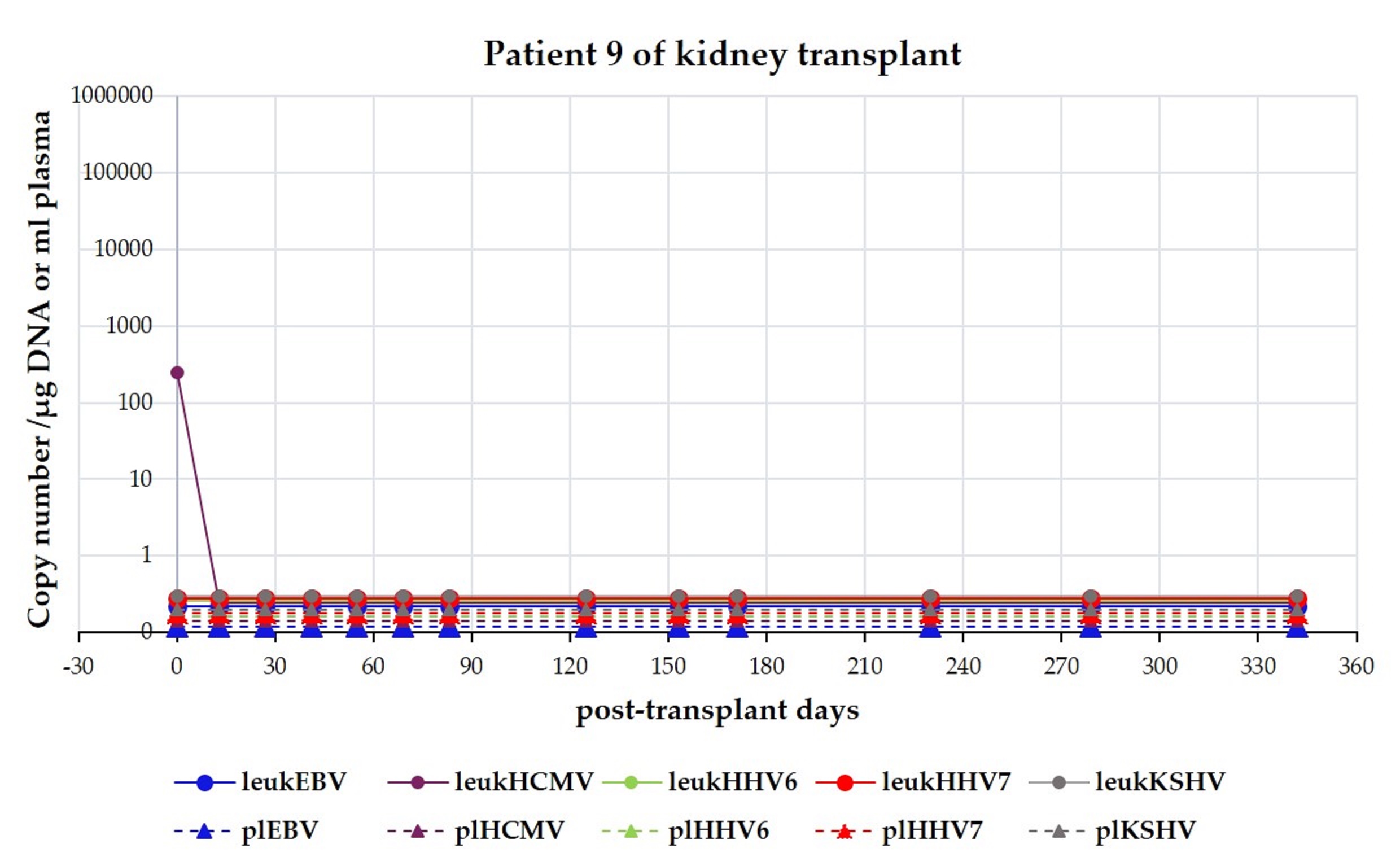

Supplement: Supplementary file 1 [file viruses-10-00730-s001.zip › Supplemntary 2i.jpg]

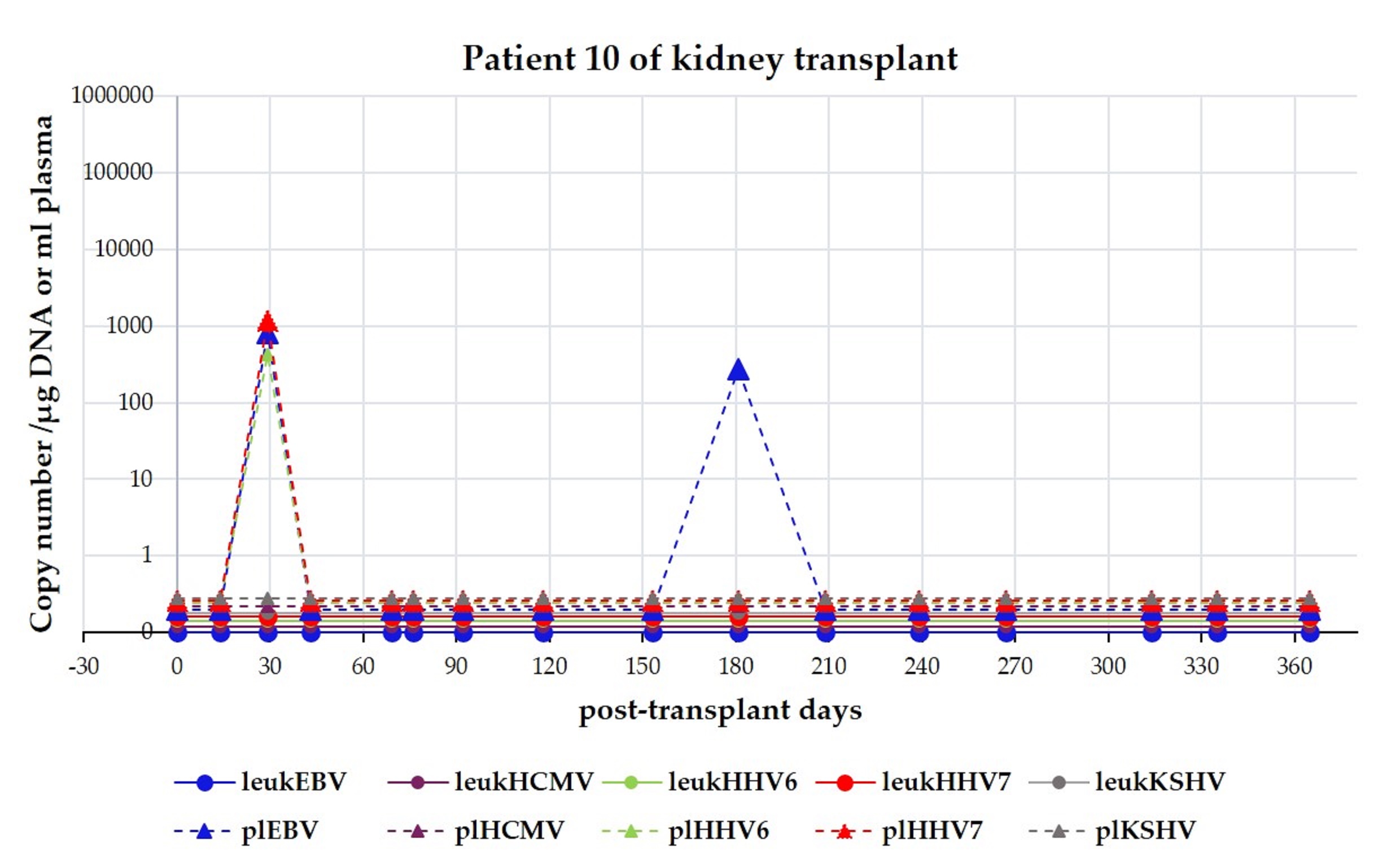

Supplement: Supplementary file 1 [file viruses-10-00730-s001.zip › Supplemntary 2j.jpg]

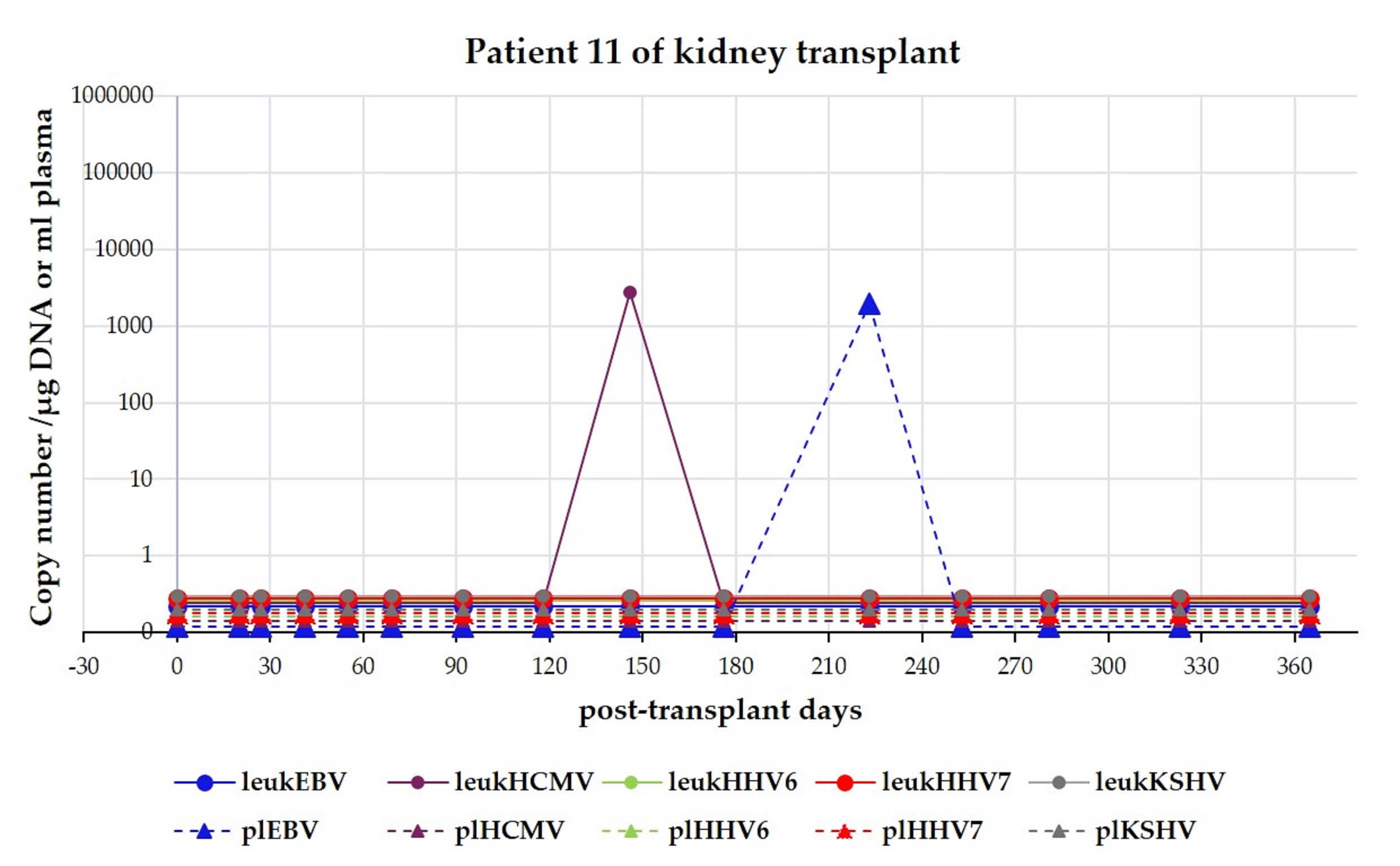

Supplement: Supplementary file 1 [file viruses-10-00730-s001.zip › Supplemntary 2k.jpg]

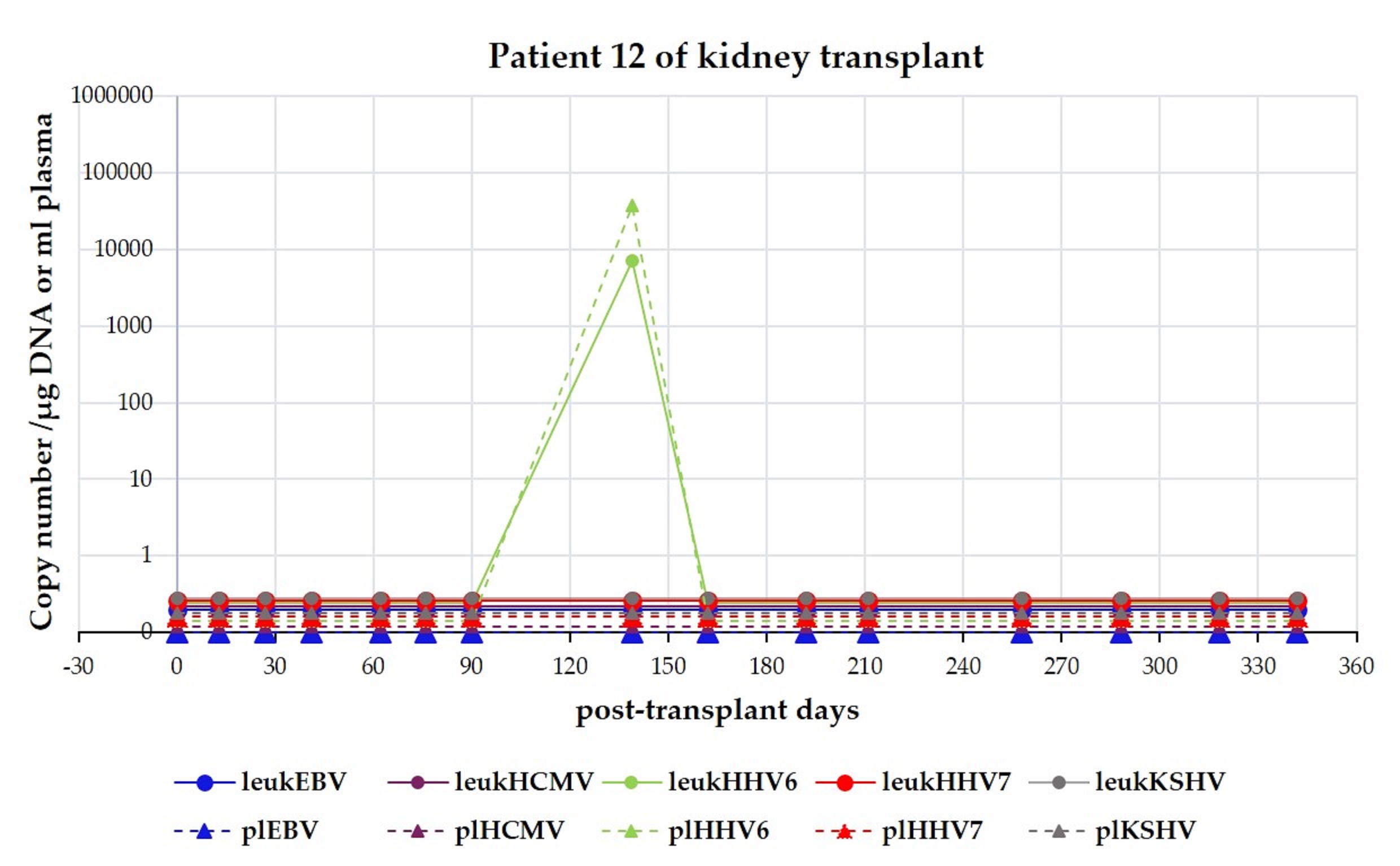

Supplement: Supplementary file 1 [file viruses-10-00730-s001.zip › Supplemntary 2l.jpg]

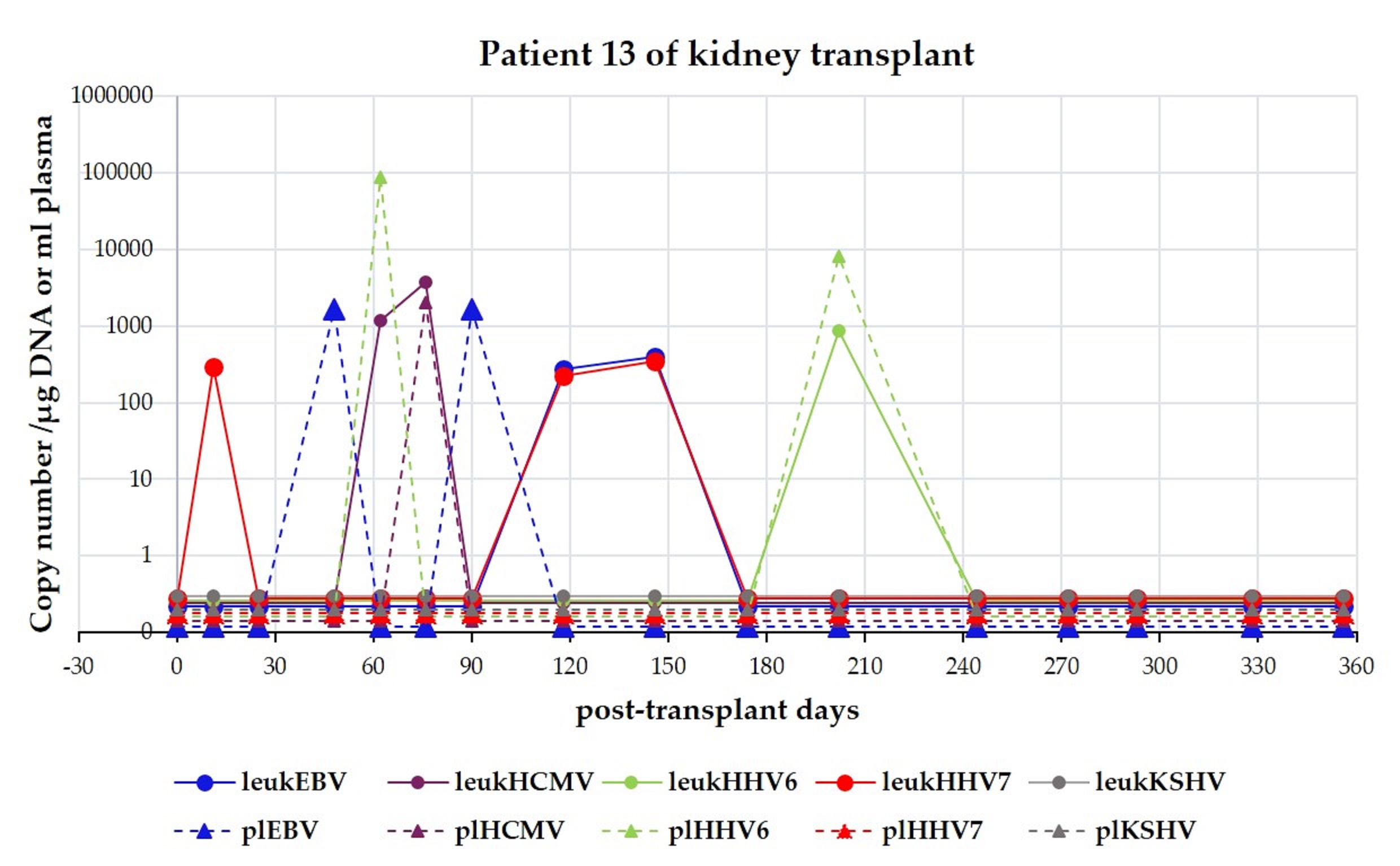

Supplement: Supplementary file 1 [file viruses-10-00730-s001.zip › Supplemntary 2m.jpg]

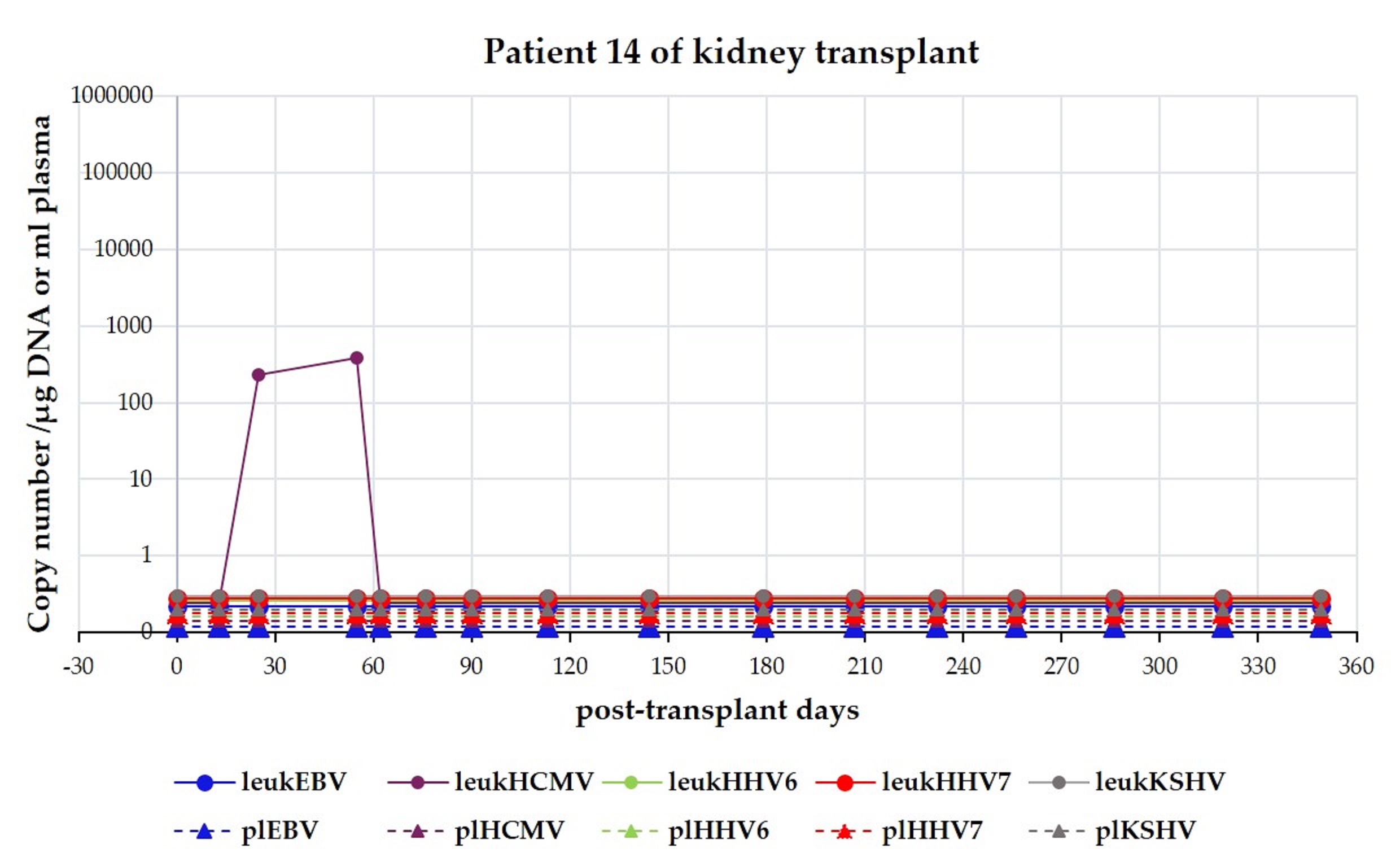

Supplement: Supplementary file 1 [file viruses-10-00730-s001.zip › Supplemntary 2n.jpg]

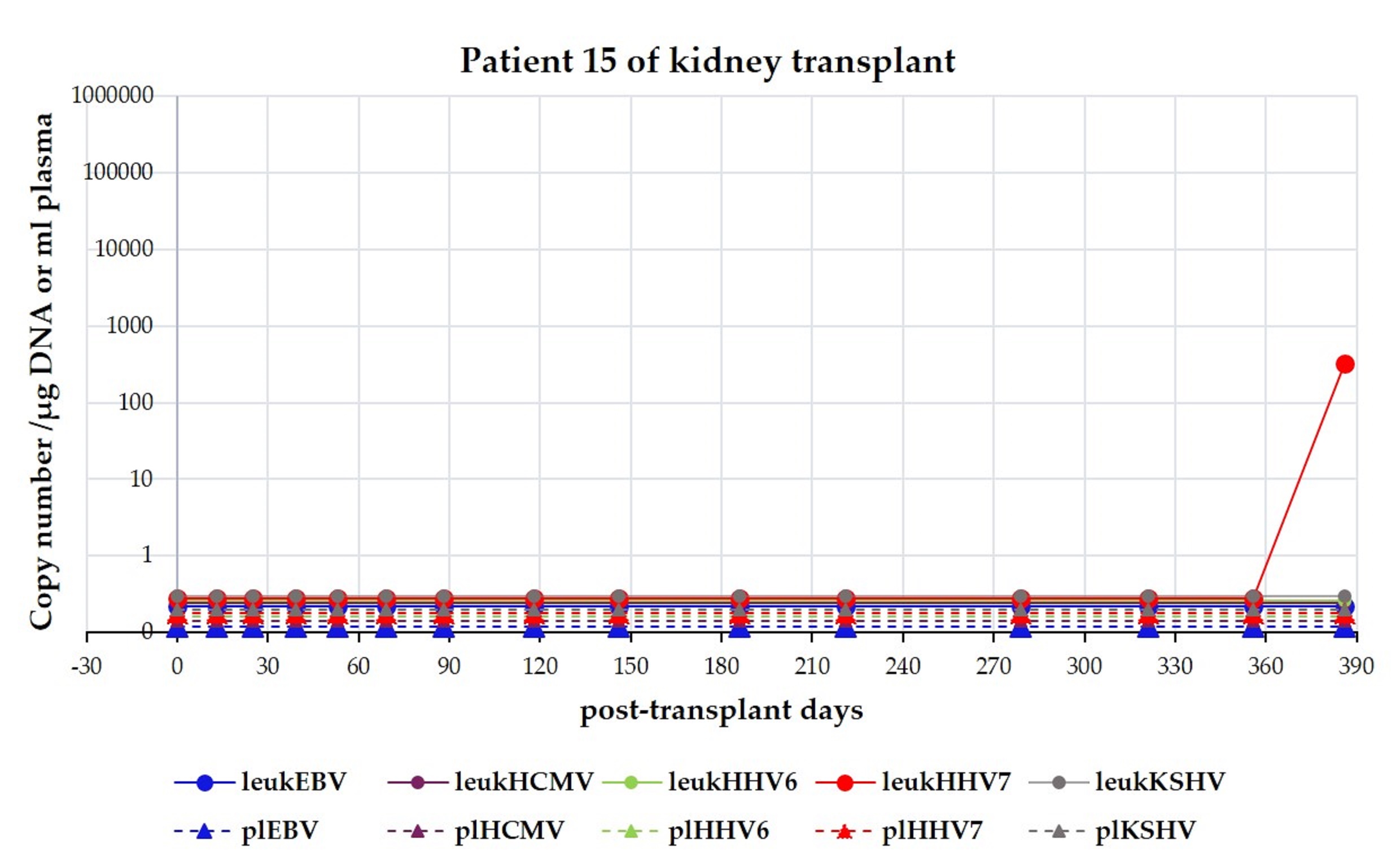

Supplement: Supplementary file 1 [file viruses-10-00730-s001.zip › Supplemntary 2o.jpg]

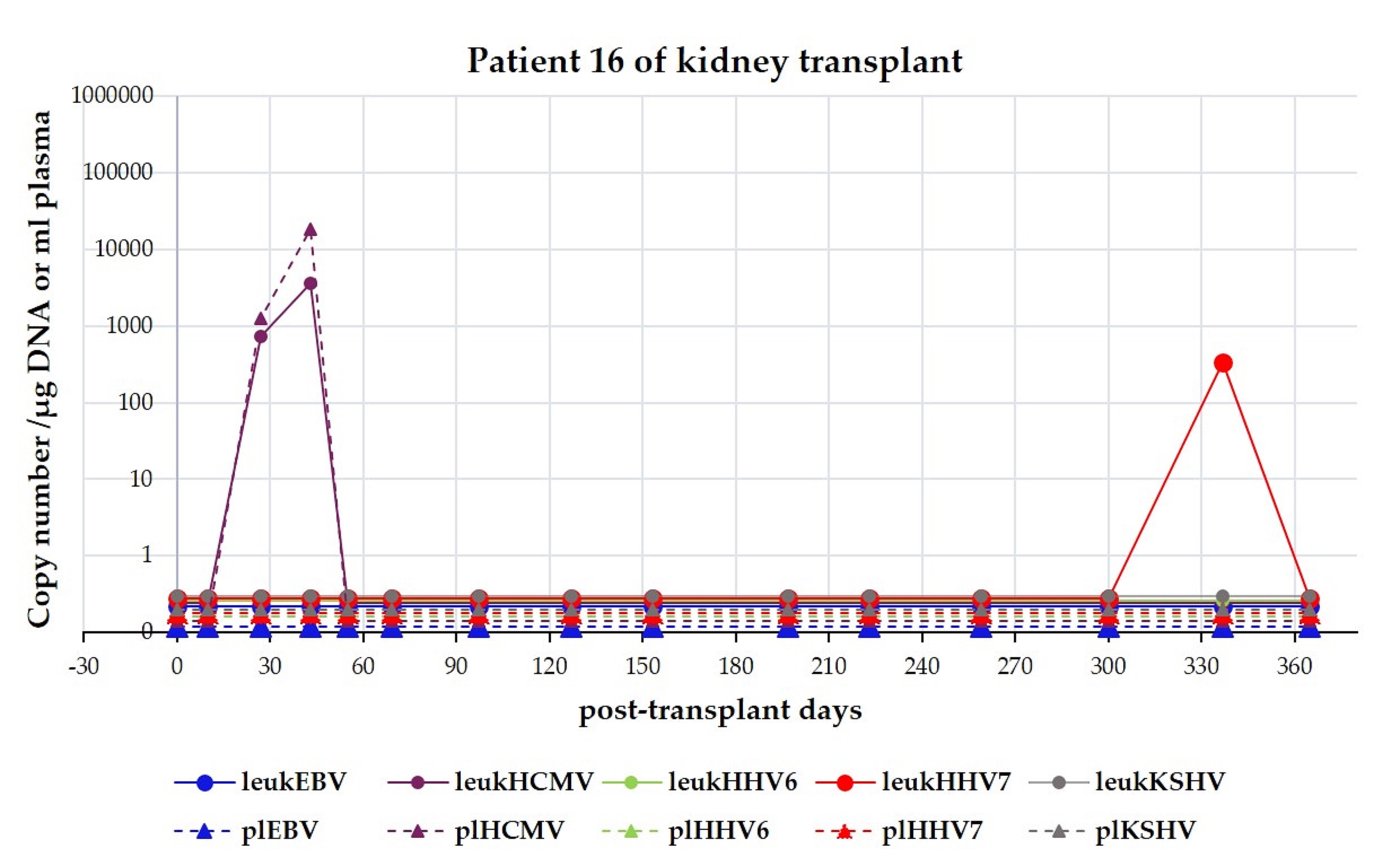

Supplement: Supplementary file 1 [file viruses-10-00730-s001.zip › Supplemntary 2p.jpg]

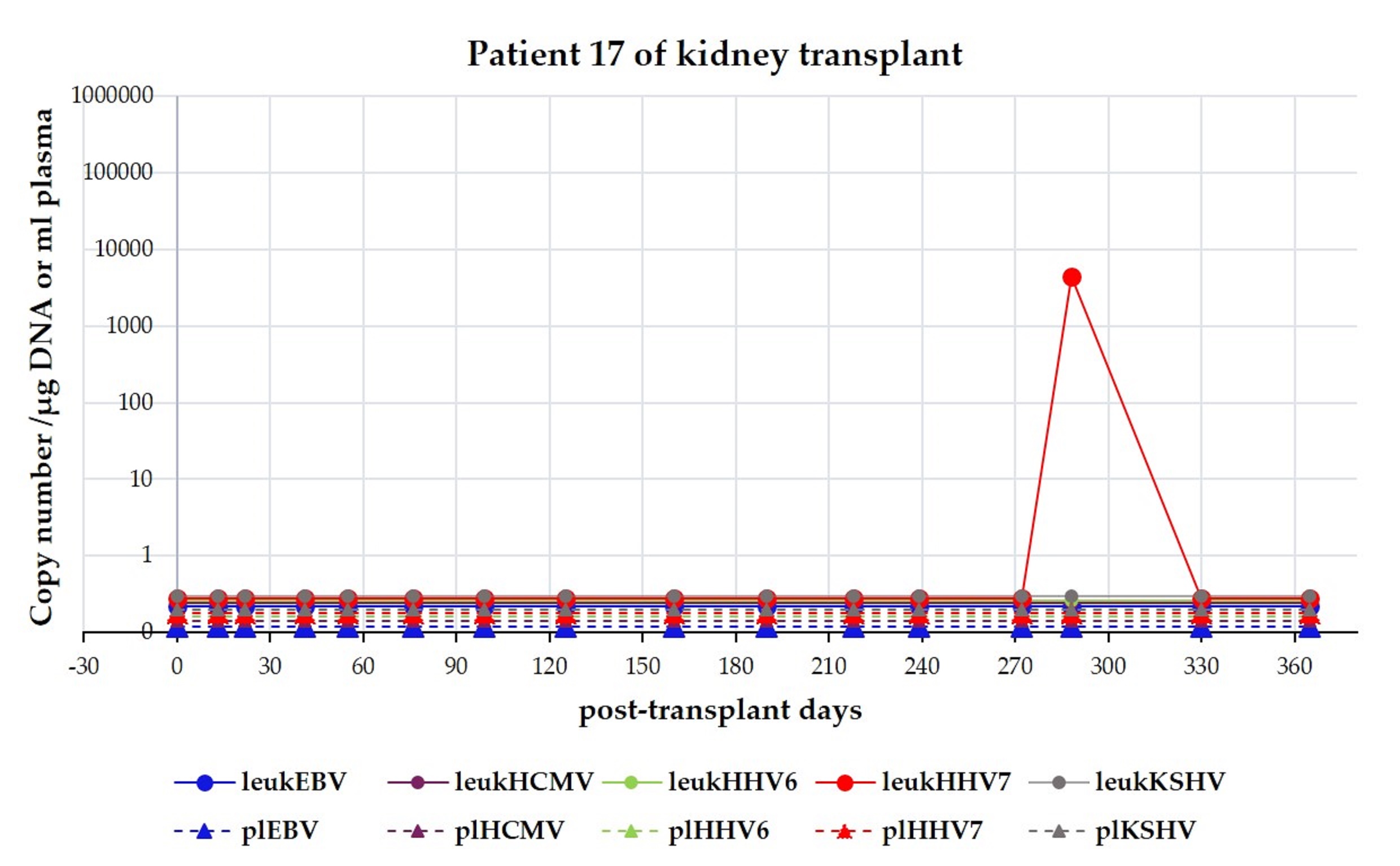

Supplement: Supplementary file 1 [file viruses-10-00730-s001.zip › Supplemntary 2q.jpg]

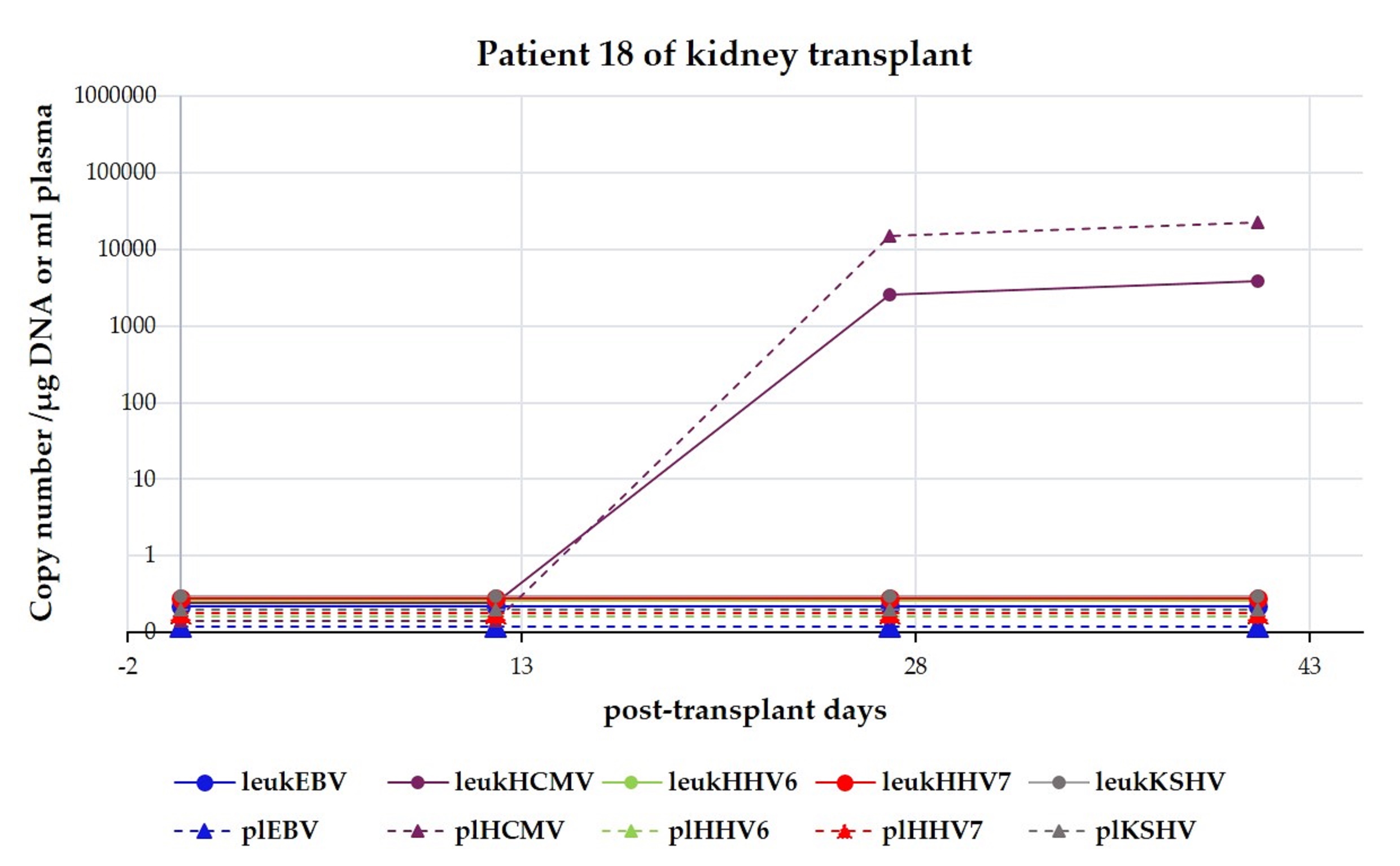

Supplement: Supplementary file 1 [file viruses-10-00730-s001.zip › Supplemntary 2r.jpg]

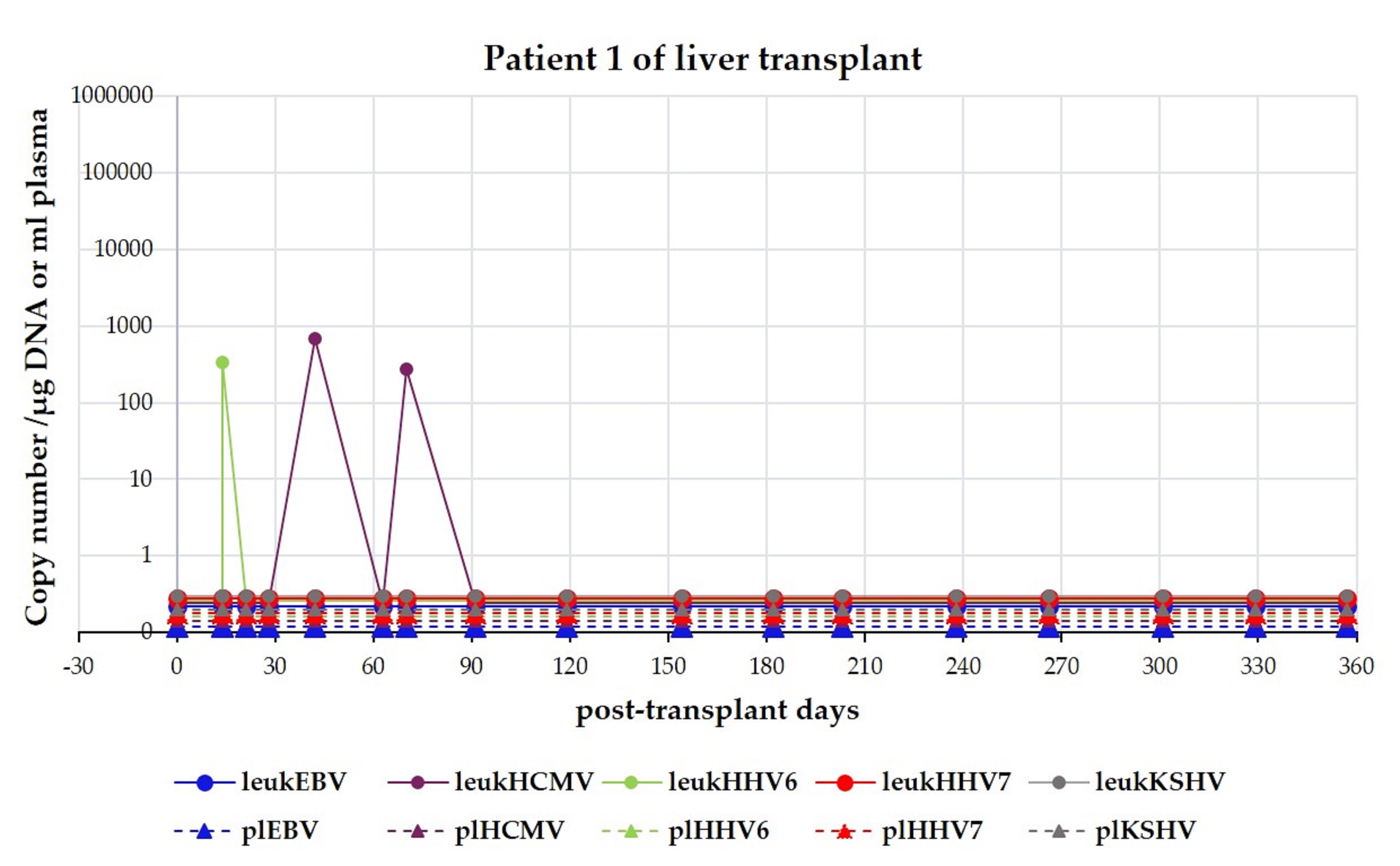

Supplement: Supplementary file 1 [file viruses-10-00730-s001.zip › Supplemntary 3a.jpg]

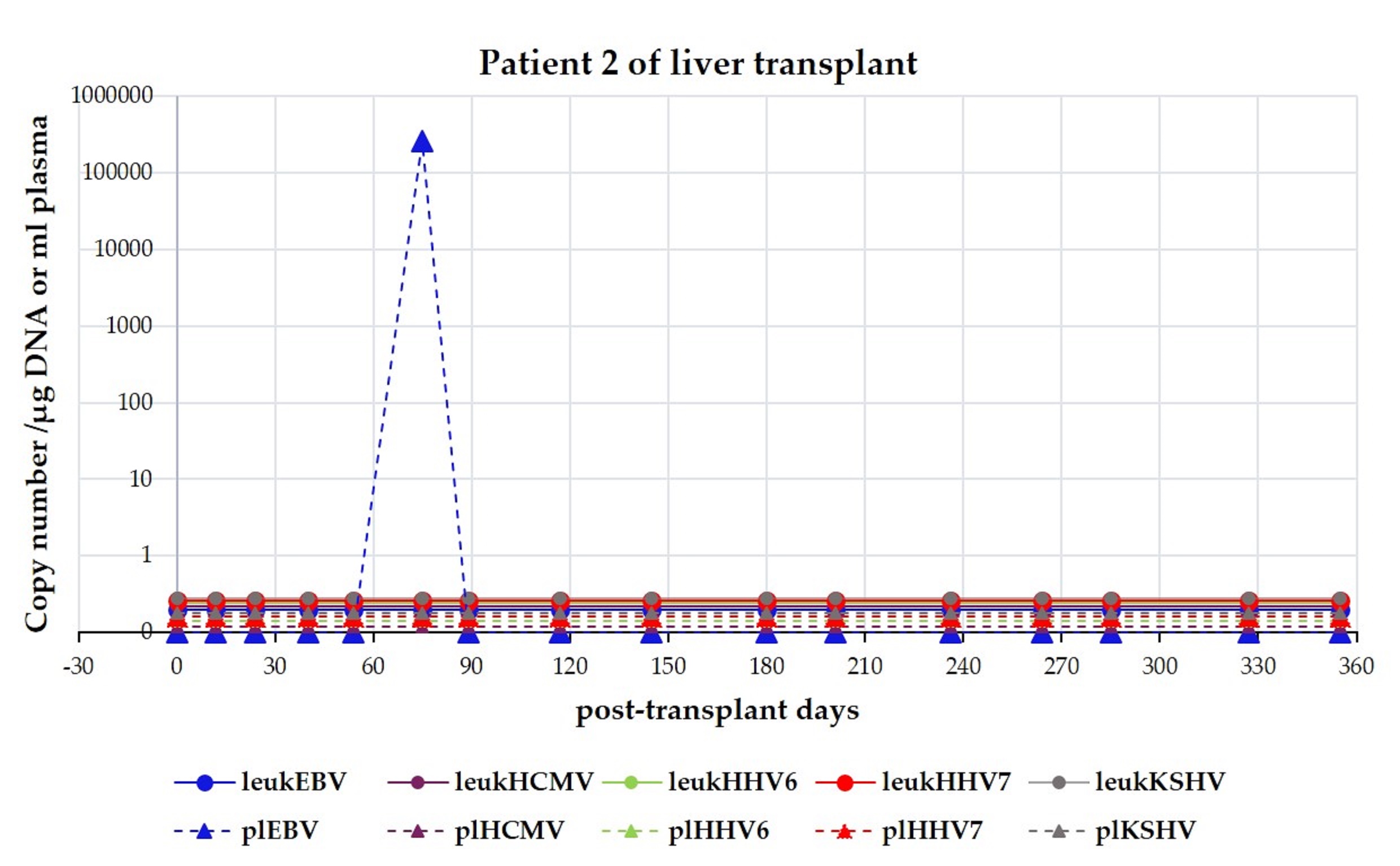

Supplement: Supplementary file 1 [file viruses-10-00730-s001.zip › Supplemntary 3b.jpg]

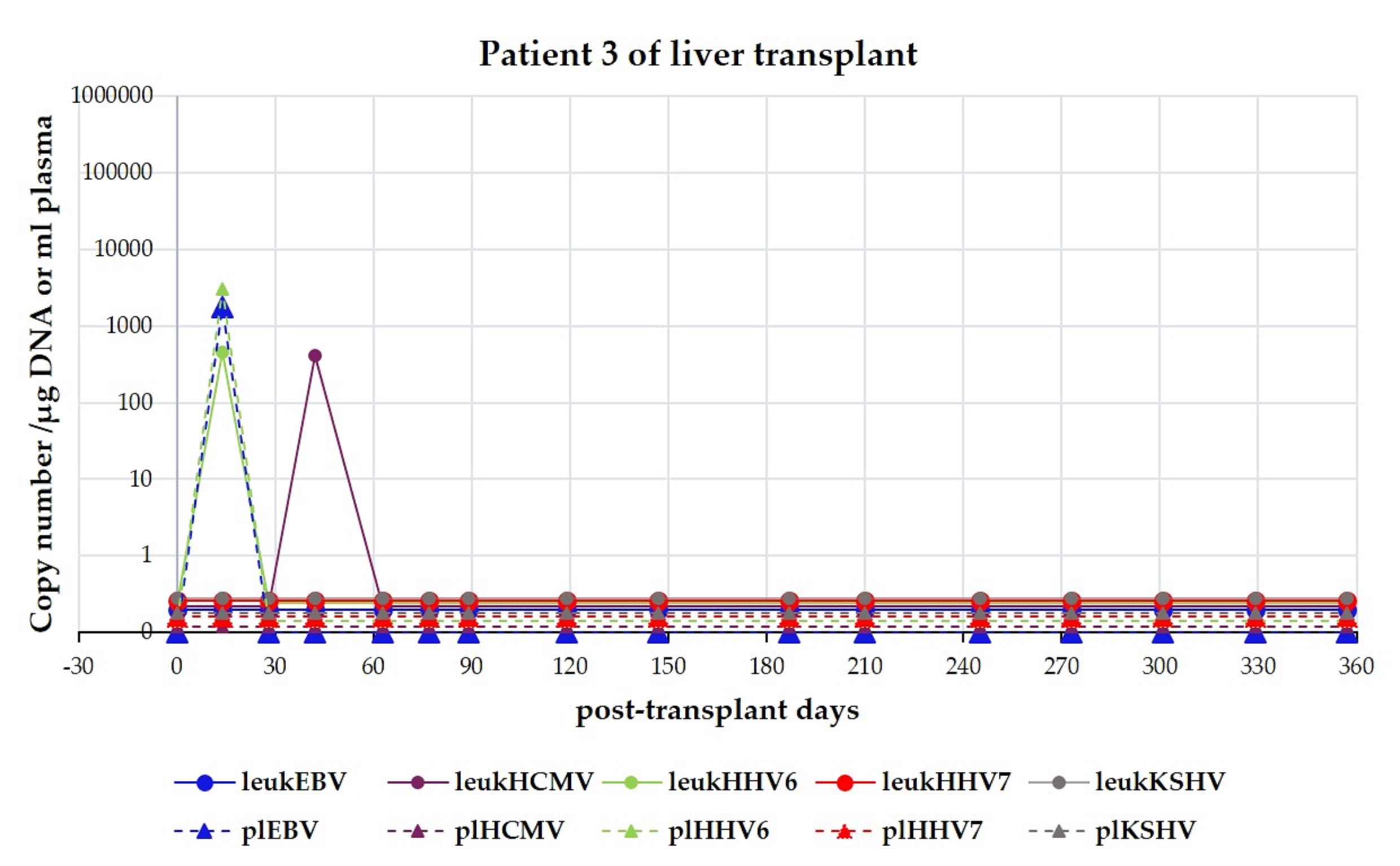

Supplement: Supplementary file 1 [file viruses-10-00730-s001.zip › Supplemntary 3c.jpg]

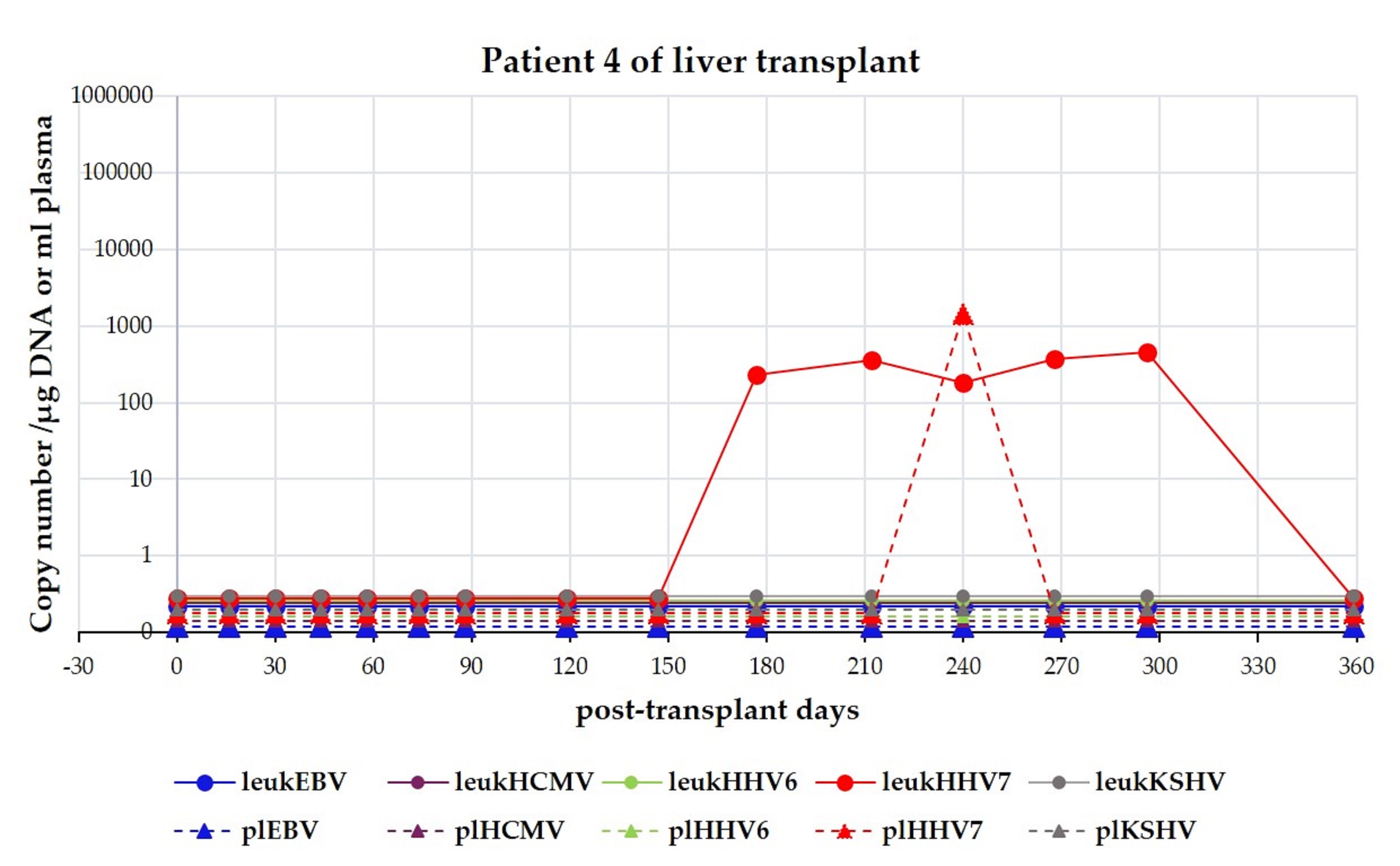

Supplement: Supplementary file 1 [file viruses-10-00730-s001.zip › Supplemntary 3d.jpg]

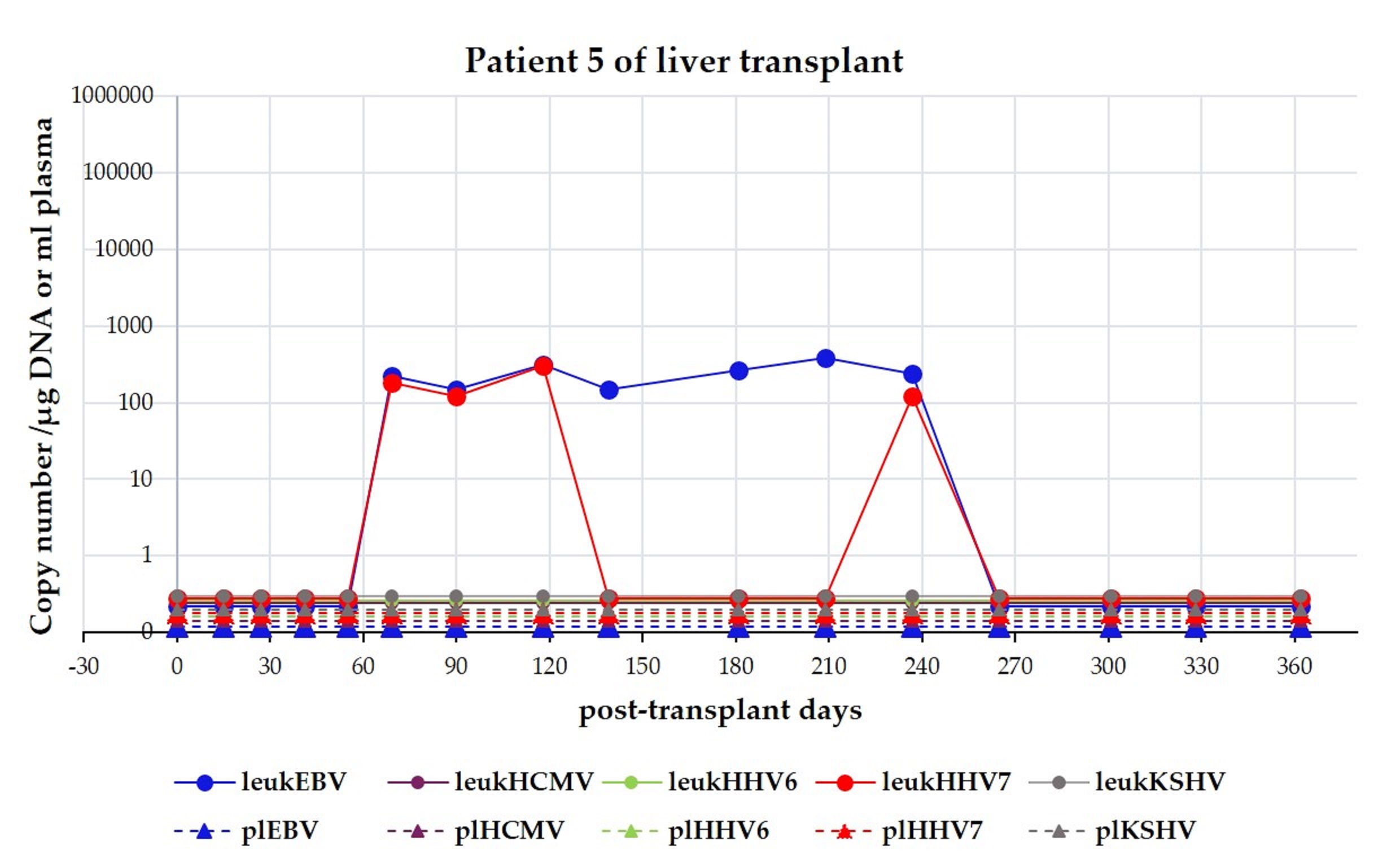

Supplement: Supplementary file 1 [file viruses-10-00730-s001.zip › Supplemntary 3e.jpg]

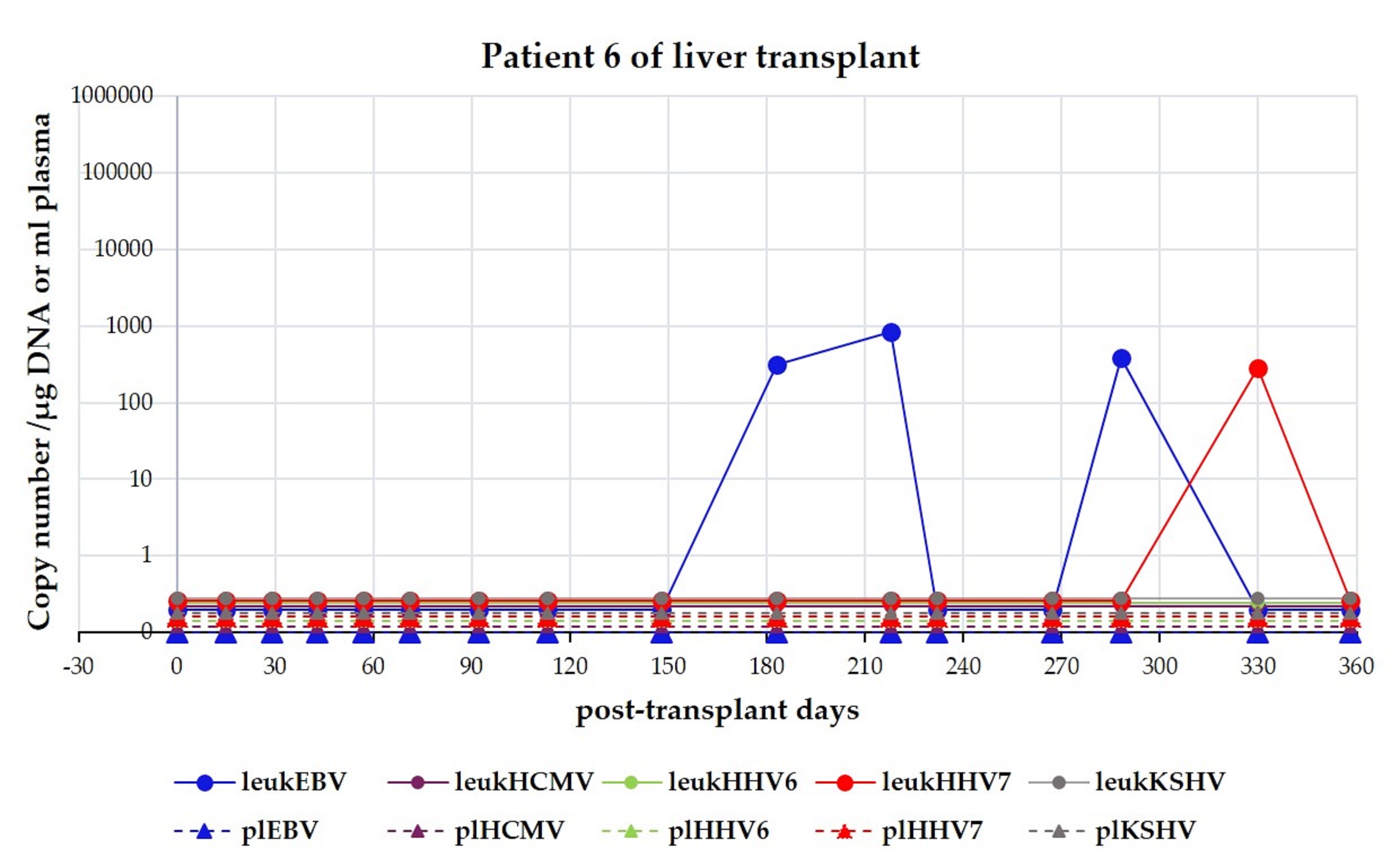

Supplement: Supplementary file 1 [file viruses-10-00730-s001.zip › Supplemntary 3f.jpg]

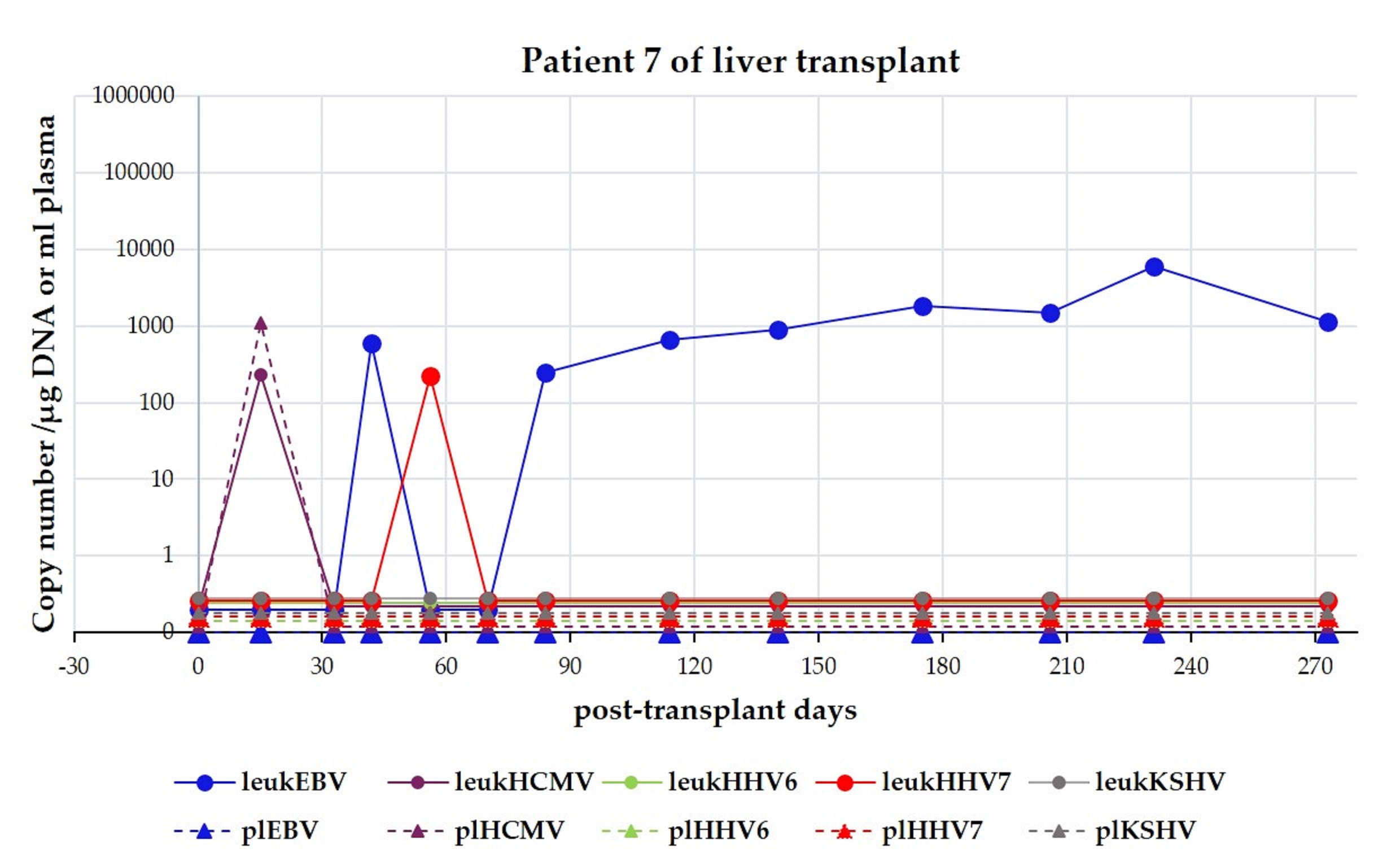

Supplement: Supplementary file 1 [file viruses-10-00730-s001.zip › Supplemntary 3g.jpg]

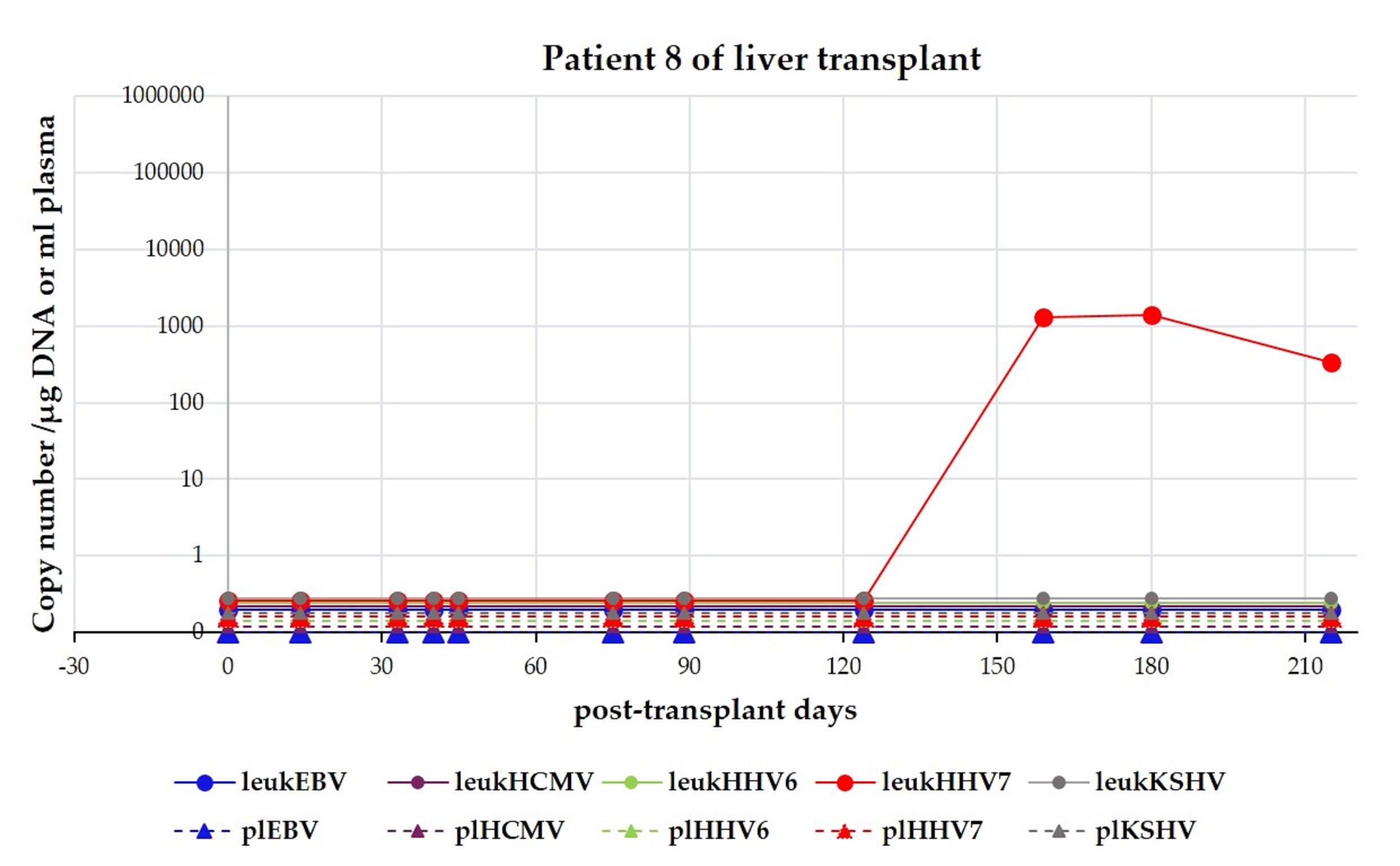

Supplement: Supplementary file 1 [file viruses-10-00730-s001.zip › Supplemntary 3h.jpg]

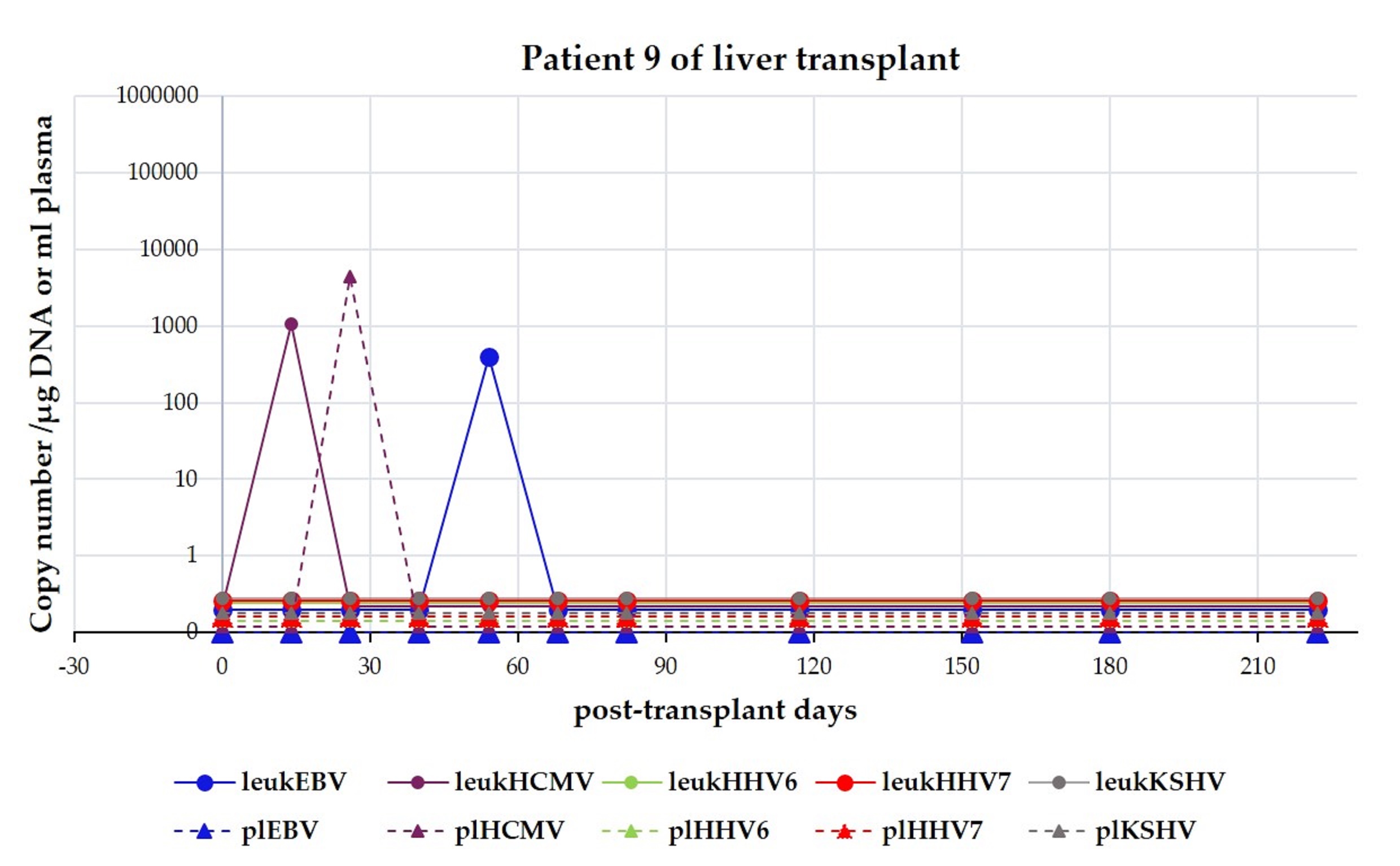

Supplement: Supplementary file 1 [file viruses-10-00730-s001.zip › Supplemntary 3i.jpg]

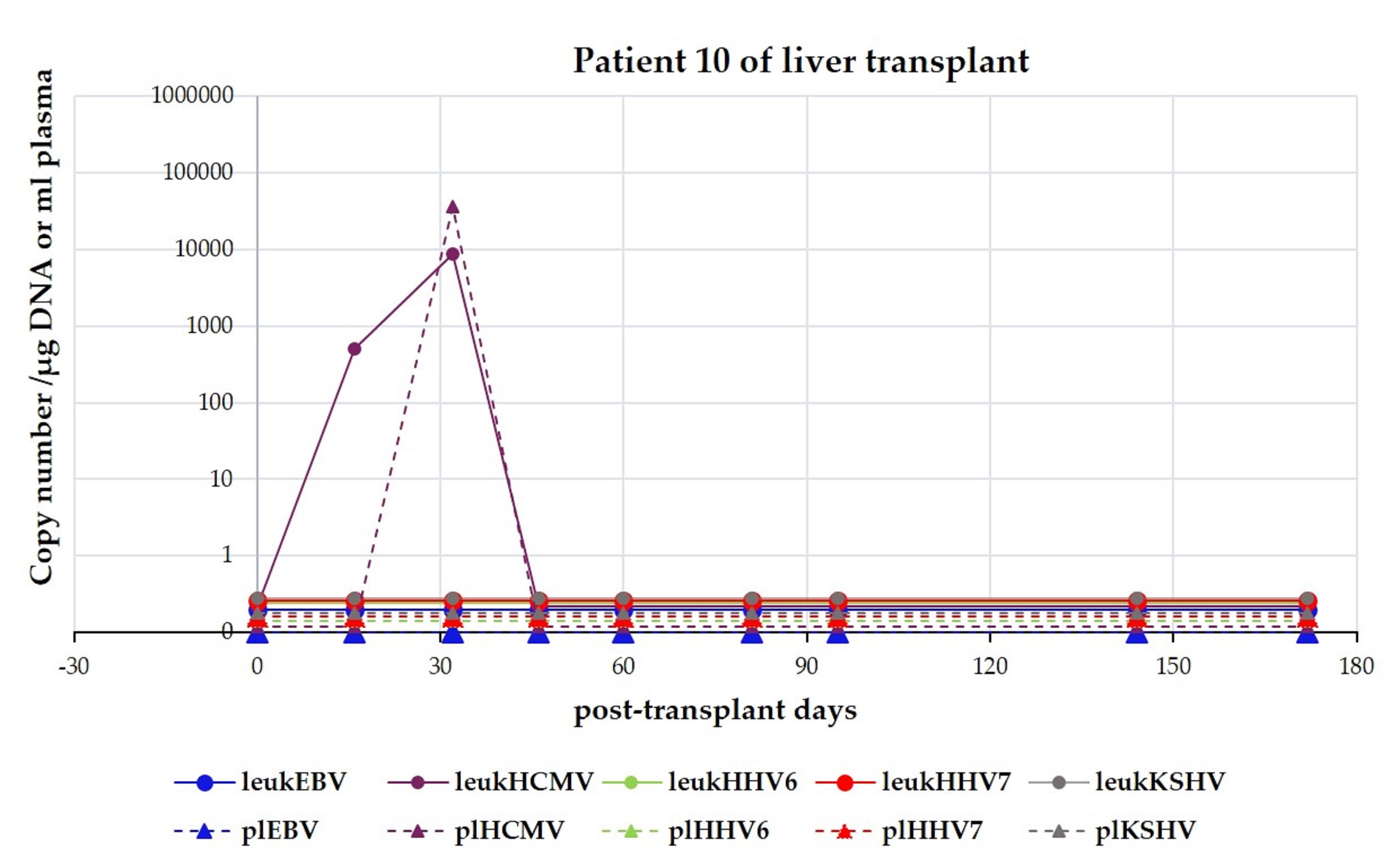

Supplement: Supplementary file 1 [file viruses-10-00730-s001.zip › Supplemntary 3j.jpg]
